# Connecting GSK-3β Inhibitory Activity with IKK-β or ROCK-1 Inhibition to Target Tau Aggregation and Neuroinflammation in Alzheimer’s Disease—Discovery, In Vitro and In Cellulo Activity of Thiazole-Based Inhibitors

**DOI:** 10.3390/molecules29112616

**Published:** 2024-06-02

**Authors:** Izabella Góral, Tomasz Wichur, Emilia Sługocka, Justyna Godyń, Natalia Szałaj, Paula Zaręba, Monika Głuch-Lutwin, Barbara Mordyl, Dawid Panek, Anna Więckowska

**Affiliations:** 1 Department of Physicochemical Drug Analysis, Faculty of Pharmacy, Jagiellonian University Medical College, 9 Medyczna St., 30-688 Krakow, Poland; izabella.goral@doctoral.uj.edu.pl (I.G.); tomasz.wichur@gmail.com (T.W.); emilia.slugocka@doctoral.uj.edu.pl (E.S.); justyna.godyn@uj.edu.pl (J.G.); natalia.guzior@uj.edu.pl (N.S.); paula.zareba@uj.edu.pl (P.Z.); dawid.panek@uj.edu.pl (D.P.); 2 Doctoral School of Medical and Health Sciences, Jagiellonian University Medical College, 16 Lazarza St., 31-530 Krakow, Poland; 3 Department of Pharmacobiology, Faculty of Pharmacy, Jagiellonian University Medical College, 9 Medyczna St., 30-688 Krakow, Poland; monika.gluch-lutwin@uj.edu.pl (M.G.-L.); barbara.mordyl@uj.edu.pl (B.M.)

**Keywords:** GSK-3β, IKK-β, ROCK-1, anti-inflammatory activity, neuroprotective properties Alzheimer’s disease

## Abstract

GSK-3β, IKK-β, and ROCK-1 kinases are implicated in the pathomechanism of Alzheimer’s disease due to their involvement in the misfolding and accumulation of amyloid β (Aβ) and tau proteins, as well as inflammatory processes. Among these kinases, GSK-3β plays the most crucial role. In this study, we present compound **62**, a novel, remarkably potent, competitive GSK-3β inhibitor (IC_50_ = 8 nM, K*_i_* = 2 nM) that also exhibits additional ROCK-1 inhibitory activity (IC_50_ = 2.3 µM) and demonstrates anti-inflammatory and neuroprotective properties. Compound **62** effectively suppresses the production of nitric oxide (NO) and pro-inflammatory cytokines in the lipopolysaccharide-induced model of inflammation in the microglial BV-2 cell line. Furthermore, it shows neuroprotective effects in an okadaic-acid-induced tau hyperphosphorylation cell model of neurodegeneration. The compound also demonstrates the potential for further development, characterized by its chemical and metabolic stability in mouse microsomes and fair solubility.

## 1. Introduction

Dementia is a pathological condition marked by a gradual decline in cognitive functions and accompanied by significant personality changes that hinder daily functioning. Approximately 70% of dementia cases in the elderly can be attributed to Alzheimer’s disease (AD) [1]. The disease ranks among the leading causes of death and poses a substantial challenge to the global economy, primarily due to population ageing [2]. In AD, cognitive deterioration is associated with extensive neurodegenerative changes leading to neuronal loss, particularly in the cerebral cortex and hippocampus [3]. The exact sequence of events leading to these changes is unknown, but misfolding and accumulation of amyloid β (Aβ) and tau proteins and inflammatory processes undoubtedly play a key role in the pathogenesis of the disease [4,5,6,7,8]. 

Based on years of human and animal neuropathological, genetic, and biomarker studies, Aβ is considered an upstream event in the pathomechanism of AD [9]. The Aβ peptide is produced from amyloid precursor protein (APP) in a process dependent on β-secretase (BACE1) and γ-secretase [10]. Once formed, Aβ aggregates into higher-order protein assemblies in the form of highly toxic oligomers, protofibrils, and senile plaques. Their appearance precedes and triggers tau-mediated toxicity that leads to cortical neurodegeneration [11]. An enzyme that is a molecular link between Aβ pathophysiology and tauopathy is Glycogen Synthase Kinase 3β (GSK-3β) [12]. Aβ enhances the activity of GSK-3β, leading to enhanced phosphorylation of tau and resulting in its aggregation into neurofibrillary tangles (NFTs) [13]. Tau is a microtubule-associated protein, and its defect leads to the disturbance of axonal trafficking and eventually to neuron degeneration and death [14]. It was demonstrated both in vitro and in vivo that inhibition of GSK-3β reduces tau hyperphosphorylation and, consequently, restores cognitive deficits [15,16]. At the same time, GSK-3β has a role in the processing of Aβ. One of the mechanisms involved in this process is the upregulation of BACE1 expression via elements of the Nuclear Factor kappa B (NF-κB) pathway [17]. Specific inhibition of GSK signaling attenuates APP cleavage by BACE1, ameliorating memory deficits in animal models [18,19,20]. GSK-3β is a key regulator of processes that underlie the development of Alzheimer’s disease; therefore, the search for its inhibitors has been widely studied [21,22,23,24,25,26]. These efforts led to the discovery of various selective and potent inhibitors, among which several exhibited activity in animal models of AD [27]; however, only one has reached clinical trials in humans to date. Despite excellent research outcomes in animal models, such as significant reductions in tau phosphorylation and Aβ formation, as well as the amelioration of spatial memory deficits, tideglusib was disqualified in phase II due to lack of efficacy in mild–moderate AD patients [28,29,30]. 

Studies have shown that Aβ also increases the activity of ROCK-1 (Rho-associated coiled-coil protein kinase 1) in neurons [31] and that ROCK-1 enhances the BACE1 cleavage of APP, increasing the production of Aβ [32]. Conversely, both in vitro and in vivo depletion of ROCK-1 diminishes the level of Aβ due to reduced APP processing [32] and enhanced lysosomal degradation of APP [33]. ROCK-1 is also implicated in tau protein processing. ROCK-1 inhibitors decrease the phosphorylation of tau in vitro and in vivo as a result of the inactivation of adequate kinases (GSK-3β and Cdk-5) and activation of phosphatases (PPA2A) [34]. Additionally, they upregulate autophagy and proteasomal degradation systems, thus reducing the total amount of tau [34], which was reflected in learning and memory improvements in APP/PS1 mice [32]. The clinically approved ROCK inhibitor, fasudil, suppresses Aβ production in neurons [33,35] and reduces the levels of phosphorylated tau and tau oligomers [34], making ROCK an interesting target in the search for AD treatment.

Aβ also plays a significant role in activating inflammatory processes within the brain [6]. It triggers the release of pro-inflammatory mediators such as interleukin-1β (IL-1β), interleukin-6 (IL-6), tumor necrosis factor-α (TNF-α), nitric oxide (NO), and reactive oxygen species (ROS) by engaging microglial receptors, including CD36, RAGE, and Toll-like receptors (TLRs) [7,36]. The activation of these receptors triggers NF-κB-induced upregulation of NLRP3 and its assembly into the inflammasome, which is essential for the maturation of pro-inflammatory cytokines. NF-κB, a transcription factor protein complex, regulates the expression of genes responsible for the organism’s immune response, thereby playing a crucial role in the neuroinflammatory processes observed in AD [37,38]. Physiologically, NF-κB complexes exist as dimers of various subunits bound by the inhibitory protein IκB. The activation of NF-κB is controlled by the IκB kinase complex (IKK-α, -β), which phosphorylates IκB, leading to the release of the dimer. Subsequently, the released dimer translocates to the nucleus, where it regulates the gene expression of multiple inflammatory pathways [39]. The inhibition of IKKβ (also known as IKK2), with the aim of disrupting the activation of NF-κB, has garnered significant attention in the pursuit of therapies for inflammatory, autoimmune, respiratory, and oncological conditions [40,41,42,43]. In an AD APP-transgenic murine model, IKKβ inhibition proved to be effective in reducing inflammatory microglial activation and decreasing TNF-α, Il-1β, and iNOS inflammatory gene transcription caused by Aβ deposits, thereby improving cognitive functions [44,45]. 

Currently available small-molecule, anti-AD drugs remain ineffective in terminating disease progression [46]; therefore, the exploration of novel, innovative therapeutic areas is still a major priority. The recently FDA-approved monoclonal antibodies are undoubtedly a breakthrough, but they also have limitations—severe side effects and high treatment costs [47,48]. Here, we combined structural elements found in inhibitors of three kinases—GSK-3β, IKK-β, and ROCK-1—to develop multifunctional ligands targeting processes involved in the progression of Alzheimer’s disease. We evaluated their potential in both in vitro and in cellulo settings.

## 2. Design

**Figure 1:**
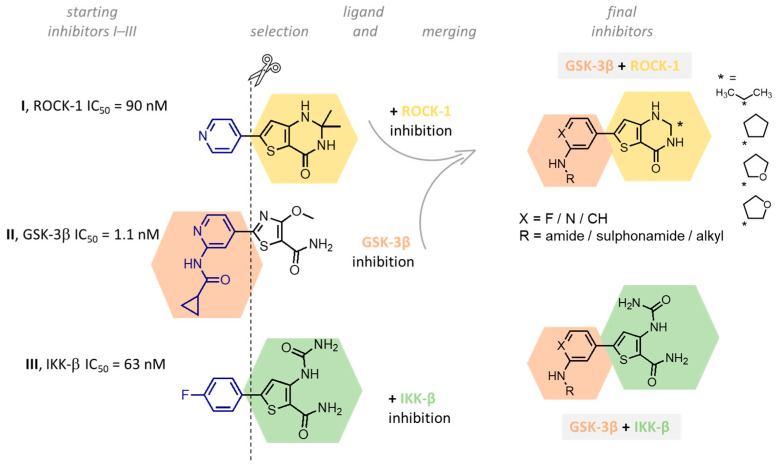
Design of novel ligands targeting ROCK-1, GSK-3β, and IKK-β kinases (* substituent).

We designed new multifunctional ligands based on the structural features of inhibitors targeting three kinases: ROCK-1 (**I**) [49], GSK-3β (**II**) [50], and IKK-β (**III**) [51] (Figure 1). Each compound incorporates two characteristic structural elements crucial for biological activity: a six-membered aromatic ring (either pyridine or 4-fluorobenzene) and a sulfur-based heterocycle containing a carboxamide fragment. To maintain GSK-3β inhibitory activity in all ligands, we utilized the *N*-(pyridin-2-yl)cyclopropanecarboxamide scaffold, which was combined with one of two thiophene ligands, depending on the targeted kinase. For ROCK kinase, we selected the thiophene ring fused with tetrahydropyrimidone, while for IKK-β kinase, we opted for thiophene-urea. To explore the chemical space of the ligands, we varied the substituents attached to the amino group of the 2-aminopyridine ring, resulting in different amides or sulfonamides. Additionally, we replaced the pyridine ring with 4-fluorobenzene or benzene rings. Our attention was also directed towards the cyclic aminal, where we substituted the dimethyl group with cyclopentyl or tetrahydrofuryl moieties.

## 3. Chemistry

**Scheme 1:**
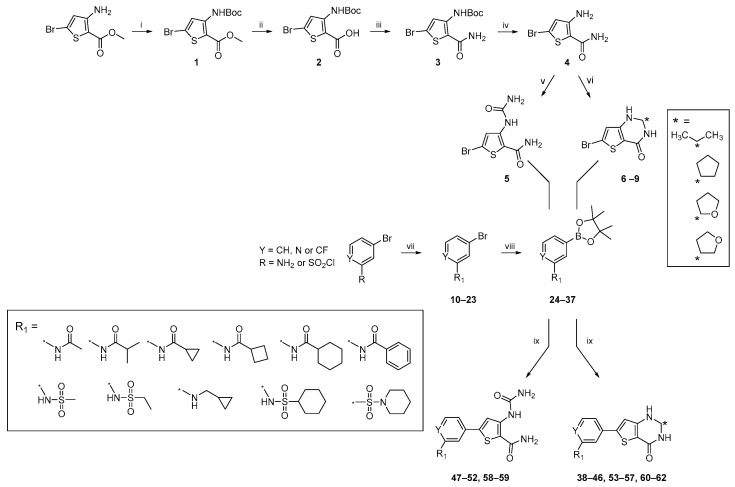
Reagents and conditions: (i) (Boc)_2_O, DMAP, pyridine_(anh.)_, 0 °C–rt, overnight; (ii) 10% KOH_(aq)_, MeOH, reflux, 1 h; (iii) NH_4_HCO_3_, HATU, DIEA, DMF, rt, overnight; (iv) TFA, DCM, rt, 1 h; (v) trichloroacetyl isocyanate, THF_(anh.)_, rt, 1.5 h and 4 M NH_3_ sol. in MeOH, rt, 1.5 h; (vi) *p*-TSA, acetone, CH_3_COOH, 80 °C, overnight or *p*-TSA, appropriate ketone/lactone, toluene_(anh.)_, reflux, overnight; (vii) appropriate acid chloride, pyridine or TEA, DCM, 0 °C–rt, overnight/appropriate sulfonyl chloride, pyridine_(anh.)_, rt, 3 h/appropriate cycloalkyl acid, pyridine_(anh.)_, 50% T_3_P sol. in EtOAc, DCM_(anh.)_, rt, 1 h/NaH (60% in oil), DMF_(anh.)_, 50 °C, 1 h, (bromomethyl)cyclopropane, 100 °C, overnight; (viii) bis(pinacolato)diboron, CH_3_COOK, Pd(dppf)Cl_2_, dioxane_(anh.)_ or DMF_(anh.)_, 80 °C or 100 °C, overnight; (ix) K_2_CO_3_, Pd(dppf)Cl_2_, DMF_(anh.)_, 80 °C, overnight; * substituent.

Thiophene derivatives with urea and tetrahydropyrimidone moieties were obtained in a multistep synthesis, starting from the commercially available methyl 3-amino-5-bromothiophene-2-carboxylate (Scheme 1). The initial step involved the protection of the primary amine group using Boc anhydride, followed by the hydrolysis of an ester group in compound **1** with aqueous KOH, resulting in the formation of compound **2**. Subsequently, the carboxylic group was transformed to primary amide **3** via condensation with NH_4_HCO_3_ using HATU and DIEA. The deprotection of the amino group with TFA resulted in compound **4**, which underwent distinct reactions based on the specific moiety intended for introduction into the thiophene ring. The urea group was introduced in reaction with trichloroacetyl isocyanate, followed by treatment with 4 M ammonia solution in MeOH, yielding compound **5**. Cyclic aminals **6**, **7**, **8,** and **9** were obtained via the condensation of compound **4** with appropriate ketones or lactones. In parallel, pinacol esters **24**–**36** were synthesized starting from sulphonylation, acylation, or alkylation of 4-bromo-2-aminopyridine followed by cross-coupling with bis(pinacolato)diboron in the Miyaura borylation reaction catalyzed by Pd(dppf)Cl_2_. Pinacol ester **37** was prepared in the reaction of piperidine with 3-bromobenzenesulfonyl chloride followed by Miyaura borylation. Final compounds **38**–**62** were obtained in the Suzuki–Miyaura cross-coupling between pinacol esters and thiophene-based bromides under anhydrous conditions with potassium carbonate and Pd(dppf)Cl_2_ catalyst. 

## 4. Biological Evaluation

### 4.1. Inhibitory Activity against GSK-3β, IKK-β, and ROCK-1 Kinases

We evaluated the inhibitory activity of the final compounds against GSK-3β, IKK-β, and ROCK-1 kinases using the commercially available ADP-Glo™ kinase assay [52]. The method was based on the measurement of luminescence that correlates with kinase activity. During the initial screening, the compounds were examined at a concentration of 10 µM, and subsequent IC_50_ determination was performed for those exhibiting over 50% inhibition. The results are shown in Table 1, Table 2 and Table 3. As references in the study, we used a commercially available kinase inhibitor—staurosporine—along with compounds **I** [50], **II** [51], and **III** [52]. The latter are inhibitors of the kinases of interest and were integral to the design of our studies.

SAR analysis of **series I** and **II** included modifications within the carboxamide moiety with the introduction of a variety of alkyl (**38**, **39**, **47**, and **48**), cycloalkyl (**40**–**42** and **49**–**51**), and phenyl (**43** and **52**) substituents, as well as the replacement of carboxamide with a sulfonamide (**44** or **45**) or alkylamine (**46**) group (Table 1). All but one (**47**) carboxamide derivatives displayed GSK-3β inhibitory activity, with IC_50_ values ranging from 10 to 1314 nM. The most potent compounds are those containing isopropyl- (**44** and **48**), cyclopropyl- (**40** and **49**), and cyclobutyl- (**41** and **50**) substituents. Both smaller substituents such as methyl (**38** and **47**) and larger substituents like cyclohexyl (**42** and **51**) and phenyl (**43** and **52**) led to a decrease in activity. The loss of activity was caused by the reduction of an amide to amine (**46**) and the replacement of amide by sulphonamide (**44** or **45**).

**Figure 2:**
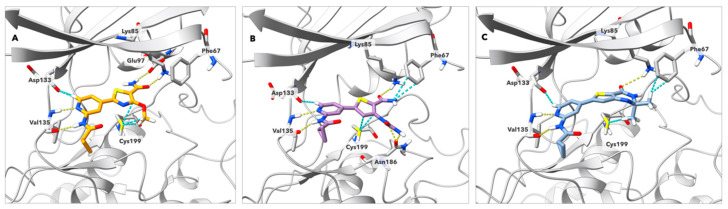
(**A**) Visualization of the **II**/GSK-3β complex of refined crystal structure (PDB ID: 4PTC), gscore function value −9.81. (**B**) Visualization of the docking pose of the **49**/GSK-3β complex, gscore function value −9.87. (**C**) Visualization of the docking pose of the **40**/GSK-3β complex, gscore function value −8.16. All poses were obtained using Glide (Schrodinger Suite, 2023). Residues 52–65 are hidden for clarity. Hydrogen bonds are shown as yellow dashed lines. Favorable contacts (van der Waals overlap > −0.3 Å) are shown as cyan-colored dashed lines.

The efficacy of the compounds is indicative of their binding mode within GSK-3β, as illustrated by **40** and **49** (Figure 2). Their structure is based on a *N*-(piridyn-2-yl)cyclopropanecarboxamide core, inspired by compound **II [51]**, which provides elements crucial for the interactions with the amino acids of the hinge region of the kinase. To support SAR analysis, we employed molecular modelling tools based on the crystal structure of the GSK-3β with the reference compound **II** (PDBID: 4PTC). Crystal structures underwent refinement and energy minimization and served as a model for further docking studies with the Glide procedure (Schrodinger Suite, 2023). The pyridine nitrogen atom in the presented series of ligands serves as a hydrogen bond acceptor (HBA) and the amide NH group as a hydrogen bond donor (HBD), forming hydrogen bonds (HB) with the main chain of Val135. This fragment of the ligands is additionally stabilized by the HB between Asp133 and the C(2) hydrogen atom of the pyridine ring. All three ligands also share a binding pattern with the catalytic Lys85, forming H-bonds with carbonyl oxygen atoms. The ligands exhibit interactions unique to each individual: compound **II** demonstrates stabilization of the methoxy-thiazole spacer group by Cys199, **49** forms hydrogen bonds between the urea fragment and Asn186, and dimethyl substituents of **40** introduce interactions of dispersion character with Phe67 and Cys199. Higher activity of **40** when compared to **49** might be explained by the higher contribution of lipophilic interactions and its more rigid structure, suggesting a lower conformational entropy penalty due to a decreased number of available conformers for the molecule.

Within **series I** and **II**, compounds **38**, **40**, **43**, and **46** displayed inhibitory activity against IKK-β and/or ROCK-1 kinases. Further modifications included the substitution of the pyridine moiety with a phenyl ring (Table 2, **series III** and **IV**). This alteration proved to be advantageous for inhibitory activity against IKK-β, yielding compound **58** with an IC_50_ value of 422 nM, comparable to the reference compound **III** (IC_50_ = 405 nM). On the other hand, the absence of a hydrogen bond acceptor (HBA) in the form of a pyridine nitrogen atom led to a loss of activity towards GSK-3β. This observation confirms the pivotal role of HBA in this fragment. The replacement of the pyridine nitrogen atom with the fluorine atom, as in compounds **54** and **59**, restores the possibility of forming hydrogen bonds with Val135, resulting in GSK-3β inhibition (IC_50_ = 0.453 µM and 1.847 µM, respectively).

Compound **40** was identified in this work as the most potent GSK-3β inhibitor, with an IC_50_ of 10 nM, and had inhibitory potency against IKK-β and ROCK-1 with IC_50_ values of 4.38 µM and 1.76 µM, respectively. Three spirocyclic analogues of compound **40**: **60**, **61**, and **62** were synthesized, as outlined in Table 3. All three analogues retained comparable inhibitory potency against GSK-3β. Notably, compound **60** also preserved its activity against IKK-β and ROCK-1 kinases.

### 4.2. Kinetic Studies of GSK-3β Inhibition by Compound ***62***

As compound **62** was the most potent GSK-3β inhibitor, we selected it for kinetic studies. Non-linear regression analysis of the Michaelis–Menten equation was conducted on ATP-velocity curves to calculate the V_max_ and K_m_ parameters. The analysis revealed increasing K_m_ values alongside preserved V_max_ values with increasing inhibitor concentrations, confirming the ATP-competitive nature of enzyme inhibition by **62**. Lineweaver–Burk and Cornish–Bowden plots, included in the Supporting Information (Figure S1A,B), further illustrate this observation. Kinetic analysis was also employed to determine the K*_i_* value of **62**, obtained through a replot of Lineweaver–Burk plot data as K_m_ versus [I] (refer to Supporting Information, Figure S2). The negative K*_i_* value was directly derived from the plot at the x-axis intersection. The K*_i_* determined through kinetic analysis was marginally lower than the biochemical IC_50_ (2 versus 8 nM).

### 4.3. Kinase Selectivity

**Figure 3:**
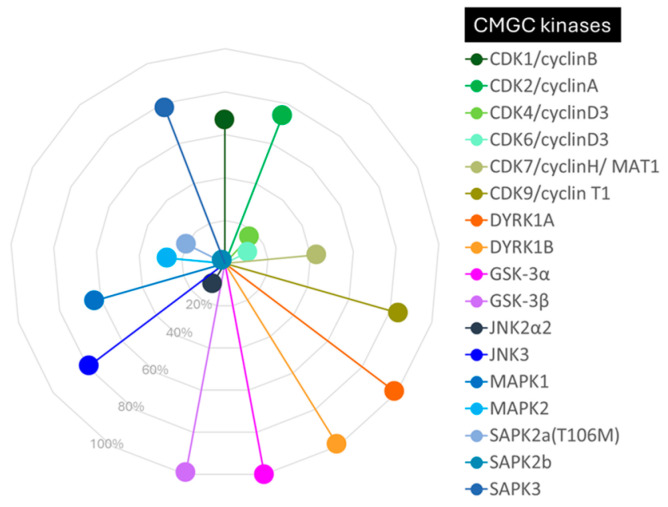
Selectivity of compound **62** against a panel of selected CMGC kinases (CDK1, CDK2, CDK4, CDK6, CDK7, CDK9, DYRK1A, DYRK1B, GSK-3α, GSK-3β, JNK2α2, JNK3, MAPK1, MAPK2, SAPK2a, SAPK2b, and SAPK3). The data are presented as the percentage of kinase inhibition at a concentration of 1 μM of inhibitor **62**.

Compound **62** is an ATP-competitive inhibitor of GSK-3β, raising concerns about selectivity. Therefore, we performed a selectivity screening at Eurofins Discovery on the CMGC group of kinases, which included the CDK, DYRK, MAPK, and GSK families (Figure 3 and Table S1 in the SI). The compound was tested at a concentration of 1 µM, at which we confirmed its activity on the GSK family (99% inhibition). It also displayed similar potency on DYRK kinases (DYRK1A and DYRK1B), which is particularly interesting as this might have a beneficial effect in Alzheimer’s disease, given the role of DYRK in tau and Aβ formation [53,54]. 

### 4.4. Cytotoxicity in HT-22 and BV-2 Cells 

In further studies, we chose the most potent inhibitors selectively targeting GSK-3β (**39**, **41**, **48**–**50**) and IKK-β (**58**), as well as GSK-3β inhibitors with additional ROCK-1 (**62**) and IKK-β/ROCK-1 (**40**, **60**) inhibitory potencies. We assessed their cytotoxic effects at five concentrations (0.1, 1, 10, 50, and 100 µM) in HT-22 (mouse hippocampal neuronal cells) and BV-2 (mouse microglial cells) cell lines using a fluorometric assay with PrestoBlue™ cell viability reagent (Table 4, Table S2 in the SI). Compounds **49** and **50** showed no significant decrease in cell viability in the whole range of the concentrations. Compounds **II**, **39**, **40**, **41**, **48**, **58**, **60**, and **62** decreased cells’ viability, although the IC_50_ values in both cell lines ranged from at least 20-fold times to over 1000-fold times higher than the effective kinases’ inhibitory concentrations. An exception was observed for compounds **40** and **60** with cytotoxic concentrations comparable to effective ROCK-1 and/or IKK-β kinase inhibitory concentrations. In further studies, we only selected compounds that did not decrease the viability of the cells at concentrations up to 10 µM: **49**, **50**, **58**, and **62** (see Table S2 in the SI).

### 4.5. Evaluation of Inhibitory Activity towards Okadaic-Acid-Induced Hyperphosphorylation

**Figure 4:**
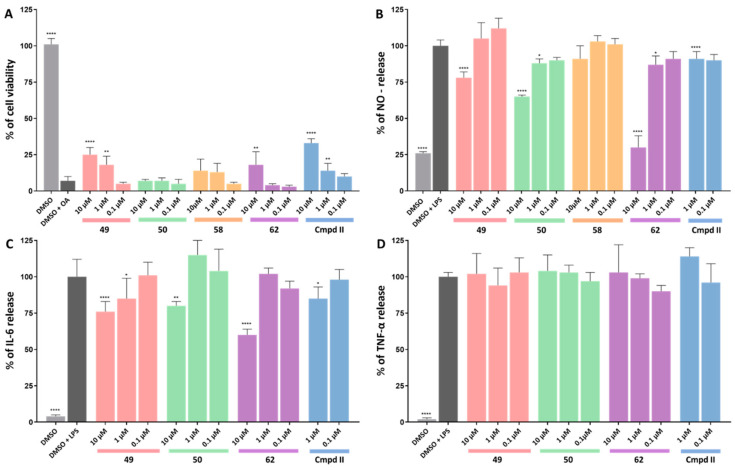
(**A**) Results of the okadaic-acid-induced hyperphosphorylation assay. The HT-22 cells were pretreated for 3 h with okadaic acid (400 nM), and the tested compounds, at the increasing non-cytotoxic concentrations, were determined using a viability assay (or with the vehicle, 10 µM DMSO in the control sample). Cell viability was measured using the PrestoBlue^TM^ cell viability reagent. (**B**–**D**) Results of anti-inflammatory assays in LPS-treated (100 ng/mL) BV-2 cell line. The final compounds and references were tested at increasing non-cytotoxic concentrations and with DMSO as a control sample. NO release was measured using a fluorometric assay with 2,3-diaminonaphthalene (DAN). The IL-6 and TNF-α levels were measured using an LANCE Ultra TR-FRET Detection Kit (Perkin Elmer). Statistical analysis was performed using GraphPad Prism 9.0.0. All values are expressed as mean with SD. Differences among groups were evaluated using one-way ANOVA followed by post hoc analysis (Dunnett’s multiple comparison tests) and were considered statistically significant if *p* < 0.05 (* *p* < 0.05, ** *p* < 0.01, and **** *p* < 0.0001).

We evaluated the neuroprotective properties of the selected compounds in an okadaic-acid-induced tau hyperphosphorylation cell model. Okadaic acid, acting as a phosphatase inhibitor, induces hyperphosphorylation and accumulation of neurofilaments in the cells, thereby mirroring the hyperphosphorylated, tau-induced neurodegeneration observed in AD brain [55,56]. Compounds demonstrating efficacy in the assay enhanced cell viability, likely attributed to the reduction in tau phosphorylation resulting from GSK-3β inhibition. The most significant effect in the assay was displayed by compound **49,** which increased cell viability at 1 and 10 µM, and compound **62,** at 10 µM, as illustrated in Figure 4A. Compounds **50** and **58** were not active.

### 4.6. Evaluation of Anti-Inflammatory Activity in BV-2 Microglial Cells 

Lipopolysaccharide (LPS) is a potent pro-inflammatory stimulus triggering the extensive production of inflammatory mediators, including cytokines and chemokines. Consequently, it finds widespread application in inducing in vitro and in vivo models of sepsis and inflammation, including neuroinflammation. In this study, we employed LPS in the BV-2 cell line and assessed the impact of the selected compounds on the levels of NO, IL-6, and TNF-α production (Figure 4B–D). At a concentration of 10 µM, compounds **49**, **50,** and **62** significantly decreased NO and IL-6 levels. Notably, at 1 µM, compounds **50** and **62** reduced NO levels, while compound **49** reduced IL-6 levels. Moreover, all compounds surpassed the reference compound in these assays. Similar to **II**, none of the compounds exhibited a decrease in TNF-α levels in the assay.

## 5. Preliminary In Vitro ADME

Based on the promising in vitro and in cellulo activity profile, we selected compound **62** for preliminary in vitro ADMET studies, including thermodynamic solubility and chemical and metabolic stability (Table 5). We used compound **II** as a reference in these studies.

### 5.1. Thermodynamic Solubility 

To address potential challenges related to poorly soluble compounds, such as variable and limited intestinal absorption, we conducted thermodynamic solubility studies. Compound **62** exhibited a sixfold increase in solubility when compared to the reference compound **II** (31 µg/mL vs. 5 µg/mL) in Dulbecco’s phosphate-buffered saline (DPBS, Table 5).

### 5.2. Metabolic Stability in Mouse Liver Microsomes

Before conducting the metabolic stability studies, we assessed the chemical stability of **62** and **II** in phosphate buffer at pH 7.4. Following a 120 min incubation at 37 °C, the compounds exhibited 100% stability when compared to the 0 min time point. Subsequently, we evaluated the metabolic stability in mouse liver microsomes (MLM) in the presence of NADPH, with a focus on CYP450-dependent metabolism, utilizing verapamil as a positive control. Notably, the tested compounds demonstrated negligible metabolic degradation after a 15 min incubation, as opposed to verapamil (35% compound remaining after 15 min, Table 5).

## 6. Experimental Section

### 6.1. General Chemistry Information

All reagents were purchased from commercial suppliers and were used without further purification. Tetrahydrofuran (THF) and dichloromethane (DCM) were distilled under argon immediately before use. The drying agent used for THF was sodium/benzophenone ketyl and the drying agent for DCM was calcium hydride. Reactions were monitored using thin-layer chromatography carried out on aluminum sheets precoated with silica gel 60 F254 (Merck, Darmstadt, Germany). Compounds were visualized using UV light and suitable visualization reagents (solution of ninhydrin). Compounds were purified using flash chromatography on Isolera^TM^ Spectra (Biotage, Uppsala, Sweden) with silica gel 60 (63–200 μm; Merck) as a stationary phase or using reverse-phase HPLC performed on LC-4000 Jasco with a Phenomenex Luna C8 (5 μm, 15 × 21.2 mm) column and water/acetonitrile gradient with 0.1% solution of formic acid (*v*/*v*) as a mobile phase. Melting points (mp) were determined in open capillaries on a Büchi B-540 melting point apparatus (Büchi Labortechnik, Flawil, Switzerland). The UPLC-MS analyses were conducted on a UPLC-MS/MS system, comprising a Waters ACQUITY UPLC (Waters Corporation, Milford, MA, USA) coupled with a Waters TQD mass spectrometer (electrospray ionization mode ESI with tandem quadrupole). Chromatographic separations were carried out using the ACQUITY UPLC BEH (bridged ethyl hybrid) C18 column: 2.1 × 100 mm and 1.7 μm particle size. The column was maintained at 40 °C and eluted under gradient conditions using 95%–0% of eluent A over 10 min, at a flow rate of 0.3 mL/min. Eluent A: 0.1% solution of formic acid in water (*v*/*v*). Eluent B: 0.1% solution of formic acid in acetonitrile (*v*/*v*). A total of 10 μL of each sample was injected and chromatograms were recorded using a Waters eλ PDA detector. The UPLC analyses and high-resolution mass spectra (LC-HRMS) were obtained on a Waters ACQUITY I-Class PLUS SYNAPT XS High-Resolution Mass Spectrometer (Waters, Milford, CT, USA) with an MS-Q-TOF detector and a UV–vis-DAD eλ detector. Chromatographic separations were carried out using the same column as for UPLC-MS analyses and with the same applied conditions as mentioned above. The spectra were analyzed in the range of 200–700 nm with 1.2 nm resolution and at a sampling rate of 20 points/s. The UPLC/MS purity of all the test compounds was determined to be ≥ 95% and is given for each compound in the following description. ^1^H NMR and ^13^C NMR spectra were recorded on Varian Mercury 300 MHz (Varian, Inc., Palo Alto, CA, USA) or Jeol 500 MHz (Jeol Inc., Peabody, MA, USA). The chemical shifts are reported in ppm and were referenced to the residual solvent signals (CHLOROFORM-*d*—^1^H: 7.26 ppm, ^13^C: 77.06 ppm; METHANOL-d_4_—^1^H: 3.31 ppm, ^13^C: 49.03 ppm; ACETONE-d_6_—^1^H: 2.05 ppm, ^13^C: 29.82 ppm, 206.03 ppm; and DMSO-d_6_—^1^H: 2.50 ppm, ^13^C: 39.52 ppm); coupling constants are reported in hertz (Hz). HRMS analyses were performed on a MALDI-TOF/TOF mass spectrometer UltrafleXtreme from Bruker Daltonics (Bremen, Germany) with α-cyano-4-hydroxycinnamic acid (CHCA) MALDI matrix after standard dried droplet preparation on a ground steel target plate.

### 6.2. Previously Reported Compounds

*N*-(4-Bromopyridin-2-yl)acetamide (**10**), *N*-(4-bromopyridin-2-yl)cyclopropanecarboxamide (**12**), *N*-(4-bromopyridin-2-yl)cyclobutanecarboxamide (**15**), *N*-(4-bromopyridin-2-yl)cyclohexanecarboxamide (**16**), *N*-(4-bromopyridin-2-yl)benzamide (**17**), *N*-(4-(4,4,5,5-tetramethyl-1,3,2-dioxaborolan-2-yl)pyridin-2-yl)acetamide (**24**), *N*-(4-(4,4,5,5-tetramethyl-1,3,2-dioxaborolan-2-yl)pyridin-2-yl)cyclopropanecarboxamide (**26**), *N*-(4-(4,4,5,5-tetramethyl-1,3,2-dioxaborolan-2-yl)pyridin-2-yl)cyclobutanecarboxamide (**29**), *N*-(4-(4,4,5,5-tetramethyl-1,3,2-dioxaborolan-2-yl)pyridin-2-yl)cyclohexanecarboxamide (**30**), *N*-(4-(4,4,5,5-tetramethyl-1,3,2-dioxaborolan-2-yl)pyridin-2-yl)benzamide (**31**) [57]; *N*-(4-bromopyridin-2-yl)isobutyramide (**11**), *N*-(4-(4,4,5,5-tetramethyl-1,3,2-dioxaborolan-2-yl)pyridin-2-yl)isobutyramide (**25**) [58,59]; *N*-(3-bromophenyl)cyclopropanecarboxamide (**13**), *N*-(3-bromophenyl)ethanesulfonamide (**20**), *N*-(3-(4,4,5,5-tetramethyl-1,3,2-dioxaborolan-2-yl)phenyl)cyclopropanecarboxamide (**27**), *N*-(3-(4,4,5,5-tetramethyl-1,3,2-dioxaborolan-2-yl)phenyl)ethanesulfonamide (**34**) [60]; *N*-(4-bromopyridin-2-yl)methanesulfonamide (**18**) [61]; 1-((3-bromophenyl)sulfonyl)piperidine (**23**), 1-((3-(4,4,5,5-tetramethyl-1,3,2-dioxaborolan-2-yl)phenyl)sulfonyl)piperidine (**37**) [62]; 4-bromo-*N*-(cyclopropylmethyl)pyridin-2-amine (**21**), *N*-(cyclopropylmethyl)-4-(4,4,5,5-tetramethyl-1,3,2-dioxaborolan-2-yl)pyridin-2-amine (**35**) [63]; *N*-(4-(4,4,5,5-tetramethyl-1,3,2-dioxaborolan-2-yl)pyridin-2-yl)methanesulfonamide (**32**) [64,65]; *N*-(3-(4,4,5,5-tetramethyl-1,3,2-dioxaborolan-2-yl)phenyl)cyclohexanesulfonamide (**36**) [66]; 6-bromo-2,2-dimethyl-2,3-dihydrothieno [3,2-*d*]pyrimidin-4(1*H*)-one (**6**) [50]; 5-Bromo-3-ureidothiophene-2-carboxamide (**5**) [67]; and 6′-bromo-1′*H*-spiro[cyclopentane-1,2′-thieno [3,2-*d*]pyrimidin]-4′(3′*H*)-one (**7**) [68].

### 6.3. Chemical Synthesis 

#### 6.3.1. Methyl 5-Bromo-3-((tert-butoxycarbonyl)amino)thiophene-2-carboxylate (**1**)

At rt, 25 mL of anhydrous pyridine DMAP (104 mg, 0.85 mmol, 0.1 equiv.) was added to a stirred solution of methyl 3-amino-5-bromothiophene-2-carboxylate (2.00 g, 8.47 mmol, 1.0 equiv.). Then, the mixture was cooled to 0 °C and, under Ar, di-*tert*-butyl dicarbonate (2.03 g, 9.32 mmol, 1.1 equiv.) was added portion-wise over 20 min. The reaction mixture was warmed up to rt and stirred overnight. After that time, pyridine was evaporated under reduced pressure. The residue was then purified via flash chromatography (PE/EtOAc 95:5). Yield: 2.29 g (80%). ^1^H NMR (500 MHz, CHLOROFORM-*d*) δ ppm: 1.52 (s, 9H), 3.86 (s, 3H), 7.97 (br s, 1H), and 9.33 (br s, 1H). Formula: C_10_H_12_BrNO_4_S.

#### 6.3.2. 5-Bromo-3-((tert-butoxycarbonyl)amino)thiophene-2-carboxylic Acid (**2**)

To a stirred solution of **1** (785 mg, 2.33 mmol), 18 mL of MeOH 10% KOH solution (7 mL) was added and the mixture was heated under reflux for 1 h. Then, the reaction mixture was cooled to rt, the pH was adjusted to 2–3 by adding 6 M of HCl, and the mixture concentrated under vacuum to remove MeOH. The residue was extracted using EtOAc. Then, the combined organic layer was dried over anhydrous Na_2_SO_4_, filtered, and concentrated under vacuum. Yield: 726 mg (97%). ^1^H NMR (500 MHz, DMSO-*d*_6_) δ ppm: 1.44 (s, 6H), 7.78 (s, 1H), 9.37 (s, 1H), and 13.72 (br s, 1H). Formula: C_10_H_12_BrNO_4_S.

#### 6.3.3. *Tert*-Butyl (5-Bromo-2-carbamoylthiophen-3-yl)carbamate (**3**)

To a solution of **2** (726 mg, 2.25 mmol, 1.0 equiv.) in 18 mL of DMF HATU (1.11 g, 2.93 mmol, 1.3 equiv.), DIPEA (1.18 mL, 6.75 mmol, 3.0 equiv.) and NH_4_HCO_3_ (543 mg, 6.75 mmol, 3.0 equiv.) were added and the mixture was stirred at rt overnight. After that time, DMF was evaporated under reduced pressure and the residue was extracted using DCM. The combined organic layer was dried over anhydrous Na_2_SO_4_, filtered, and concentrated under vacuum. The residue was then purified via flash chromatography (DCM/PE/EtOAc 6:3:1). Yield: 621 mg (86%). ^1^H NMR (500 MHz, DMSO-*d*_6_) δ ppm: 1.46 (s, 9H), 7.68 (br s, 2H), 7.81 (s, 1H), and 10.43 (s, 1H). Formula: C_10_H_13_BrN_2_O_3_S.

#### 6.3.4. 3-Amino-5-bromothiophene-2-carboxamide (**4**)

To a solution of **3** (620 mg, 1.93 mmol) in 25 mL DCM TFA (4.5 mL) was added and the mixture was stirred at rt for 1 h. When all of the starting material was consumed, the pH was adjusted to 8 by the addition of saturated aqueous NaHCO_3_ solution and extracted first to DCM and then to EtOAc. The residue was purified by flash chromatography (DCM/PE/EtOAc 5:2:3). Yield: 407 mg (95%). ^1^H NMR (500 MHz, DMSO-*d*_6_) δ ppm 6.56 (s, 2H), 6.70 (s, 1H), 6.92 (br s, 2H). Formula: C_5_H_5_BrN_2_OS.

#### 6.3.5. 5-Bromo-3-ureidothiophene-2-carboxamide (**5**)

A stirred solution of **4** (315 mg, 1.42 mmol) in 13 mL anhydrous THF was cooled to 0 °C on an ice bath and then trichloroacetyl isocyanate (186 µL) was added dropwise. The reaction mixture was warmed up to rt and stirred for 1.5 h. After that time, 4 M of ammonia solution in methanol (20 mL) was added and stirred for another 1.5 h. Then, the solvents were evaporated under reduced pressure and the solid residue was washed with Et_2_O. Yield: 129 mg (34%). ^1^H NMR (500 MHz, DMSO-*d*_6_) δ ppm: 6.64 (br s, 2H), 7.43 (br s, 2H), 7.96 (s, 1H), and 10.01 (s, 1H). Formula: C_6_H_6_BrN_3_O_2_S.

#### 6.3.6. 6-Bromo-2,2-dimethyl-2,3-dihydrothieno [3,2-d]pyrimidin-4(1H)-one (**6**)

To a stirred solution of **4** (200 mg, 0.91 mmol, 1.0 equiv.) in 6 mL acetone, CH_3_COOH (3 mL) and *p*-TSA (17 mg) were added; then, the mixture was heated to 80 °C overnight. After that time, the reaction mixture was cooled to rt, solvents were evaporated under reduced pressure, and the residue was extracted using DCM. The combined organic layer was dried over anhydrous Na_2_SO_4_, filtered, and concentrated under vacuum. Yield: 217 mg (92%). ^1^H NMR (500 MHz, CHLOROFORM-*d*) δ ppm: 1.47 (s, 6H), 6.48 (s, 1H), 6.57 (s, 1H), and 7.24 (s, 1H). Formula: C_8_H_9_BrN_2_OS.

#### 6.3.7. 6′-Bromo-1′H-spiro[cyclopentane-1,2′-thieno [3,2-d]pyrimidin]-4′(3′H)-one (**7**)

A stirred solution of cyclopentanone (3.3 mL) and *p*-TSA (16 mg) in 6 mL of anhydrous toluene was heated to 80 °C. After 5 min, **4** (50 mg, 0.23 mmol) was added, and the mixture was heated to 110 °C for 5 h. Then, the reaction mixture was cooled to rt, the solvent was evaporated under reduced pressure, and the solid residue was washed with MeCN/MeOH 2:1. Yield: 24 mg (37%). ^1^H NMR (500 MHz, DMSO-*d*_6_) δ ppm: 1.58–1.70 (m, 4H), 1.77–1.87 (m, 4H), 6.70 (s, 1H), 7.30 (br s, 1H), and 7.71 (br s, 1H). Formula: C_10_H_11_BrN_2_OS.

#### 6.3.8. 6′-Bromo-4,5-dihydro-1′H,3H-spiro[furan-2,2′-thieno [3,2-d]pyrimidin]-4′(3′H)-one (**8**)

A stirred solution of γ-butyrolactone (6.6 mL) and *p*-TSA (40 mg) in 8 mL of anhydrous toluene was heated to 80 °C. After 5 min, **4** (150 mg, 0.68 mmol) was added, and the mixture was heated to 110 °C overnight. Then, the reaction mixture was cooled to rt and the solvent was evaporated under reduced pressure. The residue was purified via flash chromatography (DCM/MeOH 91:9). Yield: 104 mg (53%). ^1^H NMR (500 MHz, DMSO-*d*_6_) δ ppm: 1.83 (quin, *J* = 6.9 Hz, 2H), 2.66 (t, *J* = 7.6 Hz, 2H), 3.44 (t, *J* = 6.3 Hz, 2H), 4.57 (br s, 1H), 7.56 (s, 1H), and 12.26 (br s, 1H). Formula: C_9_H_9_BrN_2_O_2_S.

#### 6.3.9. 6′-Bromo-4,5-dihydro-1′H,2H-spiro[furan-3,2′-thieno [3,2-d]pyrimidin]-4′(3′H)-one (**9**)

To the tube filled with the solution of **4** (25 mg, 0.11 mmol) in 400 µL anhydrous toluene dihydro-3(2*H*)-furanone (100 µL) was added, sealed and placed in a microwave reactor for 2 h in 120 °C. Then reaction mixture was cooled to rt, solvent was evaporated under reduced pressure. The residue was purified by flash chromatography (DCM/MeOH 93:7). Yield: 30 mg (92%). ^1^H NMR (500 MHz, DMSO-*d*_6_) δ ppm 2.10–2.18 (m, 2H), 3.56 (d, *J* = 8.9 Hz, 1H), 3.73 (d, *J* = 8.9 Hz, 1H), 3.80–3.89 (m, 2H), 6.75 (s, 1H), 7.67 (br s, 1H), 7.97 (br s, 1H). Formula: C_9_H_9_BrN_2_O_2_S.

### 6.4. General Procedure for the Synthesis of Compounds ***10***–***14***, and ***17*** (***GP1***)

An appropriate aromatic amine (1.0 equiv.) was dissolved in DCM, the solution was cooled to 0 °C on an ice bath, and then a base (2.0 equiv.) and an appropriate acid chloride (1.2 equiv.) were added dropwise. The reaction mixture was then warmed up to rt and stirred overnight. After that time, the mixture was extracted using DCM, and the combined organic layer was dried over anhydrous Na_2_SO_4_, filtered, and concentrated under vacuum. The product did not require further purification.

#### 6.4.1. *N*-(4-Bromopyridin-2-yl)acetamide (**10**)

Following **GP1**, compound **10** was prepared using 2-amino-4-bromopyridine (750 mg, 4.33 mmol), acetyl chloride (354 µL, 4.98 mmol), and pyridine (698 µL, 8.66 mmol) in 15 mL of DCM. Yield: 744 mg (79%). ^1^H NMR (500 MHz, CHLOROFORM-*d*) δ ppm: 2.19 (s, 3H), 6.77–6.82 (m, 1H), 7.19 (dd, *J* = 5.4, 1.7 Hz, 1H), 8.05 (d, *J* = 5.4 Hz, 1H), and 8.45 (s, 1H). Formula: C_7_H_7_BrN_2_O.

#### 6.4.2. *N*-(4-Bromopyridin-2-yl)isobutyramide (**11**)

Following **GP1**, compound **11** was prepared using 2-amino-4-bromopyridine (500 mg, 2.89 mmol), isobutyryl chloride (350 µL, 3.32 mmol), and pyridine (466 µL, 5.78 mmol) in 10 mL of DCM. Yield: 430 mg (61%). ^1^H NMR (500 MHz, CHLOROFORM-*d*) δ ppm: 1.24 (d, *J* = 6.9 Hz, 6H), 2.54 (dt, *J* = 13.7, 6.9 Hz, 1H), 7.18 (dd, *J* = 5.2, 1.7 Hz, 1H), 8.05 (d, *J* = 5.4 Hz, 1H), 8.13 (br s, 1H), and 8.50 (d, *J* = 1.4 Hz, 1H). Formula: C_9_H_11_BrN_2_O.

#### 6.4.3. *N*-(4-Bromopyridin-2-yl)cyclopropanecarboxamide (**12**)

Following **GP1**, compound **12** was prepared using 2-amino-4-bromopyridine (2.00 g, 11.56 mmol), cyclopropanecarbonyl chloride (1.21 mL, 13.29 mmol), and pyridine (1.87 mL, 23.12 mmol) in 40 mL of DCM. Yield: 2.54 g (91%). ^1^H NMR (500 MHz, CHLOROFORM-*d*) δ ppm: 0.91–0.96 (m, 2H), 1.08–1.14 (m, 2H), 1.63–1.70 (m, 1H), 7.23 (dd, *J* = 5.7, 1.7 Hz, 1H), 8.02 (d, *J* = 5.7 Hz, 1H), 8.58 (d, *J* = 1.7 Hz, 1H), and 9.61 (br s, 1H). Formula: C_9_H_9_BrN_2_O.

#### 6.4.4. *N*-(3-Bromophenyl)cyclopropanecarboxamide (**13**)

Following **GP1**, compound **13** was prepared using 3-bromoaniline (544 µL, 5.00 mmol), cyclopropanecarbonyl chloride (544 µL, 6.00 mmol), and triethylamine (832 µL, 6.00 mmol) in 5 mL of DCM. Yield: 1100 mg (92%). ^1^H NMR (500 MHz, CHLOROFORM-d) δ ppm: 7.80 (br s, 1H), 7.54 (br s, 1H), 7.38–7.43 (m, 1H), 7.20–7.24 (m, 1H), 7.14–7.19 (m, 1H), 1.47–1.54 (m, 1H), 1.06–1.12 (m, 2H), and 0.84–0.90 (m, 2H). Formula: C_10_H_10_BrNO.

#### 6.4.5. *N*-(5-Bromo-2-fluorophenyl)cyclopropanecarboxamide (**14**)

Following **GP1**, compound **14** was prepared using 5-bromo-2-fluoroaniline (1.00 g, 5.26 mmol), cyclopropanecarbonyl chloride (549 µL, 6.05 mmol), and pyridine (847 µL, 10.52 mmol) in 10 mL of DCM. Yield: 1.32 g (97%). ^1^H NMR (500 MHz, CHLOROFORM-*d*) δ ppm: 0.86–0.91 (m, 2H), 1.07–1.12 (m, 2H), 1.55 (tt, *J* = 7.8, 4.5 Hz, 1H), 6.95 (dd, *J* = 10.7, 8.7 Hz, 1H), 7.12 (ddd, *J* = 8.7, 4.6, 2.4 Hz, 1H), 7.56 (br s, 1H), and 8.55 (br d, *J* = 5.7 Hz, 1H). Formula: C_10_H_9_BrFNO.

#### 6.4.6. *N*-(4-Bromopyridin-2-yl)benzamide (**17**)

Following **GP1**, compound **17** was prepared using 2-amino-4-bromopyridine (500 mg, 2.89 mmol), benzoyl chloride (383 µL, 3.32 mmol), and pyridine (466 µL, 5.78 mmol) in 10 mL of DCM. Yield: 272 mg (34%). ^1^H NMR (500 MHz, DMSO-*d*_6_) δ ppm: 7.41 (dd, *J* = 5.4, 1.7 Hz, 1H), 7.45–7.51 (m, 2H), 7.54–7.60 (m, 1H), 7.94–8.01 (m, 2H), 8.27 (d, *J* = 5.4 Hz, 1H), 8.43 (d, *J* = 1.7 Hz, 1H), and 11.01 (s, 1H). Formula: C_12_H_9_BrN_2_O.

### 6.5. General Procedure for the Synthesis of Compounds ***15***, ***16*** (***GP2***)

An appropriate cycloalkyl acid (1.0 equiv.) was dissolved in anhydrous DCM, and then 2-amino-4-bromopyridine (1.1 equiv.), anhydrous pyridine (2.0 equiv.), and 50% T_3_P sol. in ethyl acetate (2.2 equiv.) were added under Ar. The reaction mixture was stirred for 1 h at rt. After that time, the residue was extracted using DCM. The crude product was purified via flash chromatography.

#### 6.5.1. *N*-(4-Bromopyridin-2-yl)cyclobutanecarboxamide (**15**)

Following **GP2**, compound **15** was prepared using cyclobutanecarboxylic acid (101 µL, 1.06 mmol), 2-amino-4-bromopyridine (200 mg, 1.16 mmol), pyridine (171 µL, 2.12 mmol), and T_3_P (1.37 mL, 2.33 mmol) in 12 mL of DCM. Purification: flash chromatography (DCM/MeOH 98:2). Yield: 268 mg (91%). ^1^H NMR (500 MHz, CHLOROFORM-*d*) δ ppm: 1.86–1.95 (m, 1H), 1.97–2.07 (m, 1H), 2.18–2.29 (m, 2H), 2.31–2.43 (m, 2H), 3.19–3.29 (m, 1H), 7.22 (dd, *J* = 5.4, 1.7 Hz, 1H), 8.04 (d, *J* = 5.4 Hz, 1H), 8.61 (d, *J* = 1.4 Hz, 1H), and 8.82 (br s, 1H). Formula: C_10_H_11_BrN_2_O.

#### 6.5.2. *N*-(4-Bromopyridin-2-yl)cyclohexanecarboxamide (**16**)

Following **GP2**, compound **16** was prepared using cyclohexanecarboxylic acid (506 mg, 3.95 mmol), 2-amino-4-bromopyridine (750 mg, 4.34 mmol), pyridine (636 µL, 7.90 mmol), and T_3_P (5.12 mL, 8.69 mmol) in 30 mL of DCM. Purification: flash chromatography (DCM/MeOH 98:2). Yield: 833 mg (68%). ^1^H NMR (500 MHz, CHLOROFORM-*d*) δ ppm: 1.22–1.37 (m, 3H), 1.53 (qd, *J* = 12.2, 3.2 Hz, 2H), 1.67–1.74 (m, 1H), 1.79–1.87 (m, 2H), 1.96 (br dd, *J* = 13.5, 2.0 Hz, 2H), 2.27 (tt, *J* = 11.7, 3.7 Hz, 1H), 7.20 (dd, *J* = 5.3, 1.9 Hz, 1H), 8.06 (d, *J* = 5.4 Hz, 1H), 8.31 (br s, 1H), and 8.54 (d, *J* = 1.4 Hz, 1H). Formula: C_12_H_15_BrN_2_O.

### 6.6. General Procedure for the Synthesis of Compounds ***18***–***23*** (***GP3***)

An appropriate amine (1.0–1.2 equiv.) was dissolved in anhydrous pyridine or DCM, the solution was cooled to 0 °C on an ice bath, and then sulfonyl chloride (1.0–2.0 equiv.) and base (optional; 1.2–1.5 equiv.) were added dropwise. The reaction mixture was stirred at rt overnight. After that time, pyridine was evaporated under reduced pressure. The crude product was purified using different methods, as described below.

#### 6.6.1. *N*-(4-Bromopyridin-2-yl)methanesulfonamide (**18**)

Following **GP3**, compound **18** was prepared using 2-amino-4-bromopyridine (600 mg, 3.47 mmol) and mesyl chloride (537 µL, 6.94 mmol) in 11 mL of pyridine. Water was added directly to the residue and the solid was filtered. Then, the solid product was washed with Et_2_O. Yield: 530 mg (61%). ^1^H NMR (500 MHz, DMSO-*d*_6_) δ ppm: 3.25 (s, 3H), 7.12 (s, 1H), 7.23 (br d, *J* = 4.6 Hz, 1H), 8.11 (d, *J* = 5.4 Hz, 1H), and 10.91 (br s, 1H). Formula: C_6_H_7_BrN_2_O_2_S.

#### 6.6.2. *N*-(4-Bromopyridin-2-yl)ethanesulfonamide (**19**)

Following **GP3**, compound **19** was prepared using 2-amino-4-bromopyridine (500 mg, 2.89 mmol) and ethanesulfonyl chloride (546 µL, 5.78 mmol) in 9 mL of pyridine. The residue was extracted using DCM. The combined organic layer was dried over anhydrous Na_2_SO_4_, filtered, and concentrated under vacuum. The crude product was then purified via flash chromatography (DCM/MeOH 98:2). Yield: 216 mg (28%). ^1^H NMR (500 MHz, CHLOROFORM-*d*) δ ppm: 1.34–1.40 (m, 3H), 3.21–3.28 (m, 2H), 7.11–7.17 (m, 1H), 7.66 (d, *J* = 1.7 Hz, 1H), and 8.17 (d, *J* = 5.7 Hz, 1H). The proton of the -NH- group was not detected. Formula: C_7_H_9_BrN_2_O_2_S.

#### 6.6.3. *N*-(3-Bromophenyl)ethanesulfonamide (**20**)

Following **GP3**, compound **20** was prepared using 3-bromoaniline (435 µL, 4.00 mmol), ethanesulfonyl chloride (492 µL, 5.20 mmol), and pyridine (484 µL, 6.00 mmol) in 20 mL of DCM. The mixture was transferred to a separatory funnel and washed sequentially with saturated NH_4_Cl_(aq.)_ and saturated NaCl_(aq.)_. The organic layer was dried over anhydrous Na_2_SO_4_, filtered, and concentrated under vacuum. The residue was purified using flash chromatography (DCM/MeOH 98:2 then 96:4). Yield: 940 mg (89%). ^1^H NMR (500 MHz, CHLOROFORM-d) δ ppm: 7.41–7.43 (m, 1H), 7.32 (s, 1H), 7.27–7.30 (m, 1H), 7.18–7.22 (m, 2H), 3.18 (q, *J* = 7.3 Hz, 2H), and 1.39 (t, *J* = 7.4 Hz, 3H). Formula: C_8_H_10_BrNO_2_S.

#### 6.6.4. 4-Bromo-N-(cyclopropylmethyl)pyridin-2-amine (**21**)

To a suspension of NaH (60% suspension in mineral oil, 240 mg, 6.00 mmol, 2.0 equiv.) in DMF (8 mL), 4-bromopyridin-2-amine (519 mg, 3.00 mmol, 1.0 equiv.) was added, the mixture was stirred for 1 h at 50 ºC, and then cyclopropylmethyl bromide (405 mg, 3.00 mmol, 1.0 equiv.) was added. The reaction mixture was stirred overnight at 100 ºC. After that time, the mixture was concentrated under vacuum, diluted with water, and extracted using DCM. The combined organic layer was dried over anhydrous Na_2_SO_4_, filtered, and concentrated under vacuum. The residue was purified via flash chromatography (PE/EtOAc 8:2). Yield: 192 mg (28%). ^1^H NMR (500 MHz, CHLOROFORM-*d*) δ ppm: 7.88 (d, *J* = 5.4 Hz, 1 H), 6.71 (dd, *J* = 5.4, 1.7 Hz, 1 H), 6.56 (d, *J* = 1.4 Hz, 1 H), 4.89 (br s, 1 H), 3.10 (dd, *J* = 7.0, 5.3 Hz, 2 H), 1.02–1.14 (m, 1 H), 0.51–0.60 (m, 2 H), and 0.21–0.29 (m, 2 H). Formula: C_9_H_11_BrN_2_.

#### 6.6.5. *N*-(3-Bromophenyl)cyclohexanesulfonamide (**22**)

Following **GP3**, compound **22** was prepared using 3-bromoaniline (163 mL, 1.50 mmol), cyclohexylsulfonyl chloride (283 mL, 1.95 mmol), and pyridine (182 mL, 2.25 mmol) in 7.5 mL of DCM. The mixture was transferred to a separatory funnel and washed sequentially with saturated NH_4_Cl_(aq.)_ and saturated NaCl_(aq.)_. The organic layer was dried over anhydrous Na_2_SO_4_, filtered, and concentrated under vacuum. The residue was purified using flash chromatography (0–4% MeOH in DCM). Yield: 425 mg (98%). ^1^H NMR (500 MHz, CHLOROFORM-*d*) δ ppm: 7.38 (t, *J* = 2.0 Hz, 1H), 7.23–7.27 (m, 1H), 7.12–7.20 (m, 2H), 6.78 (s, 1H), 3.01 (tt, *J* = 12.2, 3.4 Hz, 1H), 2.10–2.19 (m, 2H), 1.82–1.92 (m, 2H), 1.63–1.72 (m, 1H), 1.46–1.62 (m, 2H), and 1.10–1.30 (m, 3H). Formula: C_12_H_16_BrNO_2_S.

#### 6.6.6. 1-((3-Bromophenyl)sulfonyl)piperidine (**23**)

Following **GP3**, compound **23** was prepared using piperidine (475 µL, 4.80 mmol), 3-bromobenzene-1-sulfonyl chloride (577 µL, 4.00 mmol), and triethylamine (665 µL, 4.80 mmol) in 4.5 mL of DCM. The mixture was transferred to a separatory funnel and washed sequentially with saturated NH_4_Cl_(aq.)_ and saturated NaCl_(aq.)_. The organic layer was dried over anhydrous Na_2_SO_4_, filtered, and concentrated under vacuum. Yield: 1.08 g (88%). ^1^H NMR (500 MHz, CHLOROFORM-d) δ ppm: 7.91 (t, *J* = 1.9 Hz, 1H), 7.67–7.75 (m, 2H), 7.42 (t, *J* = 7.9 Hz, 1H), 2.96–3.06 (m, 4H), 1.63–1.71 (m, 4H), and 1.41–1.49 (m, 2H). Formula: C_11_H_14_BrNO_2_S.

### 6.7. General Procedure for the Synthesis of Compounds ***24***–***37*** (***GP4***)

An appropriate aryl bromide (1.0 equiv.) and bis(pinacolato)diboron (1.0–1.5 equiv.) were dissolved in anhydrous dioxane. Then, potassium acetate (2.0 equiv.) was added and, under Ar, Pd(dppf)Cl_2_ (0.1–0.4 equiv.) was added. The reaction was stirred under reflux overnight. After that time, the reaction mixture was diluted with DCM, filtered through Celite, and evaporated under reduced pressure. The residue was dissolved in EtOAc. Then, activated charcoal (5–10 g) was added and the mixture was stirred under reflux for 1 h. The mixture was then filtered again through Celite and evaporated under reduced pressure. The crude product was purified using different methods, as described below.

#### 6.7.1. *N*-(4-(4,4,5,5-Tetramethyl-1,3,2-dioxaborolan-2-yl)pyridin-2-yl)acetamide (**24**)

Following **GP4**, compound **24** was prepared using *N*-(4-bromopyridin-2-yl)acetamide (**10**) (744 mg, 3.46 mmol), bis(pinacolato)diboron (879 mg, 3.46 mmol), potassium acetate (679 mg, 6.92 mmol), Pd(dppf)Cl_2_ (255 mg, 0.35 mmol) in 10 mL dioxane. Purification: crystallization from EtOAc/PE 1:2. Yield: 520 mg (57%). ^1^H NMR (500 MHz, CHLOROFORM-*d*) δ ppm 1.35 (s, 12H), 2.22 (s, 3H), 7.40 (d, *J* = 4.6 Hz, 1H), 8.26 (br d, *J* = 4.6 Hz, 1H), 8.55 (br s, 1H), 8.79 (br s, 1H). Formula: C_13_H_19_BN_2_O_3_.

#### 6.7.2. *N*-(4-(4,4,5,5-Tetramethyl-1,3,2-dioxaborolan-2-yl)pyridin-2-yl)isobutyramide (**25**)

Following **GP4**, compound **25** was prepared using *N*-(4-bromopyridin-2-yl)isobutyramide (**11**) (430 mg, 1.77 mmol), bis(pinacolato)diboron (450 mg, 1.77 mmol), potassium acetate (348 mg, 3.54 mmol), and Pd(dppf)Cl_2_ (130 mg, 0.18 mmol) in 5 mL of dioxane. Purification: crystallization from EtOAc/PE 1:2. Yield: 200 mg (39%). ^1^H NMR (500 MHz, CHLOROFORM-*d*) δ ppm: 1.24 (d, *J* = 6.9 Hz, 6H), 1.31 (s, 12H), 2.47–2.58 (m, 1H), 7.34 (d, *J* = 4.6 Hz, 1H), 8.19 (br s, 1H), 8.25 (br d, *J* = 4.6 Hz, 1H), and 8.57 (s, 1H). Formula: C_15_H_23_BN_2_O_3_.

#### 6.7.3. *N*-(4-(4,4,5,5-Tetramethyl-1,3,2-dioxaborolan-2-yl)pyridin-2-yl)cyclopropanecarboxamide (**26**)

Following **GP4**, compound **26** was prepared using *N*-(4-bromopyridin-2-yl)cyclopropanecarboxamide (**12**) (2.54 g, 8.82 mmol), bis(pinacolato)diboron (2.54 g, 8.82 mmol), potassium acetate (1.73 g, 17.64 mmol), and Pd(dppf)Cl_2_ (650 mg, 0.89 mmol) in 21 mL of dioxane. Purification: crystallization from EtOAc/PE 1:2. Yield: 1.03 g (40%). ^1^H NMR (500 MHz, CHLOROFORM-*d*) δ ppm: 0.83–0.90 (m, 2H), 1.07–1.12 (m, 2H), 1.30 (s, 12H), 1.55–1.64 (m, 1H), 7.34 (d, *J* = 4.6 Hz, 1H), 8.24 (d, *J* = 4.6 Hz, 1H), 8.55 (s, 1H), and 8.95 (br s, 1H). Formula: C_15_H_21_BN_2_O_3_.

#### 6.7.4. *N*-(3-(4,4,5,5-Tetramethyl-1,3,2-dioxaborolan-2-yl)phenyl)cyclopropanecarboxamide (**27**)

Following **GP4**, compound **27** was prepared using *N*-(3-bromophenyl)cyclopropanecarboxamide (**13**) (600 mg, 2.50 mmol), bis(pinacolato)diboron (635 mg, 2.50 mmol), potassium acetate (491 mg, 5.00 mmol), and Pd(dppf)Cl_2_ (180 mg, 0.25 mmol) in 7 mL of dioxane. Purification: crystallization from EtOAc/PE 1:2. Yield: 475 mg (66%). ^1^H NMR (500 MHz, CHLOROFORM-*d*) δ ppm: 0.82 (dq, *J* = 7.6, 3.7 Hz, 2H), 1.03–1.10 (m, 2H), 1.32 (s, 12H), 1.44 (m, *J* = 2.3 Hz, 1H), 7.28–7.34 (m, 1H), 7.37 (br s, 1H), 7.51 (br d, *J* = 7.2 Hz, 1H), 7.69 (s, 1H), and 7.83 (br d, *J* = 7.4 Hz, 1H). Formula: C_16_H_22_BNO_3_.

#### 6.7.5. *N*-(2-Fluoro-5-(4,4,5,5-tetramethyl-1,3,2-dioxaborolan-2-yl)phenyl)cyclopropanecarboxamide (**28**)

Following **GP4**, compound **28** was prepared using *N*-(5-bromo-2-fluorophenyl)cyclopropanecarboxamide (**14**) (660 mg, 2.56 mmol), bis(pinacolato)diboron (975 mg, 3.84 mmol), potassium acetate (754 mg, 7.68 mmol), and Pd(dppf)Cl_2_ (180 mg, 0.25 mmol) in 7 mL of dioxane. Purification: crystallization from EtOAc/PE 1:2. Yield: 291 mg (37%). ^1^H NMR (500 MHz, CHLOROFORM-*d*) δ ppm: 0.83–0.88 (m, 2H), 1.06–1.13 (m, 2H), 1.29 (s, 12H), 1.50–1.58 (m, 1H), 7.06 (dd, *J* = 11.2, 8.3 Hz, 1H), 7.47 (br t, *J* = 6.4 Hz, 1H), 7.52 (br s, 1H), and 8.67 (br d, *J* = 7.7 Hz, 1H). Formula: C_16_H_21_BFNO_3_.

#### 6.7.6. *N*-(4-(4,4,5,5-Tetramethyl-1,3,2-dioxaborolan-2-yl)pyridin-2-yl)cyclobutanecarboxamide (**29**)

Following **GP4**, compound **29** was prepared using *N*-(4-bromopyridin-2-yl)cyclobutanecarboxamide (**15**) (405 mg, 1.59 mmol), bis(pinacolato)diboron (404 mg, 1.59 mmol), potassium acetate (312 mg, 3.18 mmol), and Pd(dppf)Cl_2_ (120 mg, 0.16 mmol) in 5 mL of dioxane. Purification: crystallization from EtOAc/PE 1:2. Yield: 120 mg (25%). ^1^H NMR (500 MHz, CHLOROFORM-*d*) δ ppm 1.31 (s, 12H), 1.87–1.96 (m, 1H), 1.97–2.04 (m, 1H), 2.19–2.28 (m, 2H), 2.34–2.44 (m, 2H), 3.21 (quin, *J* = 8.4 Hz, 1H), 7.37 (d, *J* = 4.9 Hz, 1H), 8.22 (d, *J* = 4.9 Hz, 1H), 8.38 (br s, 1H), and 8.63 (s, 1H). Formula: C_16_H_23_BN_2_O_3_.

#### 6.7.7. *N*-(4-(4,4,5,5-Tetramethyl-1,3,2-dioxaborolan-2-yl)pyridin-2-yl)cyclohexanecarboxamide (**30**)

Following **GP4**, compound **30** was prepared using *N*-(4-bromopyridin-2-yl)cyclohexanecarboxamide (**16**) (831 mg, 2.93 mmol), bis(pinacolato)diboron (744 mg, 2.93 mmol), potassium acetate (575 mg, 5.86 mmol), and Pd(dppf)Cl_2_ (215 mg, 0.29 mmol) in 8.5 mL of dioxane. Purification: crystallization from EtOAc/PE 1:2. Yield: 602 mg (62%). ^1^H NMR (500 MHz, CHLOROFORM-*d*) δ ppm: 1.22–1.29 (m, 2H), 1.32 (s, 12H), 1.34 (s, 1H), 1.48–1.61 (m, 2H), 1.69 (br d, *J* = 12.0 Hz, 1H), 1.77–1.87 (m, 2H), 1.97 (br d, *J* = 12.6 Hz, 2H), 2.32 (br t, *J* = 11.0 Hz, 1H), 7.39 (d, *J* = 4.6 Hz, 1H), 8.21 (d, *J* = 4.9 Hz, 1H), 8.67 (s, 1H), and 8.78 (br s, 1H). Formula: C_18_H_27_BN_2_O_3_.

#### 6.7.8. *N*-(4-(4,4,5,5-Tetramethyl-1,3,2-dioxaborolan-2-yl)pyridin-2-yl)benzamide (**31**)

Following **GP4**, compound **31** was prepared using *N*-(4-bromopyridin-2-yl)benzamide (**17**) (398 mg, 1.44 mmol), bis(pinacolato)diboron (366 mg, 1.44 mmol), potassium acetate (283 mg, 2.88 mmol), and Pd(dppf)Cl_2_ (110 mg, 0.15 mmol) in 4 mL of dioxane. Purification: crystallization from EtOAc/PE 1:2. Yield: 195 mg (42%). ^1^H NMR (500 MHz, CHLOROFORM-*d*) δ ppm: 1.35 (s, 12H), 7.44 (d, *J* = 4.0 Hz, 1H), 7.47–7.53 (m, 2H), 7.55–7.60 (m, 1H), 7.99 (d, *J* = 7.4 Hz, 2H), 8.27 (br d, *J* = 4.3 Hz, 1H), 8.81 (s, 1H), and 9.22 (br s, 1H). Formula: C_18_H_21_BN_2_O_3_.

#### 6.7.9. *N*-(4-(4,4,5,5-Tetramethyl-1,3,2-dioxaborolan-2-yl)pyridin-2-yl)methanesulfonamide (**32**)

Following **GP4**, compound **32** was prepared using *N*-(4-bromopyridin-2-yl)methanesulfonamide (**18**) (518 mg, 2.06 mmol), bis(pinacolato)diboron (680 mg, 2.68 mmol), potassium acetate (404 mg, 4.12 mmol), and Pd(dppf)Cl_2_ (240 mg, 0.33 mmol) in 6 mL of dioxane. The solid product was used further without purification. Formula: C_12_H_19_BN_2_O_4_S. MW: 298.16. LC-MS: *m*/*z* 217 [M + H]^+^ of (2-(methylsulfonamido)pyridin-4-yl)boronic acid, MW: 216.02.

#### 6.7.10. *N*-(4-(4,4,5,5-Tetramethyl-1,3,2-dioxaborolan-2-yl)pyridin-2-yl)ethanesulfonamide (**33**)

Following **GP4**, compound **33** was prepared using *N*-(4-bromopyridin-2-yl)ethanesulfonamide (**19**) (360 mg, 1.36 mmol), bis(pinacolato)diboron (518 mg, 2.04 mmol), potassium acetate (267 mg, 2.72 mmol), and Pd(dppf)Cl_2_ (200 mg, 0.27 mmol) in 5 mL of dioxane. Purification: crystallization from EtOAc/PE 1:2. Yield: 241 mg (57%). ^1^H NMR (500 MHz, CHLOROFORM-*d*) δ ppm: 1.32 (s, 12H), 1.34–1.38 (m, 3H), 3.26 (q, *J* = 7.4 Hz, 2H), 7.25 (s, 1H), 7.69 (s, 1H), and 8.25 (d, *J* = 5.2 Hz, 1H). The proton of the -NH- group was not detected. Formula: C_13_H_21_BN_2_O_4_S.

#### 6.7.11. *N*-(3-(4,4,5,5-Tetramethyl-1,3,2-dioxaborolan-2-yl)phenyl)ethanesulfonamide (**34**)

Following **GP4**, compound **34** was prepared using *N*-(3-bromophenyl)ethanesulfonamide (**20**) (528 mg, 2.00 mmol), bis(pinacolato)diboron (762 mg, 3.00 mmol), potassium acetate (393 mg, 4.00 mmol), and Pd(dppf)Cl_2_ (294 mg, 0.40 mmol) in 7.5 mL of dioxane. Purification: crystallization from the mixture of Et_2_O and EtOAc. Yield: 433 mg (70%). ^1^H NMR (500 MHz, CHLOROFORM-d) δ ppm: 7.62 (dt, *J* = 7.3, 1.1 Hz, 1H), 7.49–7.51 (m, 1H), 7.43–7.47 (m, 1H), 7.34–7.39 (m, 1H), 6.41 (s, 1H), 3.13 (q, *J* = 7.4 Hz, 2H), 1.36–1.40 (m, 3H), and 1.35 (s, 12H). Formula: C_14_H_22_BNO_4_S.

#### 6.7.12. *N*-(Cyclopropylmethyl)-4-(4,4,5,5-tetramethyl-1,3,2-dioxaborolan-2-yl)pyridin-2-amine (**35**)

Following **GP4**, compound **35** was prepared using 4-bromo-*N*-(cyclopropylmethyl)pyridin-2-amine (**21**) (242 mg, 1.07 mmol), bis(pinacolato)diboron (259 mg, 1.07 mmol), potassium acetate (210 mg, 2.14 mmol), and Pd(dppf)Cl_2_ (79 mg, 0.11 mmol) in 4 mL of dioxane. Purification: crystallization from the mixture of PE and EtOAc. Yield: 260 mg (89%). Formula: C_15_H_23_BN_2_O_2_. MW: 274.17.

#### 6.7.13. *N*-(3-(4,4,5,5-Tetramethyl-1,3,2-dioxaborolan-2-yl)phenyl)cyclohexanesulfonamide (**36**)

Following **GP4**, compound **36** was prepared using *N*-(3-bromophenyl)cyclohexanesulfonamide (**22**) (416 mg, 1.31 mmol), bis(pinacolato)diboron (500 mg, 1.97 mmol), potassium acetate (257 mg, 2.62 mmol), and Pd(dppf)Cl_2_ (200 mg, 0.27 mmol) in 5 mL of dioxane. Purification: flash chromatography (0–3% MeOH gradient in DCM). Yield: 415 mg (87%). ^1^H NMR (500 MHz, CHLOROFORM-*d*) δ ppm: 7.59 (d, *J* = 7.2 Hz, 1 H), 7.42–7.52 (m, 2 H), 7.32–7.39 (m, 1H), 6.25 (s, 1H), 3.00 (tt, *J* = 12.0, 3.4 Hz, 1H), 2.17 (dd, *J* = 13.2, 1.4 Hz, 2H), 1.83–1.91 (m, 2H), 1.51–1.72 (m, 4H), 1.35 (s, 12H), and 1.18–1.22 (m, 2H). Formula: C_18_H_28_BNO_4_S.

#### 6.7.14. 1-((3-(4,4,5,5-Tetramethyl-1,3,2-dioxaborolan-2-yl)phenyl)sulfonyl)piperidine (**37**)

Following **GP4**, compound **27** was prepared using 1-((3-bromophenyl)sulfonyl)piperidine (**23**) (650 mg, 2.14 mmol), bis(pinacolato)diboron (760 mg, 2.99 mmol), potassium acetate (420 mg, 4.28 mmol), and Pd(dppf)Cl_2_ (260 mg, 0.36 mmol) in 7 mL of dioxane. Purification: preparative HPLC (20–100% MeCN gradient). Yield: 306 mg (41%). ^1^H NMR (500 MHz, DMSO-*d*_6_) δ ppm: 1.28 (s, 12H), 1.30–1.34 (m, 2H), 1.50 (quin, *J* = 5.6 Hz, 4H), 7.63 (t, *J* = 7.6 Hz, 1H), 7.81 (ddd, *J* = 7.8, 1.9, 1.1 Hz, 1H), 7.86–7.89 (m, 1H), and 7.92 (dt, *J* = 7.2, 1.1 Hz, 1H). Formula: C_17_H_26_BNO_4_S.

### 6.8. General Procedure for the Synthesis of Compounds ***38***–***62*** (GP5)

An appropriate pinacol ester of 4-arylboronic acid (1.0 equiv.) and thiophene-based building block with urea **5** or cyclic moiety **6**–**9** (1.0 equiv.) were dissolved in anhydrous DMF. Then, K_2_CO_3_ (3.00 equiv.) was added and, under Ar, [1,1′-bis(diphenylphosphino)ferrocene]dichloropalladium(II) (Pd(dppf)Cl_2_) (0.4–0.5 equiv.) was added. The reaction was stirred overnight at 80 °C. After that time, the reaction mixture was diluted with DCM, filtered through Celite^®^, and evaporated under reduced pressure. The crude product was purified using different methods, as described below.

#### 6.8.1. *N*-(4-(2,2-Dimethyl-4-oxo-1,2,3,4-tetrahydrothieno [3,2-d]pyrimidin-6-yl)pyridin-2-yl)acetamide (**38**)

Following **GP5**, compound **38** was prepared using *N*-(4-(4,4,5,5-tetramethyl-1,3,2-dioxaborolan-2-yl)pyridin-2-yl)acetamide (**24**) (100 mg, 0.38 mmol), **6** (99 mg, 0.38 mmol), K_2_CO_3_ (158 mg, 1.14 mmol), and Pd(dppf)Cl_2_ (125 mg, 0.17 mmol) in 4 mL of DMF. Purification: column chromatography (DCM/MeOH/NH_3(aq)_ 95:5:0.05), and then the solid residue was washed with MeCN. Yield: 39 mg (33%), yellow solid, mp 284–285 °C. Purity: 97% (UPLC/MS). ^1^H NMR (500 MHz, DMSO-*d*_6_) δ ppm: 1.42 (s, 6H), 2.11 (s, 3H), 7.05 (d, *J* = 2.6 Hz, 1H), 7.13 (s, 1H), 7.35 (dd, *J* = 5.2, 2.0 Hz, 1H), 7.66 (s, 1H), 8.30–8.37 (m, 2H), and 10.62 (s, 1H). ^13^C NMR (126 MHz, DMSO-*d*_6_) δ ppm: 23.99, 28.23 (2C), 69.10, 105.91, 108.61, 115.30, 116.00, 141.82, 145.13, 148.92, 151.70, 153.05, 160.73, and 169.67. Formula: C_15_H_16_N_4_O_2_S. MW: 316.38. MS: *m/z* 317 (M + H^+^).

#### 6.8.2. *N*-(4-(2,2-Dimethyl-4-oxo-1,2,3,4-tetrahydrothieno [3,2-d]pyrimidin-6-yl)pyridin-2-yl)isobutyramide (**39**)

Following **GP5**, compound **39** was prepared using *N*-(4-(4,4,5,5-tetramethyl-1,3,2-dioxaborolan-2-yl)pyridin-2-yl)isobutyramide (**25**) (100 mg, 0.34 mmol), **6** (90 mg, 0.34 mmol), K_2_CO_3_ (141 mg, 1.02 mmol), Pd(dppf)Cl_2_ (112 mg, 0.15 mmol), and DMF (3.5 mL). Purification: column chromatography (5% MeOH in DCM). Yield: 44 mg (38%), yellow solid, mp 226–227 °C. Purity: 100% (UPLC/MS). ^1^H NMR (500 MHz, DMSO-*d*_6_) δ ppm: 10.53 (s, 1 H), 8.34 (d, *J* = 1.1 Hz, 1 H), 8.29 (d, *J* = 5.7 Hz, 1 H), 7.63 (s, 1 H), 7.32 (dd, *J* = 5.3, 1.9 Hz, 1 H), 7.08 (d, *J* = 0.9 Hz, 1 H), 7.02 (s, 1 H), 2.73 (quin, *J* = 6.8 Hz, 1 H), and 1.38 (s, 6 H), 1.06 (d, *J* = 6.9 Hz, 6 H). ^13^C NMR (DMSO-d_6_, 126 MHz) δ ppm: 176.6, 160.7, 153.2, 151.7, 148.9, 145.2, 141.8, 116.0, 115.3, 108.7, 105.9, 69.1, 34.5, 28.2 (2C), and 19.4 (2C). Formula: C_17_H_20_N_4_O_2_S. MW: 344.43. MS: *m/z* 344 (M + H^+^).

#### 6.8.3. *N*-(4-(2,2-Dimethyl-4-oxo-1,2,3,4-tetrahydrothieno [3,2-d]pyrimidin-6-yl)pyridin-2-yl)-cyclopropanecarboxamide (**40**)

Following **GP5**, compound **40** was prepared using *N*-(4-(4,4,5,5-tetramethyl-1,3,2-dioxaborolan-2-yl)pyridin-2-yl)cyclopropanecarboxamide (**26**) (110 mg, 0.38 mmol), **6** (100 mg, 0.38 mmol), K_2_CO_3_ (158 mg, 1.14 mmol), and Pd(dppf)Cl_2_ (125 mg, 0.17 mmol) in 4 mL of DMF. Purification: flash chromatography (DCM/MeOH/NH_3(aq)_ 9:1:0.1). Yield: 54 mg (41%), brown solid, mp 257–258 °C. Purity: 95% (UPLC/MS). ^1^H NMR (500 MHz, METHANOL-*d*_4_) δ ppm: 0.89–0.94 (m, 2H), 0.99–1.03 (m, 2H), 1.54 (s, 6H), 1.86–1.96 (m, 1H), 3.35 (s, 1H), 7.04 (s, 1H), 7.31 (dd, *J* = 5.2, 1.1 Hz, 1H), 7.59 (br s, 2H), 8.30 (d, *J* = 5.2 Hz, 1H), and 8.38 (s, 1H). ^13^C NMR (126 MHz, DMSO-*d*_6_) δ ppm: 7.85 (2C), 14.27, 28.21 (2C), 69.14, 105.96, 108.66, 115.25, 116.07, 141.90, 145.20, 148.97, 151.74, 153.05, 160.77, and 173.05. Formula: C_17_H_18_N_4_O_2_S. MW: 342.42. MS: *m/z* 343 (M + H^+^).

#### 6.8.4. *N*-(4-(2,2-Dimethyl-4-oxo-1,2,3,4-tetrahydrothieno [3,2-d]pyrimidin-6-yl)pyridin-2-yl)-cyclobutanecarboxamide (**41**)

Following **GP5**, compound **41** was prepared using *N*-(4-(4,4,5,5-tetramethyl-1,3,2-dioxaborolan-2-yl)pyridin-2-yl)cyclobutanecarboxamid (**29**) (62 mg, 0.21 mmol), **6** (55 mg, 0.21 mmol), K_2_CO_3_ (87 mg, 0.63 mmol), and Pd(dppf)Cl_2_ (65 mg, 0.09 mmol) in 2.5 mL of DMF. Purification: preparative HPLC (10–60% MeCN gradient). Yield: 16 mg (22%), yellow solid, mp 252–253 °C. Purity: 98% (UPLC/MS). ^1^H NMR (500 MHz, DMSO-*d*_6_) δ ppm: 1.42 (s, 6H), 1.76–1.85 (m, 1H), 1.87–1.98 (m, 1H), 2.06–2.15 (m, 2H), 2.19–2.29 (m, 2H), 7.07 (s, 1H), 7.13 (s, 1H), 7.35 (dd, *J* = 5.4, 1.7 Hz, 1H), 7.67 (s, 1H), 8.30–8.34 (m, 1H), 8.39 (s, 1H), and 10.45 (s, 1H). ^13^C NMR (126 MHz, DMSO-*d*_6_) δ ppm: 17.70, 24.46 (2C), 28.24 (2C), 40.10, 69.12, 105.95, 108.75, 115.26, 116.06, 141.84, 145.18, 148.92, 151.72, 153.14, 160.75, and 174.12. Formula: C_18_H_20_N_4_O_2_S. MW: 356.44. MS: *m/z* 257 (M + H^+^).

#### 6.8.5. *N*-(4-(2,2-Dimethyl-4-oxo-1,2,3,4-tetrahydrothieno [3,2-d]pyrimidin-6-yl)pyridin-2-yl)-cyclohexanecarboxamide (**42**)

Following **GP5**, compound **42** was prepared using *N*-(4-(4,4,5,5-tetramethyl-1,3,2-dioxaborolan-2-yl)pyridin-2-yl)cyclohexanecarboxamide (**30**) (112 mg, 0.34 mmol), **6** (90 mg, 0.34 mmol), K_2_CO_3_ (141 mg, 1.02 mmol), and Pd(dppf)Cl_2_ (110 mg, 0.15 mmol) in 3.5 mL of DMF. Purification: column chromatography (DCM/MeOH 97:3), and then the solid residue was washed with MeCN. Yield: 19 mg (15%), yellow solid, mp 268–269 °C. Purity: 100% (UPLC/MS). ^1^H NMR (500 MHz, DMSO-*d*_6_) δ ppm: 1.12–1.32 (m, 4H), 1.39 (br s, 1H), 1.40–1.44 (m, 7H), 1.64 (br d, *J* = 11.5 Hz, 1H), 1.74 (br d, *J* = 12.3 Hz, 2H), 1.80 (br d, *J* = 12.3 Hz, 2H), 7.06 (s, 1H), 7.11 (s, 1H), 7.35 (dd, *J* = 5.2, 1.7 Hz, 1H), 7.66 (s, 1H), 8.32 (d, *J* = 5.2 Hz, 1H), 8.36 (s, 1H), and 10.50 (s, 1H). ^13^C NMR (126 MHz, DMSO-*d*_6_) δ ppm: 25.17 (2C), 25.39, 28.24 (2C), 29.02 (2C), 44.29, 69.10, 105.91, 108.72, 115.22, 116.03, 141.79, 145.17, 148.90, 151.70, 153.21, 160.72, and 175.64. Formula: C_20_H_24_N_4_O_2_S. MW: 384.50. MS: *m/z* 385 (M + H^+^).

#### 6.8.6. *N*-(4-(2,2-Dimethyl-4-oxo-1,2,3,4-tetrahydrothieno [3,2-d]pyrimidin-6-yl)pyridin-2-yl)benzamide (**43**)

Following **GP5**, compound **43** was prepared using *N*-(4-(4,4,5,5-tetramethyl-1,3,2-dioxaborolan-2-yl)pyridin-2-yl)benzamide (**31**) (94 mg, 0.29 mmol), **6** (75 mg, 0.29 mmol), K_2_CO_3_ (120 mg, 0.87 mmol), and Pd(dppf)Cl_2_ (95 mg, 0.13 mmol) in 3 mL of DMF. Purification: column chromatography (DCM/MeOH 98:2), and then the solid residue was washed with MeCN. Yield: 30 mg (28%), yellow solid, mp 242–243 °C. Purity: 100% (UPLC/MS). ^1^H NMR (500 MHz, DMSO-*d*_6_) δ ppm: 1.43 (s, 6H), 7.12 (s, 1H), 7.15 (s, 1H), 7.45 (dd, *J* = 5.3, 1.6 Hz, 1H), 7.51–7.55 (m, 2H), 7.59–7.63 (m, 1H), 7.68 (s, 1H), 8.04 (d, *J* = 1.4 Hz, 1H), 8.05 (s, 1H), 8.43 (d, *J* = 5.2 Hz, 1H), 8.47 (d, *J* = 1.1 Hz, 1H), and 10.95 (s, 1H). ^13^C NMR (126 MHz, DMSO-*d*_6_) δ ppm: 28.25 (2C), 69.13, 106.04, 110.03, 115.91, 116.19, 128.08 (2C), 128.41 (2C), 132.08, 133.96, 141.90, 145.06, 148.96, 151.75, 153.17, 160.74, and 166.33. Formula: C_20_H_18_N_4_O_2_S. MW: 378.45. MS: *m/z* 379 (M + H^+^).

#### 6.8.7. *N*-(4-(2,2-Dimethyl-4-oxo-1,2,3,4-tetrahydrothieno [3,2-d]pyrimidin-6-yl)pyridin-2-yl)-methanesulfonamide (**44**)

Following **GP5**, compound **44** was prepared using *N*-(4-(4,4,5,5-tetramethyl-1,3,2-dioxaborolan-2-yl)pyridin-2-yl)methanesulfonamide (**32**) (146 mg, 0.49 mmol), **6** (127 mg, 0.49 mmol), K_2_CO_3_ (203 mg, 1.47 mmol), and Pd(dppf)Cl_2_ (160 mg, 0.22 mmol) in 5 mL of DMF. Purification: column chromatography (DCM/MeOH/NH_3(aq)_ 9:1:0.01), and then the solid residue was washed with MeCN. Yield: 12 mg (7%), dark yellow solid, mp 303–304 °C. Purity: 100% (UPLC/MS). ^1^H NMR (500 MHz, DMSO-*d*_6_) δ ppm: 1.42 (s, 6H), 3.30 (s, 3H), 7.05 (s, 1H), 7.14 (br s, 1H), 7.16 (s, 1H), 7.27 (br s, 1H), 7.50 (br s, 1H), 7.69 (s, 1H), and 8.26 (br s, 1H). Due to the low solubility of the compound, no signals were detected in the ^13^C NMR. Formula: C_14_H_16_N_4_O_3_S_2_. MW: 352.43. MS: *m/z* 353 (M + H^+^).

#### 6.8.8. *N*-(4-(2,2-Dimethyl-4-oxo-1,2,3,4-tetrahydrothieno [3,2-d]pyrimidin-6-yl)pyridin-2-yl)-ethanesulfonamide (**45**)

Following **GP5**, compound **45** was prepared using *N*-(4-(4,4,5,5-tetramethyl-1,3,2-dioxaborolan-2-yl)pyridin-2-yl)ethanesulfonamide (**33**) (106 mg, 0.34 mmol), **6** (90 mg, 0.34 mmol), K_2_CO_3_ (141 mg, 1.02 mmol), and Pd(dppf)Cl_2_ (115 mg, 0.16 mmol) in 3.5 mL of DMF. Purification: preparative HPLC (10–60% MeCN gradient). Yield: 14 mg (11%), yellow solid, mp 284–285 °C. Purity: 96% (UPLC/MS). ^1^H NMR (500 MHz, DMSO-*d*_6_) δ ppm: 1.23 (t, *J* = 7.3 Hz, 3H), 1.42 (s, 6H), 3.45 (br d, *J* = 7.4 Hz, 2H), 7.05 (s, 1H), 7.16 (s, 2H), 7.26 (br d, *J* = 4.0 Hz, 1H), 7.68 (s, 1H), 8.24 (br d, *J* = 2.3 Hz, 1H), and 10.79 (br s, 1H). ^13^C NMR (126 MHz, DMSO-*d*_6_) δ ppm: 8.06, 28.23 (2C), 47.33, 69.12, 106.22, 107.56, 107.59, 116.42, 142.52, 144.34, 151.68, 153.33, 153.41, and 160.69. Formula: C_15_H_18_N_4_O_3_S_2_. MW: 366.45. MS: *m/z* 367 (M + H^+^).

#### 6.8.9. 6-(2-((Cyclopropylmethyl)amino)pyridin-4-yl)-2,2-dimethyl-2,3-dihydrothieno [3,2-d]pyrimidin-4(1H)-one (**46**)

Following **GP5**, compound **46** was prepared using *N*-(cyclopropylmethyl)-4-(4,4,5,5-tetramethyl-1,3,2-dioxaborolan-2-yl)pyridin-2-amine (**35**) (93 mg, 0.34 mmol), **6** (90 mg, 0.34 mmol), K_2_CO_3_ (141 mg, 1.02 mmol), and Pd(dppf)Cl_2_ (110 mg, 0.15 mmol) in 3 mL of DMF. Purification: flash chromatography (DCM/MeOH 95:5). Yield: 45 mg (40%), yellow solid, mp 232–233 °C. Purity: 95% (UPLC/MS). ^1^H NMR (DMSO-d_6_, 500 MHz) δ ppm: 7.97 (d, 1H, *J* = 5.4 Hz), 7.59 (s, 1H), 7.08 (s, 1H), 6.93 (s, 1H), 6.77 (t, 1H, *J* = 5.6 Hz), 6.6–6.7 (m, 2H), 3.14 (t, 2H, *J* = 6.2 Hz), 1.41 (s, 6H), 1.0–1.1 (m, 1H), 0.4–0.5 (m, 2H), and 0.2–0.2 (m, 2H). ^13^C NMR (DMSO-d_6_, 126 MHz) δ ppm: 160.8, 159.5, 151.6, 148.6, 146.4, 140.4, 115.1, 107.8, 104.9, 103.6, 69.0, 45.2, 28.2 (2C), 10.9, and 3.4 (2C). Formula: C_17_H_20_N_24_OS. MW: 328.43. MS: *m/z* 329 (M + H^+^).

#### 6.8.10. 5-(2-Acetamidopyridin-4-yl)-3-ureidothiophene-2-carboxamide (**47**)

Following **GP5**, compound **47** was prepared using *N*-(4-(4,4,5,5-tetramethyl-1,3,2-dioxaborolan-2-yl)pyridin-2-yl)acetamide (**24**) (81 mg, 0.31 mmol), **5** (82 mg, 0.31 mmol), K_2_CO_3_ (129 mg, 0.93 mmol), and Pd(dppf)Cl_2_ (102 mg, 0.14 mmol) in 3 mL of DMF. Purification: column chromatography (10% MeOH in DCM). Yield: 8 mg (8%), brownish solid, mp degradation over 365 °C. ^1^H NMR (500 MHz, DMSO-*d*_6_) δ ppm: 10.61 (s, 1H), 10.00 (s, 1H), 8.38 (s, 1H), 8.27–8.36 (m, 2H), 7.41–7.70 (m, 2H), 7.28 (dd, *J* = 5.2, 1.4 Hz, 1H), 6.65 (br s, 2H), and 2.08 (s, 3H). ^13^C NMR (DMSO-d_6_, 126 MHz) δ ppm: 170.2, 165.7, 155.4, 153.6, 149.6, 145.7, 142.2, 141.1, 120.7, 115.7, 110.8, 109.1, and 24.5. Formula: C_13_H_13_N_5_O_3_S. MW: 319.34. MS: *m/z* 320 (M + H^+^).

#### 6.8.11. 5-(2-Isobutyramidopyridin-4-yl)-3-ureidothiophene-2-carboxamide (**48**)

Following **GP5**, compound **48** was prepared using *N*-(4-(4,4,5,5-tetramethyl-1,3,2-dioxaborolan-2-yl)pyridin-2-yl)isobutyramide (**25**) (92 mg, 0.32 mmol), **5** (85 mg, 0.32 mmol), K_2_CO_3_ (133 mg, 0.96 mmol), and Pd(dppf)Cl_2_ (100 mg, 0.14 mmol) in 3 mL of DMF. Purification: preparative HPLC (5–50% MeCN gradient). Yield: 7 mg (6%), yellow solid, mp degradation over 308 °C. Purity: 96% (UPLC/MS). ^1^H NMR (500 MHz, DMSO-*d*_6_) δ ppm: 1.10 (d, *J* = 6.9 Hz, 6H), 2.72–2.83 (m, 1H), 6.69 (br s, 1H), 7.33 (dd, *J* = 5.2, 1.7 Hz, 1H), 7.56 (br s, 2H), 8.35 (d, *J* = 5.4 Hz, 1H), 8.42 (s, 1H), 8.45 (d, *J* = 1.1 Hz, 1H), 10.04 (s, 1H), and 10.60 (s, 1H). ^13^C NMR (126 MHz, DMSO-*d*_6_) δ ppm: 19.36 (2C), 34.50, 108.79, 110.26, 115.10, 120.13, 140.62, 141.67, 145.25, 149.06, 153.27, 154.90, 165.22, and 176.60. Formula: C_15_H_17_N_5_O_3_S. MW: 347.39. MS: *m/z* 348 (M + H^+^).

#### 6.8.12. 5-(2-(Cyclopropanecarboxamido)pyridin-4-yl)-3-ureidothiophene-2-carboxamide (**49**)

Following **GP5**, compound **49** was prepared using *N*-(4-(4,4,5,5-tetramethyl-1,3,2-dioxaborolan-2-yl)pyridin-2-yl)cyclopropanecarboxamide (**26**) (98 mg, 0.34 mmol), **5** (90 mg, 0.34 mmol), K_2_CO_3_ (141 mg, 1.02 mmol), and Pd(dppf)Cl_2_ (120 mg, 0.16 mmol) in 3.5 mL of DMF. Purification: column chromatography (DCM/MeOH/TEA 90:10:0.1). Yield: 48 mg (41%), brown solid, mp degradation over 290 °C. Purity: 100% (UPLC/MS). HRMS (ESI-QTOF): *m/z* calcd. for 346.0929; found 346.0958. ^1^H NMR (500 MHz, DMSO-*d*_6_) δ ppm: 0.76–0.94 (m, 4H), 1.97–2.08 (m, 1H), 6.69 (br s, 1H), 7.32 (s, 1H), 7.57 (br s, 3H), 8.37 (s, 1H), 8.42 (s, 2H), 10.04 (br s, 1H), and 10.97 (br s, 1H). ^13^C NMR (126 MHz, DMSO-*d*_6_) δ ppm: 7.89 (2C), 14.23, 99.49, 108.69, 110.24, 120.12, 140.60, 141.71, 145.25, 149.10, 153.11, 154.89, 165.22, and 173.03. Formula: C_15_H_15_N_5_O_3_S. MW: 345.38. MS: *m/z* 346 (M + H^+^).

#### 6.8.13. 5-(2-(Cyclobutanecarboxamido)pyridin-4-yl)-3-ureidothiophene-2-carboxamide (**50**)

Following **GP5**, compound **50** was prepared using *N*-(4-(4,4,5,5-tetramethyl-1,3,2-dioxaborolan-2-yl)pyridin-2-yl)cyclobutanecarboxamide (**29**) (60 mg, 0.20 mmol), **5** (53 mg, 0.20 mmol), K_2_CO_3_ (101 mg, 0.60 mmol), and Pd(dppf)Cl_2_ (65 mg, 0.09 mmol) in 2 mL of DMF. Purification: preparative HPLC (10–60% MeCN gradient). Yield: 16 mg (22%), cream solid, mp degradation over 346 °C. Purity: 100% (UPLC/MS). ^1^H NMR (500 MHz, DMSO-*d*_6_) δ ppm: 1.77–1.86 (m, 1H), 1.88–1.99 (m, 1H), 2.07–2.15 (m, 2H), 2.17–2.29 (m, 2H), 2.51–2.52 (m, 1H), 6.69 (br s, 1H), 7.32 (dd, *J* = 5.2, 1.7 Hz, 1H), 7.59 (br s, 1H), 8.34 (dd, *J* = 5.4, 0.6 Hz, 1H), 8.43 (s, 1H), 8.46 (s, 1H), 10.04 (s, 1H), and 10.49 (s, 1H). ^13^C NMR (126 MHz, DMSO-*d*_6_) δ ppm: 17.73, 24.45 (2C), 40.10, 108.74, 110.26, 115.07, 120.14, 140.61, 141.67, 145.24, 149.06, 153.22, 154.89, 165.22, and 174.16. Formula: C_16_H_17_N_5_O_3_S. MW: 359.40. MS: *m/z* 360 (M + H^+^).

#### 6.8.14. 5-(2-(Cyclohexanecarboxamido)pyridin-4-yl)-3-ureidothiophene-2-carboxamide (**51**)

Following **GP5**, compound **51** was prepared using *N*-(4-(4,4,5,5-tetramethyl-1,3,2-dioxaborolan-2-yl)pyridin-2-yl)cyclohexanecarboxamide (**30**) (112 mg, 0.34 mmol), **5** (90 mg, 0.34 mmol), K_2_CO_3_ (141 mg, 1.02 mmol), Pd(dppf)Cl_2_ (112 mg, 0.15 mmol), and DMF (3.5 mL). Purification: column chromatography (5% MeOH in DCM). Yield: 15 mg (11%), greyish solid, mp degradation over 315 °C. Purity: 100% (UPLC/MS). ^1^H NMR (500 MHz, DMSO-*d*_6_) δ ppm: 10.54 (s, 1 H), 10.04 (s, 1 H), 8.40–8.45 (m, 2 H), 8.35 (d, *J* = 5.4 Hz, 1 H), 7.39–7.78 (m, 2 H), 7.32 (dd, *J* = 5.2, 1.7 Hz, 1 H), 6.69 (br s, 2 H), 2.08 (s, 1 H), 1.70–1.86 (m, 4 H), 1.60–1.68 (m, 1 H), 1.34–1.47 (m, 2 H), and 1.16–1.32 (m, 3 H). ^13^C NMR (DMSO-d_6_, 126 MHz) δ ppm: 175.7, 165.2, 154.9, 153.3, 149.1, 145.2, 141.6, 140.6, 120.1, 115.1, 110.2, 108.8, 44.2, 29.0 (2C), 25.4, and 25.2 (2C). Formula: C_18_H_21_N_5_O_3_S. MW: 387.46. MS: *m/z* 388 (M + H^+^).

#### 6.8.15. 5-(2-Benzamidopyridin-4-yl)-3-ureidothiophene-2-carboxamide (**52**)

Following **GP5**, compound **52** was prepared using *N*-(4-(4,4,5,5-tetramethyl-1,3,2-dioxaborolan-2-yl)pyridin-2-yl)benzamide (**31**) (100 mg, 0.31 mmol), **5** (82 mg, 0.31 mmol), K_2_CO_3_ (129 mg, 0.93 mmol), Pd(dppf)Cl_2_ (102 mg, 0.14 mmol), and DMF (3 mL). Purification: column chromatography (5% MeOH in DCM). Yield: 13 mg (11%), brown solid, mp degradation over 300 °C. Purity: 100% (UPLC/MS). ^1^H NMR (500 MHz, DMSO-*d*_6_) δ ppm: 10.97 (s, 1 H), 10.06 (s, 1 H), 8.55 (d, *J* = 1.1 Hz, 1 H), 8.42–8.51 (m, 2 H), 8.02–8.10 (m, 2 H), 7.58–7.65 (m, 2 H), 7.49–7.57 (m, 3 H), 7.42 (dd, *J* = 5.3, 1.6 Hz, 1 H), and 6.64–6.80 (m, 1 H). ^13^C NMR (DMSO-d_6_, 126 MHz) δ ppm:166.8, 165.7, 155.4, 153.8, 149.6, 145.8, 142.3, 141.0, 134.4, 132.6, 128.9 (2C), 128.6 (2C), 120.8, 116.3, 110.9, and 110.4. Formula: C_18_H_15_N_5_O_3_S. MW: 381.41. MS: *m/z* 382 (M + H^+^).

#### 6.8.16. N-(3-(2,2-Dimethyl-4-oxo-1,2,3,4-tetrahydrothieno [3,2-d]pyrimidin-6-yl)phenyl)cyclopropanecarboxamide (**53**)

Following **GP5**, compound **53** was prepared using *N*-(3-(4,4,5,5-tetramethyl-1,3,2-dioxaborolan-2-yl)phenyl)cyclopropanecarboxamide (**27**) (98 mg, 0.34 mmol), **6** (90 mg, 0.34 mmol), K_2_CO_3_ (141 mg, 1.02 mmol), Pd(dppf)Cl_2_ (112 mg, 0.15 mmol), and DMF (3.5 mL). Purification: column chromatography (5–10% MeOH in DCM). Yield: 38 mg (33%), brown–green solid, mp 157–158 °C. Purity: 100% (UPLC/MS). ^1^H NMR (500 MHz, DMSO-*d*_6_) δ ppm: 10.30 (s, 1 H), 7.88 (t, *J* = 1.6 Hz, 1 H), 7.50–7.56 (m, 1 H), 7.48 (s, 1 H), 7.24–7.34 (m, 2 H), 7.01 (d, *J* = 0.9 Hz, 1 H), 6.78 (s, 1 H), 1.70–1.79 (m, 1 H), 1.38 (s, 6 H), AND 0.74–0.81 (m, 4 H). ^13^C NMR (DMSO-d_6_, 126 MHz) δ ppm: 172.4, 161.5, 152.4, 148.8, 140.6, 134.1, 130.2, 120.6, 119.7, 116.2, 114.3, 104.6, 69.5, 28.7 (2C), 15.2, and 7.9 (2C). Formula: C_18_H_19_N_3_O_2_S. MW: 341.43. MS: *m/z* 342 (M + H^+^).

#### 6.8.17. *N*-(5-(2,2-Dimethyl-4-oxo-1,2,3,4-tetrahydrothieno [3,2-d]pyrimidin-6-yl)-2-fluorophenyl)- cyclopropanecarboxamide (**54**)

Following **GP5**, compound **54** was prepared using *N*-(2-fluoro-5-(4,4,5,5-tetramethyl-1,3,2-dioxaborolan-2-yl)phenyl)cyclopropanecarboxamide (**28**) (70 mg, 0.23 mmol), **6** (60 mg, 0.23 mmol), K_2_CO_3_ (95 mg, 0.69 mmol), and Pd(dppf)Cl_2_ (80 mg, 0.11 mmol) in 3 mL of DMF. Purification: flash chromatography (DCM/MeOH/NH_3(aq)_ 9.5:0.5:0.05), and then the solid residue was crystallized from MeCN. Yield: 31 mg (37%), beige solid, mp 199–200 °C. Purity: 95% (UPLC/MS). ^1^H NMR (500 MHz, ACETONE-*d*_6_) δ ppm: 0.81–0.86 (m, 2H), 0.92–0.97 (m, 2H), 1.57 (s, 6H), 2.00–2.04 (m, 1H), 6.29 (br s, 1H), 6.66 (br s, 1H), 6.85 (s, 1H), 7.22 (dd, *J* = 10.9, 8.6 Hz, 1H), 7.35 (ddd, *J* = 8.5, 4.7, 2.3 Hz, 1H), 8.66 (dd, *J* = 7.3, 1.9 Hz, 1H), and 9.32 (br s, 1H). ^13^C NMR (126 MHz, ACETONE-*d*_6_) δ ppm: 7.66 (2C), 14.74, 28.28 (2C), 69.81, 106.20, 114.47, 115.96 (d, *J* = 20.5 Hz), 120.03, 121.56 (br d, *J* = 7.2 Hz) 128.17 (d, *J* = 11.5 Hz) 130.66 (d, *J* = 3.6 Hz) 148.57, 153.10 (d, *J* = 252.3 Hz) 152.22, 161.30, and 172.76. Formula: C_18_H_18_FN_3_O_2_S. MW: 359.42. MS: *m/z* 360 (M + H^+^).

#### 6.8.18. *N*-(3-(2,2-Dimethyl-4-oxo-1,2,3,4-tetrahydrothieno [3,2-d]pyrimidin-6-yl)phenyl)ethanesulfonamide (**55**)

Following **GP5**, compound **55** was prepared using *N*-(3-(4,4,5,5-tetramethyl-1,3,2-dioxaborolan-2-yl)phenyl)ethanesulfonamide (**34**) (106 mg, 0.34 mmol), **6** (90 mg, 0.34 mmol), K_2_CO_3_ (141 mg, 1.02 mmol), and Pd(dppf)Cl_2_ (115 mg, 0.16 mmol) in 3.5 mL of DMF. Purification: preparative HPLC (10–60% MeCN gradient). Yield: 78 mg (62%), brown solid, mp 119–200 °C. Purity: 96% (UPLC/MS). ^1^H NMR (500 MHz, DMSO-*d*_6_) δ ppm: 1.20 (t, *J* = 7.3 Hz, 3H), 1.41 (s, 6H), 3.12–3.17 (m, 2H), 6.83 (s, 1H), 7.07 (s, 1H), 7.22 (dt, *J* = 7.7, 1.8 Hz, 1H), 7.34–7.40 (m, 2H), 7.44–7.46 (m, 1H), 7.54 (s, 1H), and 9.96 (br s, 1H). ^13^C NMR (126 MHz, DMSO-*d*_6_) δ ppm: 8.05, 28.20 (2C), 45.27, 69.00, 104.29, 114.13, 115.77, 119.28, 120.73, 130.29, 134.20, 139.26, 147.68, 151.87, and 160.94. Formula: C_16_H_19_N_3_O_3_S_2_. MW: 365.47. MS: *m/z* 366 (M + H^+^).

#### 6.8.19. *N*-(3-(2,2-Dimethyl-4-oxo-1,2,3,4-tetrahydrothieno [3,2-d]pyrimidin-6-yl)phenyl)cyclohexanesulfonamide (**56**)

Following **GP5**, compound **56** was prepared using *N*-(3-(4,4,5,5-tetramethyl-1,3,2-dioxaborolan-2-yl)phenyl)cyclohexanesulfonamide (**36**) (84 mg, 0.23 mmol), **6** (60 mg, 0.23 mmol), K_2_CO_3_ (95 mg, 0.69 mmol), Pd(dppf)Cl_2_ (76 mg, 0.10 mmol), and DMF (2 mL). Purification: column chromatography (2% MeOH in DCM). Yield: 28 mg (29%), yellow solid, mp 275–276 °C. Purity: 100% (UPLC/MS). ^1^H NMR (500 MHz, DMSO-*d*_6_) δ ppm: 9.90 (br s, 1 H), 7.50 (s, 1 H), 7.41–7.44 (m, 1 H), 7.27–7.35 (m, 2 H), 7.16–7.20 (m, 1 H), 7.04 (d, *J* = 0.9 Hz, 1 H), 6.79 (s, 1 H), 2.99 (tt, *J* = 11.8, 3.4 Hz, 1 H), 1.95–2.03 (m, 2 H), 1.65–1.77 (m, 2 H), 1.47–1.57 (m, 1 H), 1.33–1.42 (m, 8 H), 1.12–1.23 (m, 2 H), and 1.03–1.11 (m, 1 H). ^13^C NMR (DMSO-d_6_, 126 MHz) δ ppm: 160.9, 151.9, 147.7, 139.5, 134.1, 130.2, 120.5, 119.1, 115.5, 114.1, 104.2, 69.0, 59.3, 28.2 (2C), 26.0, 24.7, and 24.3 (2C). Formula: C_20_H_25_N_3_O_3_S_2_. MW: 419.56. MS: *m/z* 420 (M + H^+^).

#### 6.8.20. 2,2-Dimethyl-6-(3-(piperidin-1-ylsulfonyl)phenyl)-2,3-dihydrothieno [3,2-d]pyrimidin-4(1H)-one (**57**)

Following **GP5**, compound **57** was prepared using 1-((3-(4,4,5,5-tetramethyl-1,3,2-dioxaborolan-2-yl)phenyl)sulfonyl)piperidine (**37**) (97 mg, 0.28 mmol), **6** (73 mg, 0.28 mmol), K_2_CO_3_ (116 mg, 0.84 mmol), and Pd(dppf)Cl_2_ (95 mg, 0.13 mmol) in 3 mL of DMF. Purification: column chromatography (DCM/MeOH 97:3), and then the solid residue was washed with MeCN. Yield: 24 mg (21%), beige solid, mp 202–203 °C. Purity: 97% (UPLC/MS). ^1^H NMR (500 MHz, DMSO-*d*_6_) δ ppm: 1.34–1.39 (m, 2H), 1.42 (s, 6H), 1.51–1.57 (m, 4H), 2.92 (br t, *J* = 5.3 Hz, 4H), 7.04 (s, 1H), 7.10 (s, 1H), 7.61 (s, 1H), 7.68–7.73 (m, 2H), 7.83 (d, *J* = 0.9 Hz, 1H), and 7.98 (ddd, *J* = 5.4, 3.4, 2.0 Hz, 1H). ^13^C NMR (126 MHz, DMSO-*d*_6_) δ ppm: 22.79, 24.70 (2C), 28.20 (2C), 46.62 (2C), 69.08, 105.17, 115.47, 123.54, 127.34, 129.95, 130.53, 134.27, 136.62, 145.91, 151.92, and 160.83. Formula: C_19_H_23_N_3_O_3_S_2_. MW: 405.53. MS: *m/z* 406 (M + H^+^).

#### 6.8.21. 5-(3-(Cyclopropanecarboxamido)phenyl)-3-ureidothiophene-2-carboxamide (**58**)

Following **GP5**, compound **58** was prepared using *N*-(3-(4,4,5,5-tetramethyl-1,3,2-dioxaborolan-2-yl)phenyl)cyclopropanecarboxamide (**27**) (98 mg, 0.34 mmol), **5** (90 mg, 0.34 mmol), K_2_CO_3_ (141 mg, 1.02 mmol), and Pd(dppf)Cl_2_ (110 mg, 0.15 mmol) in 3.5 mL of DMF. Purification: column chromatography (DCM/MeOH 9:1), and then the solid residue was washed with MeCN. Yield: 24 mg (21%), beige solid, mp 240–241 °C. Purity: 100% (UPLC/MS). ^1^H NMR (500 MHz, DMSO-*d*_6_) δ ppm: 0.79–0.84 (m, 4H), 1.75–1.82 (m, 1H), 6.65 (br s, 2H), 7.26–7.29 (m, 1H), 7.38 (t, *J* = 7.9 Hz, 1H), 7.45 (br d, *J* = 3.4 Hz, 1H), 7.56 (dd, *J* = 8.2, 1.0 Hz, 1H), 8.05 (s, 1H), 8.23 (s, 1H), 10.07 (s, 1H), and 10.39 (s, 1H). ^13^C NMR (126 MHz, DMSO-*d*_6_) δ ppm: 7.36 (2C), 14.64, 108.40, 115.60, 118.03, 119.09, 119.87, 129.81, 133.37, 140.22, 143.64, 145.37, 154.93, 165.53, and 171.96. Formula: C_16_H_16_N_4_O_3_S. MW: 344.39. MS: *m/z* 345 (M + H^+^).

#### 6.8.22. 5-(3-(Cyclopropanecarboxamido)-4-fluorophenyl)-3-ureidothiophene-2-carboxamide (**59**)

Following **GP5**, compound **59** was prepared using *N*-(2-fluoro-5-(4,4,5,5-tetramethyl-1,3,2-dioxaborolan-2-yl)phenyl)cyclopropanecarboxamide (**28**) (80 mg, 0.26 mmol), **5** (68 mg, 0.26 mmol), K_2_CO_3_ (108 mg, 0.78 mmol), and Pd(dppf)Cl_2_ (95 mg, 0.13 mmol) in 3 mL of DMF. Purification: column chromatography (DCM/MeOH/NH_3(aq)_ 9.5:0.5:0.05), and then the solid residue was washed with MeCN. Yield: 50 mg (53%), brown solid, mp degradation over 305 °C. Purity: 95% (UPLC/MS). ^1^H NMR (500 MHz, DMSO-*d*_6_) δ ppm: 0.72–0.87 (m, 4H), 1.95–2.08 (m, 1H), 6.61 (br s, 1H), 7.31 (br s, 2H), 7.47 (br s, 3H), 8.13 (s, 1H), 8.32 (br d, *J* = 6.3 Hz, 1H), 10.02 (s, 1H), and 10.11 (s, 1H). ^13^C NMR (126 MHz, DMSO-*d*_6_) δ ppm: 7.69 (2C), 14.08, 108.46, 116.40 (d, *J* = 20.5 Hz), 118.18, 120.19, 121.54 (d, *J* = 6.6 Hz), 127.24 (d, *J* = 12.1 Hz), 129.39 (d, *J* = 6.0 Hz), 142.65, 145.39, 153.03 (d, *J* = 238.4 Hz), 154.90, 165.46, and 172.52. Formula: C_16_H_15_FN_4_O_3_S. MW: 362.38 MS: *m/z* 363 (M + H^+^).

#### 6.8.23. *N*-(4-(4’-Oxo-3’,4’-dihydro-1’H-spiro[cyclopentane-1,2’-thieno [3,2-d]pyrimidin]-6’-yl)pyridin-2-yl)cyclopropanecarboxamide (**60**)

Following **GP5**, compound **60** was prepared using *N*-(4-(4,4,5,5-tetramethyl-1,3,2-dioxaborolan-2-yl)pyridin-2-yl)cyclopropanecarboxamide (**26**) (92 mg, 0.32 mmol), **7** (92 mg, 0.32 mmol), K_2_CO_3_ (133 mg, 0.96 mmol), and Pd(dppf)Cl_2_ (55 mg, 0.08 mmol) in 3 mL of DMF. Purification: preparative HPLC (10–60% MeCN gradient). Yield: 60 mg (51%), yellow solid, mp 225–226 °C. Purity: 99% (UPLC/MS). ^1^H NMR (500 MHz, DMSO-*d*_6_) δ ppm: 0.79–0.89 (m, 4H), 1.60–1.73 (m, 4H), 1.78–1.91 (m, 4H), 1.98–2.06 (m, 1H), 7.07 (s, 1H), 7.22 (s, 1H), 7.34 (dd, *J* = 5.3, 1.6 Hz, 1H), 7.82 (s, 1H), 8.31–8.37 (m, 2H), and 10.93 (s, 1H). ^13^C NMR (126 MHz, DMSO-*d*_6_) δ ppm: 7.83 (2C), 14.27, 21.87 (2C), 38.65 (2C), 79.01, 106.99, 108.73, 115.20, 116.11, 141.86, 145.11, 148.94, 152.23, 153.02, 161.14, and 173.01. Formula: C_19_H_20_N_4_O_2_S. MW: 368.46. MS: *m/z* 369 (M + H^+^).

#### 6.8.24. *N*-(4-(4′-Oxo-3′,4,4′,5-tetrahydro-1′H,3H-spiro[furan-2,2′-thieno [3,2-d]pyrimidin]-6′-yl)pyridin-2-yl)cyclopropanecarboxamide (**61**)

Following **GP5**, compound **61** was prepared using *N*-(4-(4,4,5,5-tetramethyl-1,3,2-dioxaborolan-2-yl)pyridin-2-yl)cyclopropanecarboxamide (**26**) (104 mg, 0.36 mmol), **8** (102 mg, 0.36 mmol), K_2_CO_3_ (149 mg, 1.08 mmol), and Pd(dppf)Cl_2_ (60 mg, 0.08 mmol) in 3.5 mL of DMF. Purification: flash chromatography (DCM/MeOH/NH_3(aq)_ 9:1:0.01). Yield: 12 mg (9%), white solid, mp 314–315 °C. Purity: 100% (UPLC/MS). ^1^H NMR (500 MHz, DMSO-*d*_6_) δ ppm: 0.81–0.91 (m, 4H), 1.83–1.91 (m, 2H), 2.01–2.07 (m, 1H), 2.69 (t, *J* = 7.4 Hz, 2H), 3.43–3.50 (m, 2H), 4.56 (t, *J* = 4.9 Hz, 1H), 7.59 (dd, *J* = 5.2, 1.7 Hz, 1H), 7.95 (s, 1H), 8.41 (dd, *J* = 5.3, 0.7 Hz, 1H), 8.47 (d, *J* = 1.1 Hz, 1H), 11.01 (s, 1H), and 12.51 (s, 1H). ^13^C NMR (126 MHz, DMSO-*d*_6_) δ ppm: 7.87 (2C), 14.28, 29.97, 30.91, 59.97, 109.32, 115.75, 121.08, 123.60, 141.38, 147.46, 149.07, 153.14, 157.87, 158.35, 160.11, and 173.10. Formula: C_18_H_18_N_4_O_3_S. MW: 370.43. MS: *m/z* 371 (M + H^+^).

#### 6.8.25. *N*-(4-(4′-Oxo-3′,4,4′,5-tetrahydro-1′H,2H-spiro[furan-3,2′-thieno [3,2-d]pyrimidin]-6′-yl)pyridin-2-yl)cyclopropanecarboxamide (**62**)

Following **GP5**, compound **62** was prepared using *N*-(4-(4,4,5,5-tetramethyl-1,3,2-dioxaborolan-2-yl)pyridin-2-yl)cyclopropanecarboxamide (**26**) (81 mg, 0.28 mmol), **9** (81 mg, 0.28 mmol), K_2_CO_3_ (116 mg, 0.84 mmol), and Pd(dppf)Cl_2_ (50 mg, 0.07 mmol) in 2.5 mL of DMF. Purification: preparative HPLC (5–50% MeCN gradient). Yield: 18 mg (18%), yellow solid, mp 249–250 °C. Purity: 98% (UPLC/MS). HRMS (ESI-QTOF): *m/z* calcd. for 371.1133; found 371.1161. ^1^H NMR (500 MHz, DMSO-*d*_6_) δ ppm: 0.78–0.89 (m, 4H), 1.99–2.07 (m, 1H), 2.10–2.24 (m, 2H), 3.60 (d, *J* = 8.9 Hz, 1H), 3.76 (d, *J* = 8.9 Hz, 1H), 3.80–3.95 (m, 2H), 7.11 (s, 1H), 7.36 (dd, *J* = 5.2, 1.7 Hz, 1H), 7.59 (s, 1H), 8.07 (s, 1H), 8.31–8.39 (m, 2H), and 10.95 (s, 1H). ^13^C NMR (126 MHz, DMSO-*d*_6_) δ ppm: 7.85 (2C), 14.28, 38.77, 65.98, 76.48, 77.68, 107.07, 108.79, 115.28, 116.06, 141.73, 145.71, 148.99, 152.08, 153.03, 160.94, and 173.04. Formula: C_18_H_18_N_4_O_3_S. MW: 370.43. MS: *m/z* 371 (M + H^+^).

### 6.9. Evaluation of In Vitro Inhibitory Potencies towards Kinases GSK-3β, IKK-β, and ROCK-1 

Promega’s Kinase Enzyme Systems (Promega; Madison, WI, USA) were used to determine the activities of the tested compounds against the human recombinant kinases GSK-3β, IKK- β, and ROCK-1. The assays were performed according to the provided manufacturer’s instruction, based on the optimized conditions of the enzymatic reactions, using the low-volume, white polystyrene, 384-well plates. The ADP-Glo™ Assay (Promega; Madison, WI, USA) was used for bioluminescent detection of the kinase activity. Stock solutions of the tested compounds in DMSO (1mM) were diluted with the Kinase Assay Buffer before use (40 mM Tris, pH 7.5, enriched with 50 µM dithiothreitol; DTT), giving the final desired concentrations. The kinase enzymes, substrates, and ATP solutions were also diluted with the assay buffer prior to use. The GSK-3β, IKK-β, and ROCK-1 substrates were derived from human muscle glycogen synthase 1, human IkBA, and human 40S ribosomal protein S6, respectively. The assay procedure was as follows: At first, the appropriate kinase enzyme (GSK-3β: 10 ng per well; IKK-β: 45 ng per well; ROCK-1: 24 ng per well) was incubated with the tested sample (10 µM in well) for 5 min. The DMSO solution (1% in well) was used in the blank samples instead of the tested inhibitors. After the incubation period, ATP (25 µM per well in the case of GSK-3β and IKK-β kinases; 5 µM per well in the case of ROCK-1 kinase) and an appropriate enzyme substrate (0.2 µg/µL in well) were added to start the enzymatic reaction. The reaction mixture was kept at room temperature for 1 h; then, the ADP-Glo™ reagent was added, to terminate the kinase reaction and deplete any remaining ATP. After 40 min, a second reagent (Kinase Detection Reagent) was applied to convert the obtained ADP to ATP and to generate light from the newly synthesized ATP using a luciferase/luciferin reaction. The mixture was incubated for another 30 min at room temperature before the luminescence was measured. The calculations of the percent of kinases’ inhibition by tested compounds were based on the equation 100 − (S/B) × 100; S and B are the enzyme activities with and without the tested sample, respectively. For compounds with enzyme inhibitory activities better than 50% at 10 μM, their IC_50_ values were determined based on the kinase’s inhibitory activities in the six or seven different concentrations of each compound, resulting in enzyme inhibition between 5% and 95%. IC_50_ results were obtained using nonlinear regression (GraphPad Prism 9; GraphPad Software, San Diego, CA, USA) by plotting the residual enzyme activities against the applied inhibitor concentration. Each compound was evaluated in two independent experiments, with every data point collected in duplicate. Staurosporine (Biokom, Janki, Poland) and published research compounds (**I, II**, and **III**) [50,51,52] were used as the reference compounds for enzymatic reactions.

### 6.10. Computational Analysis and Visualizations

The structural model used for the visualizations was GSK-3β co-crystallized with compound **II**; this was used as a reference in the study (PDB: 4PTC) [51]. The structure was protonated with Epik and propka and then minimized using the OPLS4 force field [69]. Missing residues were added using Prime. The deposited crystal structure was missing a methyl group in the ligand compound, thus the re-docking with induced fit procedure was employed. The RMSD value between the preprocessed 4PTC (added hydrogens and missing residues) and the structural model equaled 1.283 Å. Compounds **40** and **49** were docked to the 4PTC model prepared using the Glide SP procedure. All simulations were performed with Maestro Schrödinger (Schrödinger Release 2023-2, LLC, New York, NY, USA, 2023). Molecular graphics and analyses were performed with UCSF ChimeraX (version 1.7.1) [70,71], developed by the Resource for Biocomputing, Visualization, and Informatics at the University of California, San Francisco, with support from the National Institutes of Health R01-GM129325 and the Office of Cyber Infrastructure and Computational Biology, National Institute of Allergy and Infectious Diseases.

### 6.11. Kinetics Studies of GSK-3β Inhibition by 62

Kinetic studies were performed using the GSK-3β Kinase Enzyme System (Promega; Madison, WI, USA) and ADP-Glo™ bioluminescent assay (Promega; Madison, WI, USA), following the manufacturer’s instructions. The general workflow is described in Section 6.9. Evaluation of In Vitro Inhibitory Potencies towards Kinases GSK-3β, IKK-β, and ROCK-1. Kinetic experiments were performed by varying the concentrations of both ATP (from 10 to 100 μM) and inhibitor **62** (from 0 to 100 nM), giving enzyme inhibition of between 10% and 90%. The substrate concentration was kept constant. The luminescence was measured using the EnSpire multimode microplate reader (PerkinElmer, Waltham, MA, USA). Each data point was collected in triplicate. V_max_ and K_m_ values of the Michaelis–Menten kinetics were calculated using nonlinear regression from ATP–velocity curves. Lineweaver–Burk and Cornish–Bowden plots were obtained via linear regression in GraphPad Prism (GraphPad Prism 9; GraphPad Software, San Diego, CA, USA). The K_i_ value of inhibitor **62** was determined by replotting the Lineweaver–Burk plots data (K_m_ versus [I]).

## 7. In Cellulo Studies

### 7.1. Cells Preparation

Mouse microglial cells (BV-2) were a generous gift from Professor Bozena Kaminska-Kaczmarek of the Laboratory of Molecular Neurobiology, Neurobiology Center, Nencki Institute of Experimental Biology, Polish Academy of Sciences, Warsaw, Poland. Cells were cultured in Dulbecco’s modified Eagle’s Medium-high glucose (DMEM, Glutamx Thermo Fisher, Waltham, MA, USA) supplemented with 10% inactivated fetal bovine serum (Thermo Fisher), 100 IU/mL penicillin (Merck), and 100 µg/mL streptomycin (Merck). Cells were cultured in flasks (area 175 cm^2^, Nunc) and incubated at 37 °C, 5% CO_2_. To evaluate the level of NO, IL-6, and TNF-α and the effectiveness of the compounds tested, BV-2 microglia cells were cultured in 96-well culture plates (5 × 10^4^ cells per well, Falcon). For the measurement of cell viability and cell membrane damage, cells were placed in 96-well culture plates (2 × 10^4^ cells per well, Falcon). Before the tests, cells were grown for 24 h in the incubator (37 °C, 5% CO_2_). Mouse Hippocampal Neuronal Cell Line (HT-22) was a generous gift from Dr Bartosz Pomierny of the Department of Biochemical Toxicology, Jagiellonian University Medical College, Krakow, Poland. Cells were cultured in Dulbecco’s modified Eagle’s Medium-high glucose (DMEM, Glutamx Thermo Fisher) supplemented with 10% inactivated fetal bovine serum (Thermo Fisher), 100 IU/mL penicillin (Merck), and 100 µg/mL streptomycin (Merck). Cells were cultured in flasks (area 175 cm^2^, Nunc) and incubated at 37 °C, 5% CO_2_. For the measurement of cell viability and neuroprotective effects against okadaic acid, cells were placed in 96-well culture plates (2 × 10^4^ cells per well, Falcon). Before the tests, cells were grown for 24 h in the incubator (37 °C, 5% CO_2_).

### 7.2. Preparation of Test Compound Solutions 

Stock solutions were prepared at the concentration of 10 mM for test and reference compounds. A minimum of 1 mg of each tested compound was weighed and dissolved in the appropriate volume of dimethyl sulfoxide. Serial dilutions were prepared in DMSO and then the diluted compounds were transferred to PBS. Before assays, eventual precipitation or opalescence was checked.

### 7.3. Cell Viability Assay 

Cell viability was evaluated using the PrestoBlue reagent (Thermo Fisher), according to the manufacturer’s procedures [72]. Following 24 h of incubation with the tested molecule, PrestoBlue reagent was added to a microplate well in an amount equal to one-tenth of the remaining medium volume. The resulting mixture was incubated for 15 min at 37 °C, and the fluorescence intensity (EX 530; EM 580 nm) was measured using the plate reader POLARstar Omega, (BMG Labtech, Ortenberg, Germany). The results (viability values) are provided as a percentage of live cells with respect to DMSO (control sample).

### 7.4. Okadaic Acid Treated HT-22 Cells

HT-22 cells were seeded into 96-well plates at a density of 2 × 10^4^ cells per well. Okadaic acid (Merck) was added at a concentration of 400 nM and incubated for 3 h. After this time, 10, 1, and 0.1 µM of tested compounds were added and incubated in the aseptic conditions (37 °C, 5% CO_2_). Cell viability was determined using Presto Blue assay after 24 h. All data were normalized to the percentage survival of vehicle control. Data are represented as mean SD versus vehicle control.

### 7.5. LPS-Treated BV-2 Cells

The cells were pretreated with tested compounds for 1 h. After this time, lipopolysaccharide (100 ng/mL) was added and the resulting mixture was incubated for 18 h. Next, the culture supernatant was acquired to measure the levels of nitric oxide (NO), IL-6, and TNF-α, according to the following procedures.

### 7.6. NO Release Measurement

The NO level in the culture supernatants was measured using 2,3-diaminonaphthalene (DAN) reagent, according to the method of Nussler et al. [73]. After 15 min of incubation at room temperature, the fluorescence intensity (EX 360; EM 440 nm) was measured using a microplate reader POLARstar Omega, (BMG Labtech). The values of nitric oxide were calculated as a percentage of control (maximal response of LPS).

### 7.7. Measurement of Cytokine Levels

IL-6 and TNF-α levels in the culture supernatants were measured using the LANCE Ultra TR-FRET Detection Kit (Perkin Elmer, Waltham, MA, USA), according to the manufacturer’s protocol. Each cytokine detection was performed separately in a 384-well plate, following the kit instructions. Samples were added at 15 μL/well to a 384-well plate and then premixed antibody solution was added at 5 μL/well. After 1 h of incubation of IL-6 and 3 h of incubation of TNF-α in the dark at 22 °C, the plates were read using an EnVision plate reader (Perkin Elmer) with the excitation wavelength at 320 nm, the donor emission were read at 615 nm, and the acceptor emission were read at 660 nm. The values of IL-6 and TNF-α levels were calculated as a percentage of control (maximal response of LPS).

### 7.8. Statistical Analysis

All experiments were performed in duplicates, in three independent experiments. Statistical analysis was performed using GraphPad Prism 8.0. All values are expressed as mean with SD. Differences among groups were evaluated via One-Way ANOVA followed by post hoc analysis (Dunnett’s multiple comparison tests) and were considered statistically significant if *p* < 0.05 (* *p* < 0.05, ** *p* < 0.01, *** *p* < 0.001, and **** *p* < 0.0001).

### 7.9. Thermodynamic Solubility Assay

Quantitative HPLC analyses were performed similarly to the chemical stability studies. A standard solution of the tested compound was prepared in methanol at a concentration of 2 mg/mL. The standard solution was diluted with methanol to obtain five solutions with different concentrations ranging from 0.06 to 0.1 mg/mL or 0.035 to 0.05 mg/mL (for **II**), and they were quantified via HPLC. The calibration curve was plotted using AUC against the concentration (mg/mL). Next, 2 mg of the tested compound was dissolved in 1 mL of Dulbecco’s phosphate-buffered saline (DPBS), and the mixture was constantly agitated at 20 °C for 24 h in a thermoshaker. After that time, the mixture was filtered through a cellulose acetate syringe filter (pore size 0.22 μm) and the solution was analyzed via HPLC. The solubility of the tested compounds was calculated using the calibration curves (mg/mL).

### 7.10. Chemical Stability Assay

Quantitative HPLC analyses were acquired using Waters Alliance e2695 separations module (Waters, Milford, CT, USA), containing 2998 photodiode array (PDA), a detector (Waters, Milford, CT, USA), and a SpeedROD RP-18e 50–4.6 mm column (Merck, KGaA, Darmstadt, Germany). The temperature of the column was preserved at 30 °C. The experiment was conducted under the following conditions: a flow rate of 5 mL/min, eluent A (water/0.1% HCOOH), eluent B (MeCN/0.1% HCOOH), and a gradient starting from 0% of B to 100% of B for 3 min. Each sample was injected at a volume of 10 μL in triplicate, and the spectra were analyzed at the maximum wavelength. Potassium phosphate buffer (100 mM, pH 7.4, + 2 mM MgCl_2_) was incubated with the test compounds (1 µM, 0.2% DMSO) at 37 °C for 120 min (N = 2). The area under the curve (AUC) was used to quantify the percentage of compound that remained after 120 min, relative to the 0 min time point.

### 7.11. Metabolic Stability Assay

Metabolic stability was assessed using pooled microsomes from the liver of male mice (CD-1) from Sigma-Aldrich (St. Louis, MO, USA). Incubations were carried out in 96-well plates. Potassium phosphate buffer (100 mM, pH 7.4, + 2 mM MgCl_2_) containing murine liver microsomes (0.5 mg protein/mL) was pre-incubated with test compounds or positive control (1 µM, 0.2% DMSO) at 37 °C for 15 min (N = 2). Reactions were initiated by adding NADPH (1 mM). The reactions were also incubated without NADPH to exclude non-NADPH metabolism. The reactions were terminated after 15 min via the addition of an acetonitrile quenching solution containing an internal standard (labetalol, 0.5 mM). The plates were centrifuged at 4000 rpm for 30 min at 4 °C, and an aliquot of supernatant was diluted with water before analysis via UPLC-MS/MS. The UPLC-MS/MS system consisted of a Waters Acquity Premier (Waters Corporation, Milford, MA, USA) coupled with a Waters Xevo TQ-S Cronos mass spectrometer (electrospray ionization mode ESI). Chromatographic separations were carried out using the Acquity UPLC BEH (bridged ethylene hybrid) C18 column (2.1 × 100 mm, and 1.7 µm particle size) equipped with Acquity UPLC BEH C18 VanGuard pre-column (2.1 × 5 mm, and 1.7 µm particle size). The column was maintained at 40 °C and eluted under gradient conditions using from 95% to 0% of eluent A over 10 min; afterwards, 100% of eluent B was added over 2.5 min, at a flow rate of 0.3 mL min^−1^. Eluent A: water/formic acid (0.1%, *v*/*v*). Eluent B: acetonitrile/formic acid (0.1%, *v*/*v*). Chromatograms were recorded using a Waters eλ PDA detector. Spectra were analyzed in the 200–700 nm range with 1.2 nm resolution and a sampling rate of 20 points/s. The MS detection settings of the Waters Xevo TQ-S Cronos mass spectrometer were as follows: source temperature, 150 °C; desolvation temperature, 250 °C; desolvation gas flow rate, 600 L h^−1^; cone gas flow, 100 L h^−1^; capillary potential, 3.00 kV; and cone potential, 30 V. Nitrogen was used as both nebulizing and drying gas. The data were obtained in a scan mode ranging from 50 to 1000 m/z, in 0.5 s intervals. The data acquisition software was MassLynx V 4.2 (Waters). The peak area ratios of analyte versus internal standard were used to calculate the percentage remaining of the test compound (%).

## 8. Conclusions

In the pursuit of novel and effective anti-Alzheimer’s therapy, the development of multifunctional ligands has emerged as a promising approach. These compounds, targeting multiple biological pathways implicated in the disease’s pathomechanism, are believed to offer enhanced therapeutic benefits. Following this strategy, we have designed new GSK-3β inhibitors with additional inhibitory activity against IKK-β and/or ROCK-1 kinases. Among the synthesized ligands, compound **62** has emerged as a remarkably potent, competitive inhibitor of GSK-3β (IC_50_ = 8 nM, K*_i_* = 2 nM), demonstrating additional inhibitory activity against ROCK-1 (IC_50_ = 2.3 µM). This compound displayed anti-inflammatory activity, decreasing the levels of NO and IL-6 in an LPS model in the BV-2 cell line, and it exhibited a neuroprotective effect in an okadaic-acid-induced tau hyperphosphorylation cell model. We also confirmed its high chemical and metabolic stability in mouse liver microsomes, as well as fair solubility, thereby confirming its potential in terms of developability.

## Figures and Tables

**Table 1:** Inhibitory activity of compounds **38**–**52** against GSK-3β, IKK-β, and ROCK-1 kinases.

Series I					Series II				
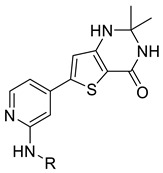					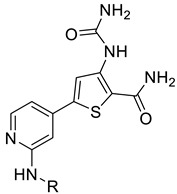				
Cmpd.	IC_50_ [nM] ^a^ or % inh. at 10 µM ^b^			R		Cmpd.	IC_50_ [nM] ^a^ or % inh. at 10 µM ^b^		
	GSK-3β	IKK-β	ROCK-1				GSK-3β	IKK-β	ROCK-1
**38**	1130 ± 43	6524 ± 281	50% ± 0	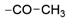		**47**	34% ± 9	17% ± 8	nd
**39**	12 ± 0	33% ± 6	32% ± 3	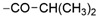		**48**	22 ± 0	<10%	<10%
**40**	10 ± 0	4380 ± 544	1760 ± 109	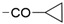		**49**	20 ± 0	16% ± 3	14% ^d^ ± 3
**41**	32 ± 1	32% ± 4	48% ± 3	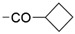		**50**	34 ± 0	<10%	<10% ^d^
**42**	830 ± 30	18% ± 3	30% ± 2	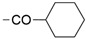		**51**	614 ± 26	<10%	11% ^d^ ± 0
**43**	1314 ± 42	38% ± 5	3742 ± 217	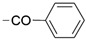		**52**	398 ± 12	30% ± 2	15% ± 7
**44**	32% ± 1	<10%	<10%	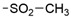		-			
**45**	40% ± 9	<10%	<10%	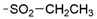		-			
**46**	39% ± 2	41% ± 2	1729 ± 78	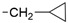		-			
**I**	161 ± 2	46%	119 ± 6	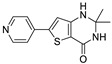					
**II**	5 ± 0	22% ± 7	nd	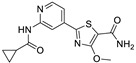					
**III**	47% ± 9	405 ± 9	nd	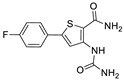					
**IV ^c^**	80 ± 2	894 ± 42	nd	**Staurosporine**					

^a^ Half maximal inhibitory concentration of the tested compound for selected kinase, mean value ± standard error of the mean (SEM) of triplicates; ^b^ mean value ± standard deviation (SD) of triplicates; ^c^ reference, Biokom, Janki, Poland; ^d^ tested at inhibitor screening concentration of 1 μM.

**Table 2:** Inhibitory activity of compounds **53**–**59** against GSK-3β, IKK-β, and ROCK-1 kinases.

Series III					Series IV					
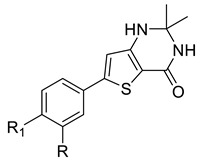					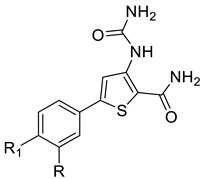					
Cmpd.	IC_50_ [nM] ^a^ or % inh. at 10 µM ^b^			R		R_1_	Cmpd.	IC_50_ [nM] ^a^ or % inh. at 10 µM ^b^		
	GSK-3β	IKK-β	ROCK-1					GSK-3β	IKK-β	ROCK-1
**53**	47% ± 4	<10%	<10%	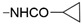		-	**58**	36% ± 8	422 ± 12	16% ^c^ ± 2
**54**	453 ± 13	10% ± 7	nd	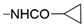		F	**59**	1847 ± 38	7621 ± 695	<10%
**55**	11% ± 3	16% ± 0	<10%	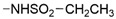		-	-			
**56**	<10%	<10%	33% ± 2	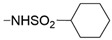		-	-			
**57**	<10%	17% ± 7	<10%	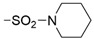		-	-			

^a^ Half maximal inhibitory concentration of the tested compound for selected kinase, mean value ± standard error of the mean (SEM) of triplicates; ^b^ mean value ± standard deviation (SD) of triplicates; ^c^ tested at inhibitor screening concentration of 1 μM.

**Table 3:** Inhibitory activity of compounds **40** and **60**–**62** against GSK-3β, IKK-β, and ROCK-1 kinases.

Series V				
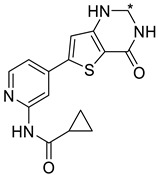				
Cmpd.	*	IC_50_ [nM] ^a^ or % inh. at 10 µM ^b^		
		GSK-3β	IKK-β	ROCK-1
**40**	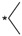	10 ± 0	4380 ± 544	1760 ± 109
**60**	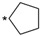	10 ± 0	7351	1361 ± 79
**61**	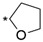	12 ± 0	<10%	40% ± 3
**62**	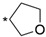	8 ± 0	39% ± 9	2310 ± 131

^a^ Half maximal inhibitory concentration of the tested compound for selected kinase, mean value ± standard error of the mean (SEM) of triplicates; ^b^ mean value ± standard deviation (SD) of triplicates; * substituent.

**Table 4:** Cytotoxicity of compounds **39**–**41**, **48**–**50**, **58**, **60**, **62** and **II** in HT-22 and BV-2 cells.

Compound	Cytotoxicity in HT-22 Cells $({\mathrm{IC}}_{50}\;\bar{x}$ ± SEM µM)	Cytotoxicity in BV-2 Cells (${\mathrm{IC}}_{50}\;\bar{x}$ ± SEM µM)
**39**	8.4 ± 5.3	4.5 ± 1.9
**40**	19.9 ± 4.1	3.6 ± 0.7
**41**	>100	6.1 ± 3.1
**48**	36.6 ± 4.3	21.8 ± 3.8
**49**	>100	>100
**50**	>100	>100
**58**	67.1 ± 14.5	51.4 ± 0.8
**60**	9.7 ± 5.8	3.0 ± 0.0
**62**	58.3 ± 10.0	54.8 ± 1.9
**II**	>100	15.4 ± 0.6

Data expressed as the means ± SEM; N ≥ 6.

**Table 5:** Solubility and metabolic stability data for compounds **62** and **II**.

Compd.	Solubility (µg/mL) ^a^ ± SD	MLM Stability (%) ^b^ ± SD
**62**	31 ± 1	100 ± 1
**II**	5 ± 1	100 ± 0

^a^ Result expressed as a concentration of the compound in Dulbecco’s phosphate-buffered saline (DPBS) after 24 h shaking at room temperature. ^b^ The results expressed are as the percentage of the compound that remains after 15 min of incubation with mouse liver microsomes (MLMs). Verapamil was used as a positive control (35% compound remaining after 15 min). Values are means of at least two experiments ± SD.

## Data Availability

The data presented in this study are available in article and Supplementary Materials.

## Supplementary Materials

The following supporting information can be downloaded at: https://www.mdpi.com/article/10.3390/molecules29112616/s1, Figure S1. Lineweaver–Burk (A) and Cornish–Bowden (B) plots revealing ATP-competitive type of GSK-3β inhibition by compound **62**; V = initial velocity rate, S = ATP concentration, I = inhibitor concentration. Figure S2. Determination of K_i_ value for ATP-competitive inhibitor **62**. Data replot from Lineweaver-Burk plots. Determined K_i_ = 2.22 × 10^−9^, pK_i_ = 8.65; I = inhibitor concentration, K_m_ = Michaelis-Menten constant. Table S1. Inhibitory activity of compound 62 against panel of selected CMGC kinases. Table S2. Cytotoxic effects of selected inhibitors in HT-22 mouse hippocampal neuronal cells and BV-2 mouse microglial cells.

## References

[1] (2022). Alzheimer’s Association 2022 Alzheimer’s Disease Facts and Figures. Alzheimer’s Dement..

[2] Wong W. (2020). Economic Burden of Alzheimer Disease and Managed Care Considerations. Am. J. Manag. Care.

[3] Livingston G., Huntley J., Sommerlad A., Ames D., Ballard C., Banerjee S., Brayne C., Burns A., Cohen-Mansfield J., Cooper C. (2020). Dementia Prevention, Intervention, and Care: 2020 Report of the Lancet Commission. Lancet.

[4] Arnsten A.F.T., Datta D., Del Tredici K., Braak H. (2021). Hypothesis: Tau Pathology Is an Initiating Factor in Sporadic Alzheimer’s Disease. Alzheimer’s Dement..

[5] Hampel H., Hardy J., Blennow K., Chen C., Perry G., Kim S.H., Villemagne V.L., Aisen P., Vendruscolo M., Iwatsubo T. (2021). The Amyloid-β Pathway in Alzheimer’s Disease. Mol. Psychiatry.

[6] Leng F., Edison P. (2021). Neuroinflammation and Microglial Activation in Alzheimer Disease: Where Do We Go from Here?. Nat. Rev. Neurol..

[7] de Oliveira J., Kucharska E., Garcez M.L., Rodrigues M.S., Quevedo J., Moreno-Gonzalez I., Budni J. (2021). Inflammatory Cascade in Alzheimer’s Disease Pathogenesis: A Review of Experimental Findings. Cells.

[8] van der Kant R., Goldstein L.S.B., Ossenkoppele R. (2020). Amyloid-β-Independent Regulators of Tau Pathology in Alzheimer Disease. Nat. Rev. Neurosci..

[9] Tiwari S., Atluri V., Kaushik A., Yndart A., Nair M. (2019). Alzheimer’s Disease: Pathogenesis, Diagnostics, and Therapeutics. Int. J. Nanomed..

[10] Chen G.F., Xu T.H., Yan Y., Zhou Y.R., Jiang Y., Melcher K., Xu H.E. (2017). Amyloid Beta: Structure, Biology and Structure-Based Therapeutic Development. Acta Pharmacol. Sin..

[11] Maccioni R.B., Farías G., Morales I., Navarrete L. (2010). The Revitalized Tau Hypothesis on Alzheimer’s Disease. Arch. Med. Res..

[12] Hooper C., Killick R., Lovestone S. (2008). The GSK3 Hypothesis of Alzheimer’s Disease. J. Neurochem..

[13] Song L., Oseid D.E., Wells E.A., Robinson A.S. (2022). The Interplay between GSK3β and Tau Ser262 Phosphorylation during the Progression of Tau Pathology. Int. J. Mol. Sci..

[14] Ballatore C., Lee V.M.Y., Trojanowski J.Q. (2007). Tau-Mediated Neurodegeneration in Alzheimer’s Disease and Related Disorders. Nat. Rev. Neurosci..

[15] Onishi T., Iwashita H., Uno Y., Kunitomo J., Saitoh M., Kimura E., Fujita H., Uchiyama N., Kori M., Takizawa M. (2011). A Novel Glycogen Synthase Kinase-3 Inhibitor 2-methyl-5-(3-{4-[(*S*)-}-1--5-)-1,3,4- Decreases Tau Phosphorylation and Ameliorates Cognitive Deficits in a Transgenic Model of Alzheimer’s Disease. J. Neurochem..

[16] Bao X.Q., Li N., Wang T., Kong X.C., Tai W.J., Sun H., Zhang D. (2013). FLZ Alleviates the Memory Deficits in Transgenic Mouse Model of Alzheimer’s Disease via Decreasing Beta-Amyloid Production and Tau Hyperphosphorylation. PLoS ONE.

[17] Chen C.H., Zhou W., Liu S., Deng Y., Cai F., Tone M., Tone Y., Tong Y., Song W. (2012). Increased Signalling up-Regulates BACE1 Expression and Its Therapeutic Potential in Alzheimer’s Disease. Int. J. Neuropsychopharmacol..

[18] Ly P.T.T., Wu Y., Zou H., Wang R., Zhou W., Kinoshita A., Zhang M., Yang Y., Cai F., Woodgett J. (2013). Inhibition of GSK3β-Mediated BACE1 Expression Reduces Alzheimer-Associated Phenotypes. J. Clin. Invest..

[19] Avrahami L., Farfara D., Shaham-Kol M., Vassar R., Frenkel D., Eldar-Finkelman H. (2013). Inhibition of Glycogen Synthase Kinase-3 Ameliorates β-Amyloid Pathology and Restores Lysosomal Acidification and Mammalian Target of Rapamycin Activity in the Alzheimer Disease Mouse Model: In Vivo and In Vitro Studies. J. Biol. Chem..

[20] Ding Y., Qiao A., Fan G.H. (2010). Indirubin-3’-monoxime Rescues Spatial Memory Deficits and Attenuates β-Amyloid-Associated Neuropathology in a Mouse Model of Alzheimer’s Disease. Neurobiol. Dis..

[21] Gianferrara T., Cescon E., Grieco I., Spalluto G., Federico S. (2022). Glycogen Synthase Kinase 3β Involvement in Neuroinflammation and Neurodegenerative Diseases. Curr. Med. Chem..

[22] Xu M., Wang S.L., Zhu L., Wu P.Y., Dai W.B., Rakesh K.P. (2019). Structure-Activity Relationship (SAR) Studies of Synthetic Glycogen Synthase Kinase-3β Inhibitors: A Critical Review. Eur. J. Med. Chem..

[23] Wei J., Wang J., Zhang J., Yang J., Wang G., Wang Y. (2022). Development of Inhibitors Targeting Glycogen Synthase Kinase-3β for Human Diseases: Strategies to Improve Selectivity. Eur. J. Med. Chem..

[24] Shri S.R., Manandhar S., Nayak Y., Pai K.S.R. (2023). Role of GSK-3β Inhibitors: New Promises and Opportunities for Alzheimer’s Disease. Adv. Pharm. Bull..

[25] Balboni B., Masi M., Rocchia W., Girotto S., Cavalli A. (2023). GSK-3β Allosteric Inhibition: A Dead End or a New Pharmacological Frontier?. Int. J. Mol. Sci..

[26] Cheng Z., Han T., Yao J., Wang K., Dong X., Yu F., Huang H., Han M., Liao Q., He S. (2024). Targeting Glycogen Synthase Kinase-3β for Alzheimer’s Disease: Recent Advances and Future Prospects. Eur. J. Med. Chem..

[27] Arciniegas Ruiz S.M., Eldar-Finkelman H. (2022). Glycogen Synthase Kinase-3 Inhibitors: Preclinical and Clinical Focus on CNS-A Decade Onward. Front. Mol. Neurosci..

[28] Serenó L., Coma M., Rodríguez M., Sánchez-Ferrer P., Sánchez M.B., Gich I., Agulló J.M., Pérez M., Avila J., Guardia-Laguarta C. (2009). A Novel GSK-3β Inhibitor Reduces Alzheimer’s Pathology and Rescues Neuronal Loss In Vivo. Neurobiol. Dis..

[29] DaRocha-Souto B., Coma M., Pérez-Nievas B.G., Scotton T.C., Siao M., Sánchez-Ferrer P., Hashimoto T., Fan Z., Hudry E., Barroeta I. (2013). Activation of Glycogen Synthase Kinase-3 Beta Mediates β-Amyloid Induced Neuritic Damage in Alzheimer’s Disease. Neurobiol. Dis..

[30] Lovestone S., Boada M., Dubois B., Hüll M., Rinne J.O., Huppertz H.J., Calero M., Andrés M.V., Gómez-Carrillo B., León T. (2015). A Phase II Trial of Tideglusib in Alzheimer’s Disease. J. Alzheimer’s Dis..

[31] Henderson B.W., Gentry E.G., Rush T., Troncoso J.C., Thambisetty M., Montine T.J., Herskowitz J.H. (2016). Rho-Associated Protein Kinase 1 (ROCK1) Is Increased in Alzheimer’s Disease and ROCK1 Depletion Reduces Amyloid-β Levels in Brain. J. Neurochem..

[32] Hu Y.B., Ren R.J., Zhang Y.F., Huang Y., Cui H.L., Ma C., Qiu W.Y., Wang H., Cui P.J., Chen H.Z. (2019). Rho-Associated Coiled-Coil Kinase 1 Activation Mediates Amyloid Precursor Protein Site-Specific Ser655 Phosphorylation and Triggers Amyloid Pathology. Aging Cell.

[33] Herskowitz J.H., Feng Y., Mattheyses A.L., Hales C.M., Higginbotham L.A., Duong D.M., Montine T.J., Troncoso J.C., Thambisetty M., Seyfried N.T. (2013). Pharmacologic Inhibition of ROCK2 Suppresses Amyloid-β Production in an Alzheimer’s Disease Mouse Model. J. Neurosci..

[34] Hamano T., Shirafuji N., Yen S.H., Yoshida H., Kanaan N.M., Hayashi K., Ikawa M., Yamamura O., Fujita Y., Kuriyama M. (2020). Rho-Kinase ROCK Inhibitors Reduce Oligomeric Tau Protein. Neurobiol. Aging.

[35] Shibuya M., Hirai S., Seto M., Satoh S., Ohtomo E. (2005). Effects of Fasudil in Acute Ischemic Stroke: Results of a Prospective Placebo-Controlled Double-Blind Trial. J. Neurol. Sci..

[36] Wong-Guerra M., Calfio C., Maccioni R.B., Rojo L.E. (2023). Revisiting the Neuroinflammation Hypothesis in Alzheimer’s Disease: A Focus on the Druggability of Current Targets. Front. Pharmacol..

[37] Sun E., Motolani A., Campos L., Lu T. (2022). The Pivotal Role of NF-κB in the Pathogenesis and Therapeutics of Alzheimer’s Disease. Int. J. Mol. Sci..

[38] Sivamaruthi B.S., Raghani N., Chorawala M., Bhattacharya S., Prajapati B.G., Elossaily G.M., Chaiyasut C. (2023). NF-κB Pathway and Its Inhibitors: A Promising Frontier in the Management of Alzheimer’ s Disease. Biomedicines.

[39] Gutierrez H., Davies A.M. (2011). Regulation of Neural Process Growth, Elaboration and Structural Plasticity by NF-κB. Trends Neurosci..

[40] Ramadass V., Vaiyapuri T., Tergaonkar V. (2020). Small Molecule NF-κB Pathway Inhibitors in Clinic. Int. J. Mol. Sci..

[41] Zhang J., Zhang R., Li W., Ma X.C., Qiu F., Sun C.P. (2023). IκB Kinase β (IKKβ): Structure, Transduction Mechanism, Biological Function, and Discovery of Its Inhibitors. Int. J. Biol. Sci..

[42] Awasthee N., Rai V., Chava S., Nallasamy P., Kunnumakkara A.B., Bishayee A., Chauhan S.C., Challagundla K.B., Gupta S.C. (2019). Targeting IκappaB Kinases for Cancer Therapy. Semin. Cancer Biol..

[43] Llona-Minguez S., Baiget J., Mackay S.P. (2013). Small-Molecule Inhibitors of IκB Kinase (IKK) and IKK-Related Kinases. Pharm. Pat. Anal..

[44] Liu Y., Liu X., Hao W., Decker Y., Schomburg R., Fülöp L., Pasparakis M., Menger M.D., Fassbender K. (2014). IKKβ Deficiency in Myeloid Cells Ameliorates Alzheimer’s Disease-Related Symptoms and Pathology. J. Neurosci..

[45] Schnöder L., Quan W., Yu Y., Tomic I., Luo Q., Hao W., Peng G., Li D., Fassbender K., Liu Y. (2023). Deficiency of IKKβ in Neurons Ameliorates Alzheimer’s Disease Pathology in APP- and Tau-Transgenic Mice. FASEB J..

[46] Li D.D., Zhang Y.H., Zhang W., Zhao P. (2019). Meta-Analysis of Randomized Controlled Trials on the Efficacy and Safety of Donepezil, Galantamine, Rivastigmine, and Memantine for the Treatment of Alzheimer’s Disease. Front. Neurosci..

[47] Salloway S., Chalkias S., Barkhof F., Burkett P., Barakos J., Purcell D., Suhy J., Forrestal F., Tian Y., Umans K. (2022). Amyloid-Related Imaging Abnormalities in 2 Phase 3 Studies Evaluating Aducanumab in Patients with Early Alzheimer Disease. JAMA Neurol..

[48] van Dyck C.H., Swanson C.J., Aisen P., Bateman R.J., Chen C., Gee M., Kanekiyo M., Li D., Reyderman L., Cohen S. (2023). Lecanemab in Early Alzheimer’s Disease. N. Engl. J. Med..

[49] Kurasawa O., Oguro Y., Miyazaki T., Homma M., Mori K., Iwai K., Hara H., Skene R., Hoffman I., Ohashi A. (2017). Identification of a New Class of Potent Cdc7 Inhibitors Designed by Putative Pharmacophore Model: Synthesis and Biological Evaluation of 2,3-dihydrothieno[3,2-*d*]pyrimidin-4(1*H*)-ones. Bioorganic Med. Chem..

[50] Sivaprakasam P., Han X., Civiello R.L., Jacutin-Porte S., Kish K., Pokross M., Lewis H.A., Ahmed N., Szapiel N., Newitt J.A. (2015). Discovery of New Acylaminopyridines as GSK-3 Inhibitors by a Structure Guided in-Depth Exploration of Chemical Space around a Pyrrolopyridinone Core. Bioorganic Med. Chem. Lett..

[51] Baxter A., Brough S., Cooper A., Floettmann E., Foster S., Harding C., Kettle J., McInally T., Martin C., Mobbs M. (2004). Hit-to-Lead Studies: The Discovery of Potent, Orally Active, Thiophenecarboxamide IKK-2 Inhibitors. Bioorganic Med. Chem. Lett..

[52] Zegzouti H., Zdanovskaia M., Hsiao K., Goueli S.A. (2009). ADP-Glo: A Bioluminescent and Homogeneous ADP Monitoring Assay for Kinases. Assay Drug Dev. Technol..

[53] Becker W., Sippl W. (2011). Activation, Regulation, and Inhibition of DYRK1A. FEBS J..

[54] Ryu Y.S., Park S.Y., Jung M.S., Yoon S.H., Kwen M.Y., Lee S.Y., Choi S.H., Radnaabazar C., Kim M.K., Kim H. (2010). Dyrk1A-Mediated Phosphorylation of Presenilin 1: A Functional Link between Down Syndrome and Alzheimer’s Disease. J. Neurochem..

[55] Chen Z., Chen B., Xu W.F., Liu R.F., Yang J., Yu C.X. (2012). Effects of PTEN Inhibition on Regulation of Tau Phosphorylation in an Okadaic Acid-Induced Neurodegeneration Model. Int. J. Dev. Neurosci..

[56] Wang J., Tung Y.C., Wang Y., Li X.T., Iqbal K., Grundke-Iqbal I. (2001). Hyperphosphorylation and Accumulation of Neurofilament Proteins in Alzheimer Disease Brain and in Okadaic Acid-Treated SY5Y Cells. FEBS Lett..

[57] Liu W., Wu L., Li D., Huang Y., Liu M., Liu W., Tian C., Liu X., Jiang X., Hu X. (2022). Discovery of Novel Tacrine Derivatives as Potent Antiproliferative Agents with CDKs Inhibitory Property. Bioorg. Chem..

[58] Sudau A., Es-Sayed M., Braun C.A., Meissner R., Sirven C., Benting J., Dahmen P., Portz D., Wachendorff-Neumann U., Desbordes P. (2011). Phenylpyri(mi)dinylazoles.

[59] Sudau A., Es-Sayed M., Braun C.A., Meissner R., Sirven C., Benting J., Dahmen P., Portz D., Wachendorff-Neumann U., Desbordes P. (2011). Phenylpyri(mi)dinylazoles. U.S. Patent.

[60] Hou S., Yang X., Yang Y., Tong Y., Chen Q., Wan B., Wei R., Lu T., Chen Y., Hu Q. (2021). Design, Synthesis and Biological Evaluation of 1*H*-Indazole Derivatives as Novel ASK1 Inhibitors. Eur. J. Med. Chem..

[61] Houze J.B., Dransfield P., Pattaropong V., Du X., Fu Z., Lai S., Park J., Jiao X., Kohn T.J., Aicher T.D. (2013). Urea Compounds as GKA Activators. WO.

[62] Sutherland M., Li A., Kaghad A., Panagopoulos D., Li F., Szewczyk M., Smil D., Scholten C., Bouché L., Stellfeld T. (2021). Rational Design and Synthesis of Selective PRMT4 Inhibitors: A New Chemotype for Development of Cancer Therapeutics. ChemMedChem.

[63] Pan Z., Chen Y., Liu J., Jiang Q., Yang S., Guo L., He G. (2018). Design, Synthesis, and Biological Evaluation of Polo-like Kinase 1/Eukaryotic Elongation Factor 2 Kinase (PLK1/EEF2K) Dual Inhibitors for Regulating Breast Cancer Cells Apoptosis and Autophagy. Eur. J. Med. Chem..

[64] Castro A.C., Chan K., Evans C.A., Janardanannair S., Lescarbeau A., Li L., Liu T., Liu Y., Ren P., Snyder D.A. (2013). Heterocyclic Compounds and Uses Thereof. U.S. Patent.

[65] Castro A.C., Evans C.A., Janardanannair S., Lescarbeau A., Liu T., Snyder D.A., Tremblay M.R., Ren P., Liu Y., Li L. (2013). Heterocyclic Compounds and Uses Thereof. U.S. Patent.

[66] Qu B., Xu Y., Lu Y., Zhuang W., Jin X., Shi Q., Yan S., Guo Y., Shen Z., Che J. (2022). Design, Synthesis and Biological Evaluation of Sulfonamides Inhibitors of XPO1 Displaying Activity against Multiple Myeloma Cells. Eur. J. Med. Chem..

[67] Boys M.L., Schretzman L.A., Tollefson M.B., Chandrakumar N.S., Khanna I.K., Nguyen M., Downs V., Mohler S.B., Gesicki G.J., Penning T.D. (2004). Heteroarylalkanoic Acids as Integrin Receptor Antagonists. WO.

[68] Oguro Y., Kurasawa O. (2010). Thienopyrimidine as CDC7 Kinase Inhibitors. WO.

[69] Lu C., Wu C., Ghoreishi D., Chen W., Wang L., Damm W., Ross G.A., Dahlgren M.K., Russell E., Von Bargen C.D. (2021). OPLS4: Improving Force Field Accuracy on Challenging Regimes of Chemical Space. J. Chem. Theory Comput..

[70] Pettersen E.F., Goddard T.D., Huang C.C., Meng E.C., Couch G.S., Croll T.I., Morris J.H., Ferrin T.E. (2021). UCSF ChimeraX: Structure Visualization for Researchers, Educators, and Developers. Tools Protein Sci..

[71] Goddard T.D., Huang C.C., Meng E.C., Pettersen E.F., Couch G.S., Morris J.H., Ferrin T.E. (2018). UCSF ChimeraX: Meeting Modern Challenges in Visualization and Analysis. Tools Protein Sci..

[72] PrestoBlue™ Cell Viability Reagent. https://assets.thermofisher.com/TFS-Assets/LSG/manuals/MAN0018370-PrestoBlueCellViabilityReagent-PI.pdf.

[73] Nussler A.K., Glanemann M., Schirmeier A., Liu L., Nüssler N.C. (2006). Fluorometric Measurement of Nitrite/Nitrate by 2,3-Diaminonaphthalene. Nat. Protoc..

