# Supplementary material for: Connecting GSK-3β Inhibitory Activity with IKK-β or ROCK-1 Inhibition to Target Tau Aggregation and Neuroinflammation in Alzheimer’s Disease—Discovery, In Vitro and In Cellulo Activity of Thiazole-Based Inhibitors
Source: Molecules. 2024 Jun 2;29(11):2616. doi: 10.3390/molecules29112616 (PMC11173485; doi:10.3390/molecules29112616)
Supplement: Supplementary file 1 [file molecules-29-02616-s001.zip › molecules-3011921-supplementary.pdf]

Supporting information for

Connecting GSK-3β inhibitory activity with IKK-β or ROCK-1 inhibition to target tau aggregation and neuroinflammation in Alzheimer's disease – discovery, *in vitro* and *cellulo* activity of thiazole-based inhibitors

Izabella Góral[a,b], Tomasz Wichur[a], Emilia Sługocka[a,b], Justyna Godyń[a], Natalia Szałaj[a], Paula Zaręba[a], Monika Głuch-Lutwin[c], Barbara Mordyl[c], Dawid Panek[a], Anna Więckowska[a,*]

[a] Department of Physicochemical Drug Analysis, Faculty of Pharmacy, Jagiellonian University Medical College, 9 Medyczna St., 30-688 Krakow, Poland

[b] Doctoral School of Medical and Health Sciences, Jagiellonian University Medical College, 16 Lazarza St., 31-530 Krakow, Poland

[c] Department of Pharmacobiology, Faculty of Pharmacy, Jagiellonian University Medical College, 9 Medyczna St., 30-688 Krakow, Poland

[*]Corresponding Author:
e-mail addresses: anna.wieckowska@uj.edu.pl (Anna Więckowska)

Contents:

Kinetic studies of GSK-3β inhibition by compound **62**.. 3

Selectivity studies for compound **62**……………………………………………………...…4

Cytotoxic effects of selected inhibitors in HT-22 mouse hippocampal neuronal cells and BV-2 mouse microglial cells... 5

LCMS chromatograms and ^{1}H and ^{13}C NMR spectra of final compounds................................ 6

Kinetic studies of GSK-3β inhibition by compound 62

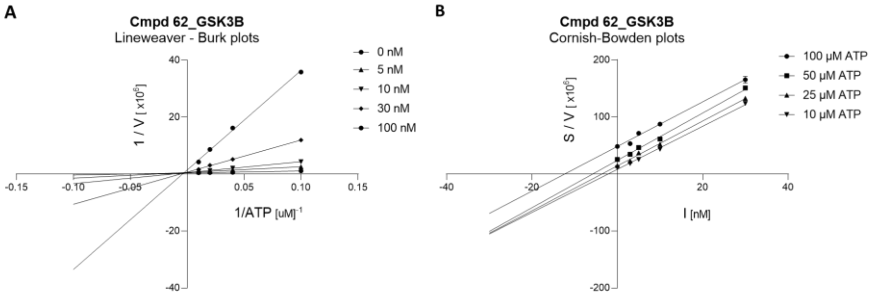

Figure S1. Lineweaver−Burk **(A)** and Cornish−Bowden **(B)** plots revealing **ATP-competitive type** of GSK-3β inhibition by compound **62**; V = initial velocity rate, S = ATP concentration, I = inhibitor concentration.

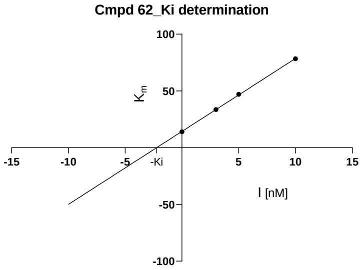

Figure S2. Determination of K_i value for **ATP-competitive** inhibitor **62**. Data replot from Lineweaver-Burk plots. Determined $K_i = 2.22\times10^{-9}$, $pK_i = 8.65$; I = inhibitor concentration, K_m = Michaelis-Menten constant.

Selectivity studies for compound 62

Selectivity studies were performed at Eurofins in Kinase Enzymatic Radiometric [Km ATP] Kinase Profiler Lead Hunter Assay. The percentage of enzymatic activity was measured at 1 µM of compound **62** in duplicate for 17 selected kinases.

Table S1. Inhibitory activity of compound **62** against panel of selected CMGC kinases.

kinase	% of enzyme activity at 1 µM of compound 62±SD*
CDK1/cyclinB	33±1
CDK2/cyclinA	26±2
CDK4/cyclinD3	83±5
CDK6/cyclinD3	88±0
CDK7/cyclinH/ MAT1	57±1
CDK9/cyclin T1	16±2
DYRK1A	1±1
DYRK1B	1±1
GSK-3α	0±0
GSK-3β	1±1
JNK2α2	89±14
JNK3	21±2
MAPK1	37±2
MAPK2	73±7
SAPK2a(T106M)	80±1
SAPK2b	98±1
SAPK3	22±3

*Where n = 2, the value reported here is actually range / $\sqrt{2}$.

Cytotoxic effects of selected inhibitors in HT-22 mouse hippocampal neuronal cells and BV-2 mouse microglial cells

Table S2. Cytotoxic effects of selected inhibitors in HT-22 mouse hippocampal neuronal cells and BV-2 mouse microglial cells.

	neurotoxicity in HT-22 cells[a]						immunotoxicity in BV-2 cells[a]					
C [µM]	**0,1**	**1**	**10**	**50**	**100**	**IC_{50}[b] [µM]**	**0,1**	**1**	**10**	**50**	**100**	**IC_{50}[b] [µM]**
log C	-7	-6	-5	-4.3	-4		-7	-6	-5	-4.3	-4	
Cmpd												
**39**	91% ±1	84% ±2	55% ±3	35% ±11	29% ±6	8.4 ±5.3	105% ±4[c]	107% ±1[c]	105% ±3[c]	73% ±3[c]	14% ±0[c]	4.5 ±1.9
**40**	100% ±3	105% ±5	71% ±4	28% ±10	21% ±3	19.9 ±4.1	106% ±10	109% ±11	9% ±2	2% ±0	2% ±0	3.6 ±0.7
**41**	94% ±8	83% ±8	62% ±4	36% ±5	49% ±9	>100	104% ±9	94% ±1	8% ±0	1% ±0	0% ±0	6.1 ±3.1
**48**	92% ±7	89% ±11	77% ±12	52% ±17	nt	36.6 ±4.3	103% ±3	97% ±4	66% ±9	20% ±8	nt	21.8 ±3.8
**49**	95% ±1	102% ±8	101% ±4	88% ±12	76% ±8	>100	107% ±3[d]	106% ±2[d]	107% ±3[d]	100% ±9[d]	74% ±10[d]	>100
**50**	112% ±9	112% ±16	96% ±11	94% ±18	70% ±15	>100	101% ±11	107% ±11	114% ±9	96% ±13	50% ±0	>100
**58**	94% ±7	92% ±8	89% ±9	54% ±8	45% ±6	67.1 ±14.5	106% ±0	107% ±5	97% ±2	52% ±6	13% ±2	51.4 ±0.8
**60**	101% ±3	105% ±8	62% ±3	36% ±9	31% ±6	9.7 ±5.8	108% ±5	101% ±4	9% ±1	3% ±0	3% ±1	3.0 ±0.0
**62**	106% ±5	108% ±9	105% ±7	35% ±12	29% ±9	58.3 ±10.0	105% ±11	106% ±7	105% ±10	5% ±1	2% ±0	54.8 ±1.9
Ref.												
**II**	107% ±14	104% ±20	87% ±20	77% ±13	64% ±10	>100	97% ±6	102% ±9	60% ±9	27% ±12	4% ±1	15.4 ±0.6

[a] % of cell viability at the given concentration of tested compound; mean value ± standard deviation (SD) of triplicates; [b] half maximal inhibitory concentration of the tested compound; mean value ± standard error of the mean (SEM) of triplicates; [c] data obtained in log C of **39** of -8.5; -7.5; -6.5; -5.5; -5, respectively; [d] data obtained in log C of **49** of -7.5; -6.5; -5.5; -4.5; -4. respectively; nt, not tested.

LCMS chromatograms and ^{1}H and ^{13}C NMR spectra of final compounds

N-(4-(2,2-dimethyl-4-oxo-1,2,3,4-tetrahydrothieno[3,2-*d*]pyrimidin-6-yl)pyridin-2-yl)acetamide (**38**)

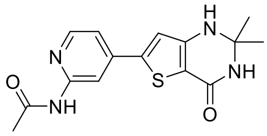

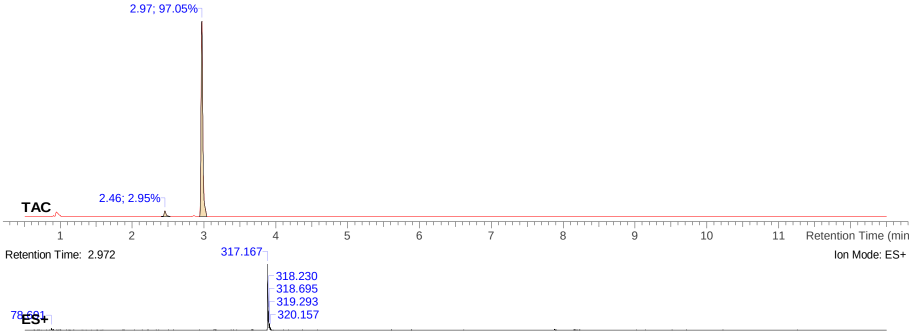

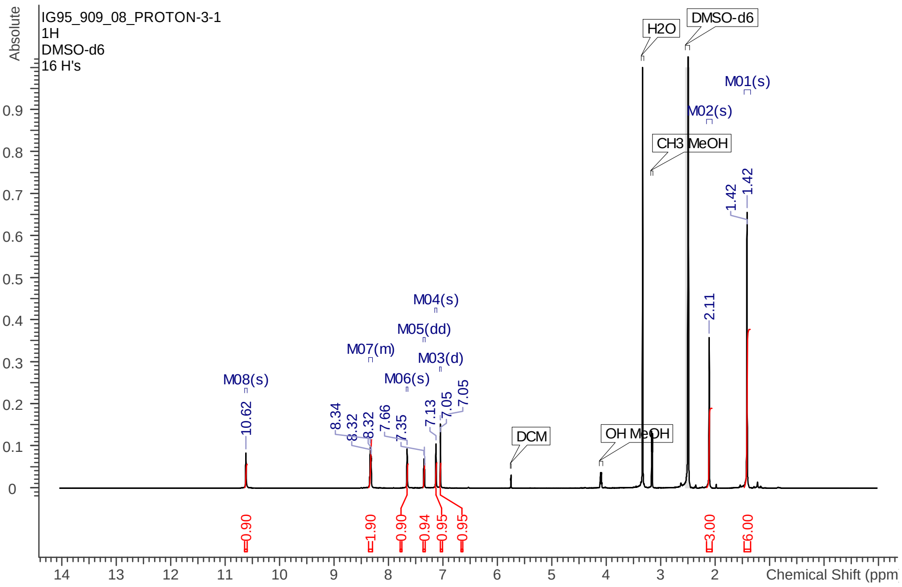

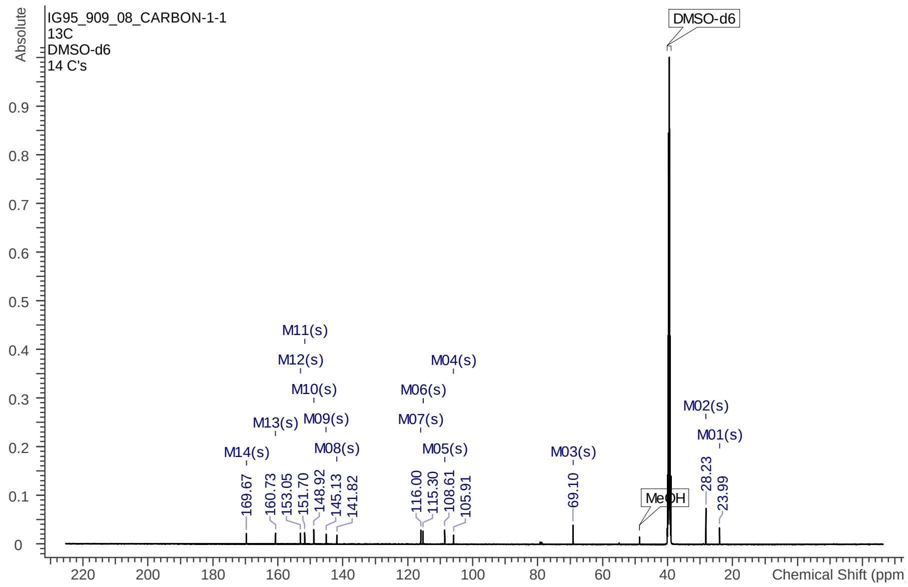

N-(4-(2,2-dimethyl-4-oxo-1,2,3,4-tetrahydrothieno[3,2-*d*]pyrimidin-6-yl)pyridin-2-yl)isobutyramide (**39**)

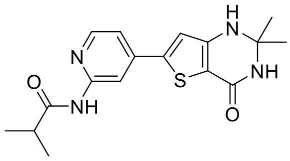

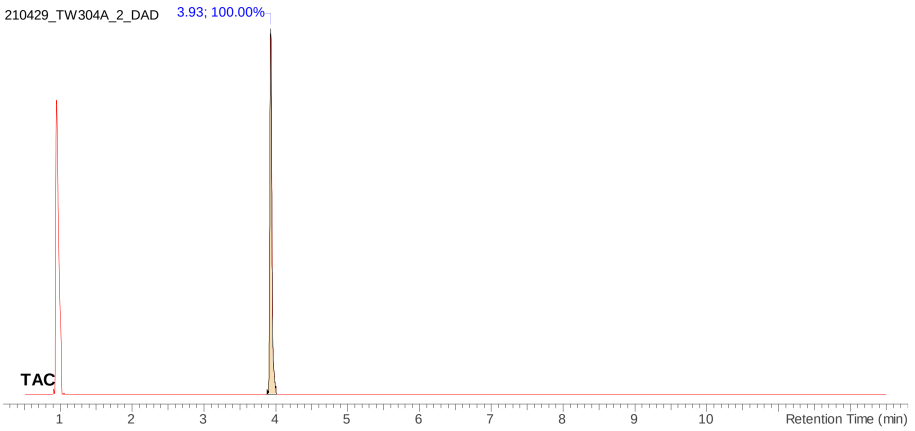
210429_TW304A_2_DAD
3.93; 100.00%
TAC
Retention Time (min)

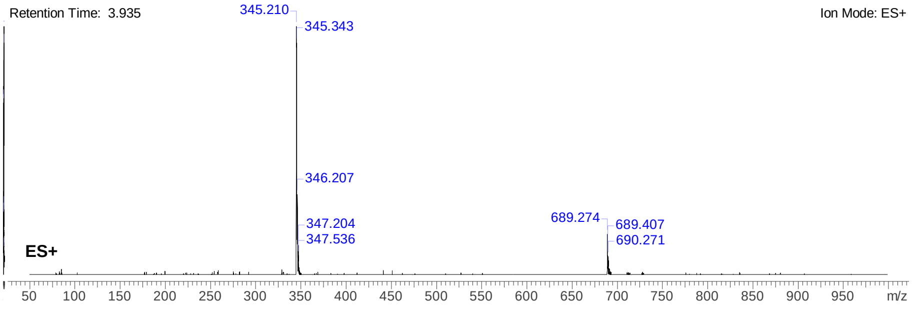
Retention Time: 3.935
Ion Mode: ES+
345.210
345.343
346.207
347.204
347.536
689.274
689.407
690.271
ES+
m/z

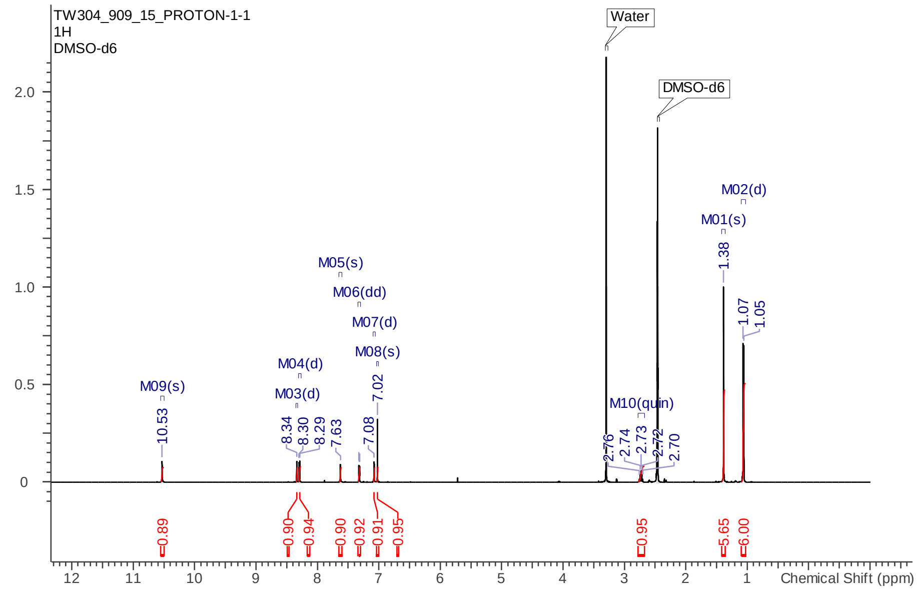
TW304_909_15_PROTON-1-1
1H
DMSO-d6
Water
DMSO-d6
M02(d)
M01(s)
1.38
1.07
1.05
M05(s)
M06(dd)
M07(d)
M08(s)
M04(d)
M03(d)
M09(s)
10.53
8.34
8.30
8.29
7.63
7.08
7.02
M10(quin)
2.76
2.74
2.73
2.72
2.70
0.89
0.90
0.94
0.90
0.92
0.91
0.95
0.95
5.65
6.00
Chemical Shift (ppm)

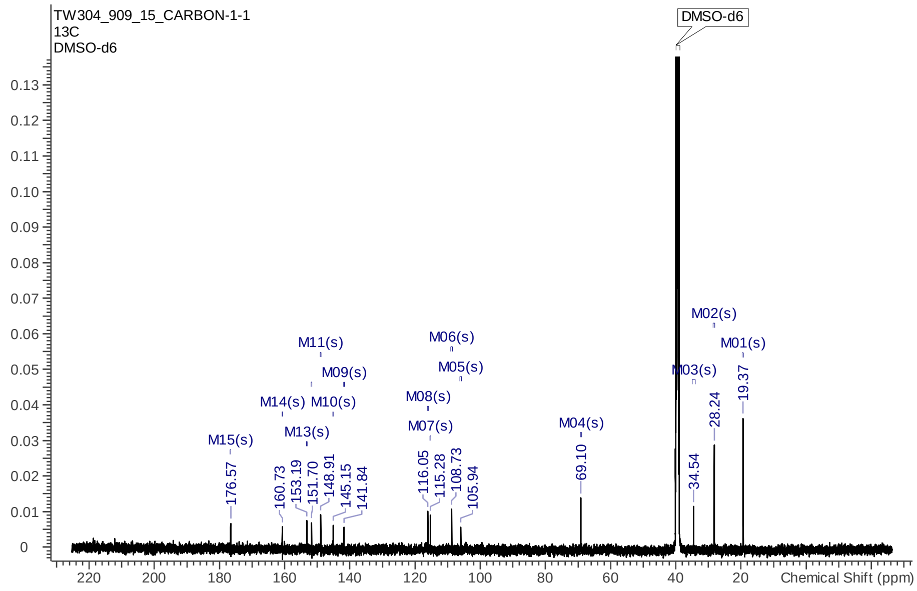

N-(4-(2,2-dimethyl-4-oxo-1,2,3,4-tetrahydrothieno[3,2-*d*]pyrimidin-6-yl)pyridin-2-yl)-cyclopropanecarboxamide (**40**)

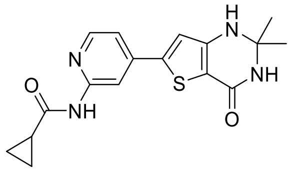

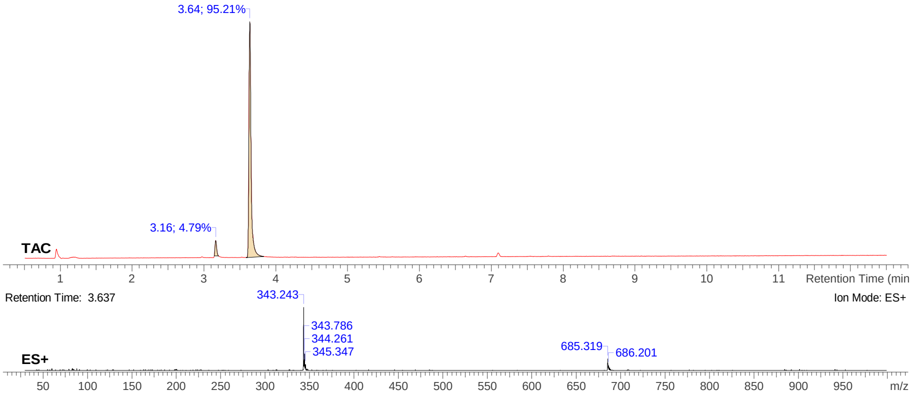

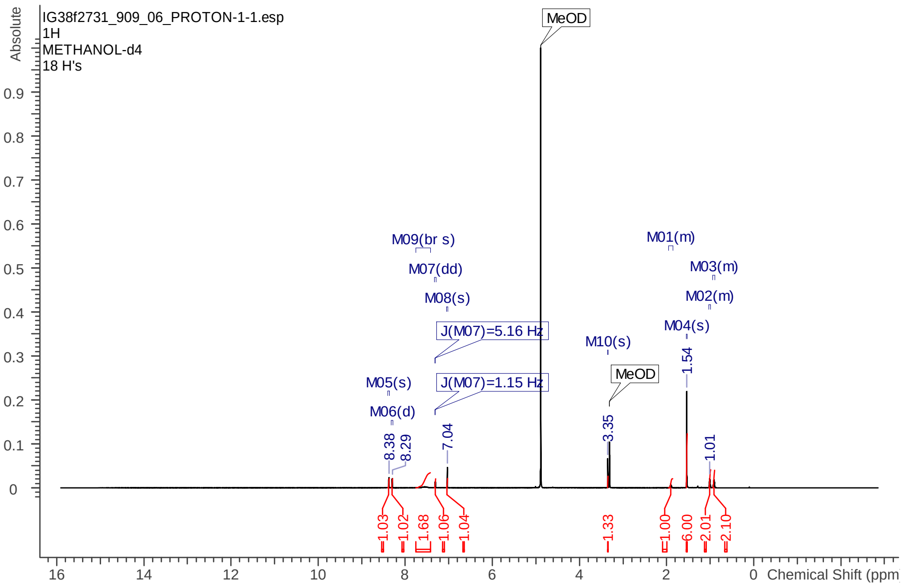

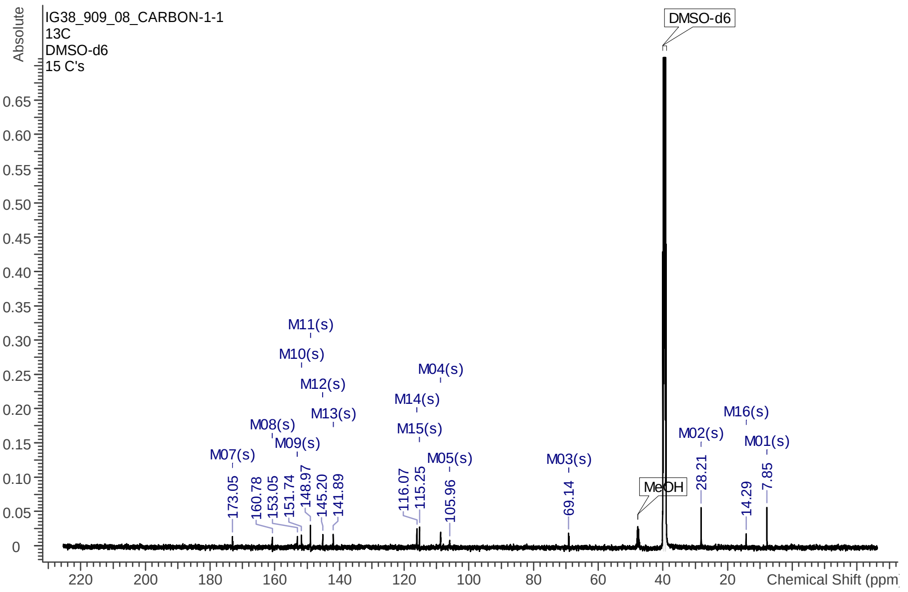

N-(4-(2,2-dimethyl-4-oxo-1,2,3,4-tetrahydrothieno[3,2-*d*]pyrimidin-6-yl)pyridin-2-yl)-cyclobutanecarboxamide (**41**)

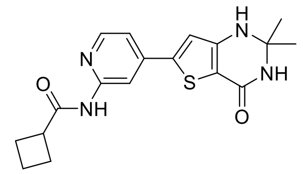

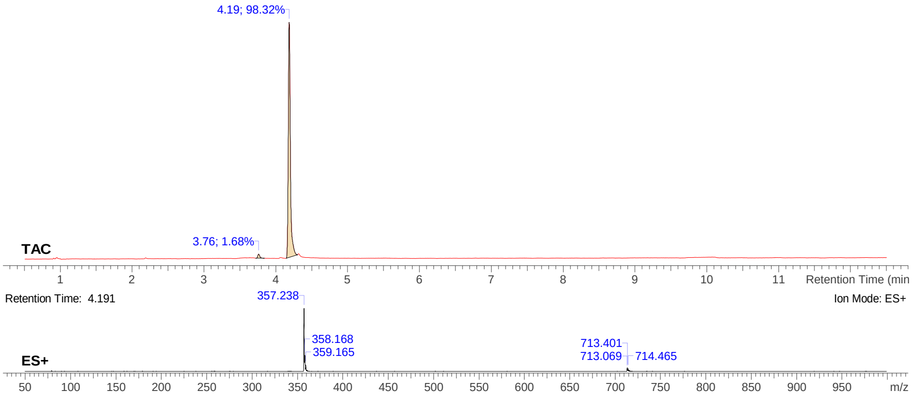
4.19; 98.32%
3.76; 1.68%
TAC
Retention Time (min
Retention Time: 4.191
Ion Mode: ES+
357.238
358.168
359.165
713.401
713.069
714.465
ES+
m/z

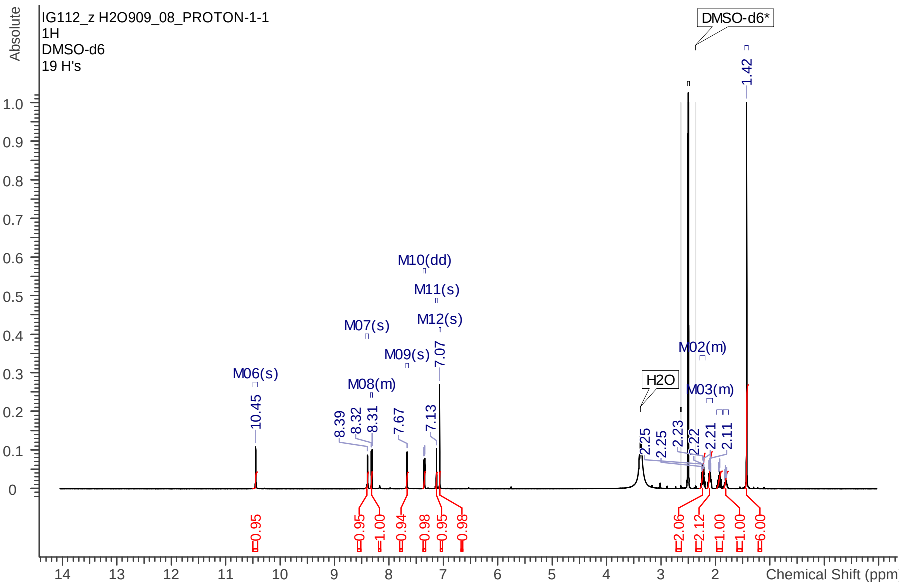
IG112_z H2O909_08_PROTON-1-1
1H
DMSO-d6
19 H's
Absolute
DMSO-d6*
H2O
M06(s)
M07(s)
M08(m)
M09(s)
M10(dd)
M11(s)
M12(s)
M02(m)
M03(m)
10.45
8.39
8.32
8.31
7.67
7.13
7.07
2.25
2.25
2.23
2.22
2.21
2.11
1.42
0.95
0.95
1.00
0.94
0.98
0.95
0.98
2.06
2.12
1.00
1.00
6.00
Chemical Shift (ppm

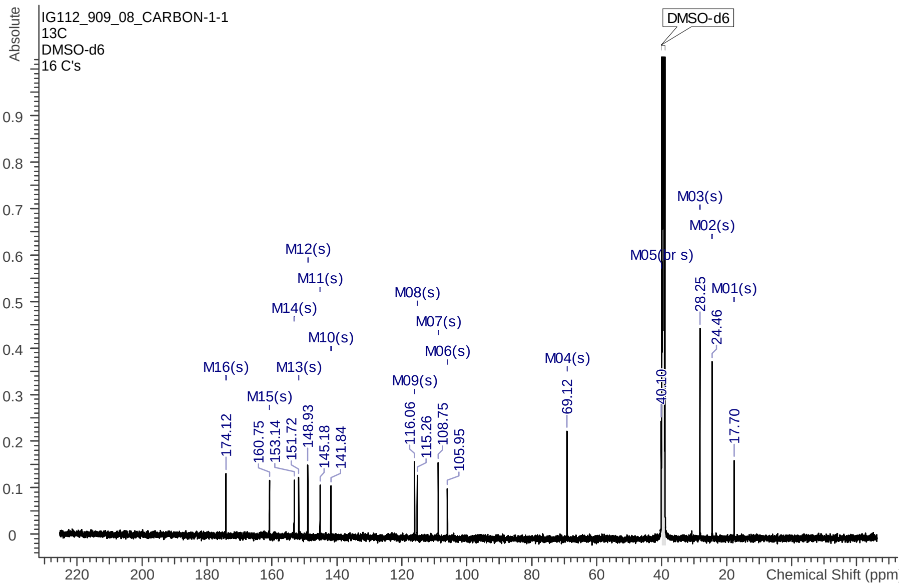

N-(4-(2,2-dimethyl-4-oxo-1,2,3,4-tetrahydrothieno[3,2-*d*]pyrimidin-6-yl)pyridin-2-yl)-cyclohexanecarboxamide (**42**)

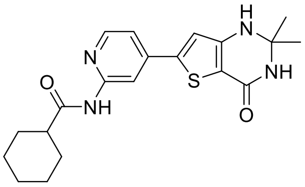

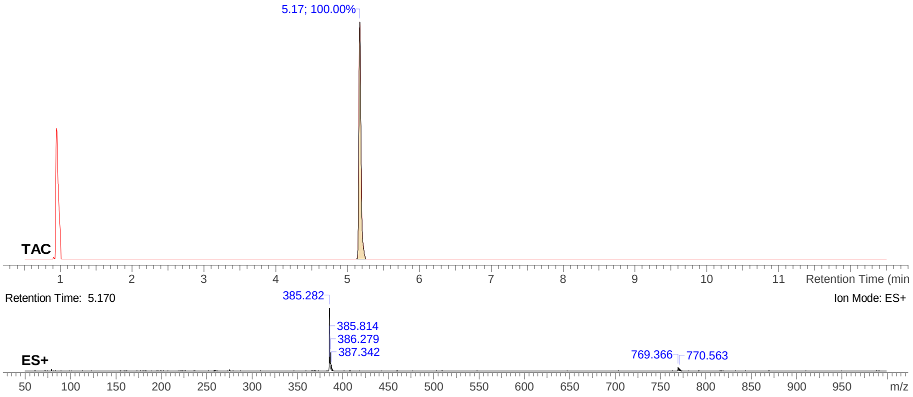

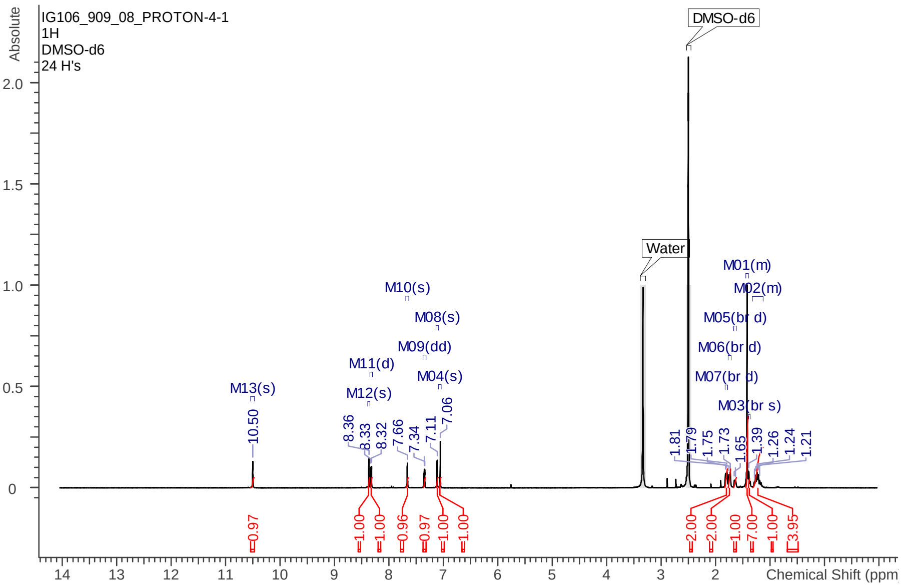

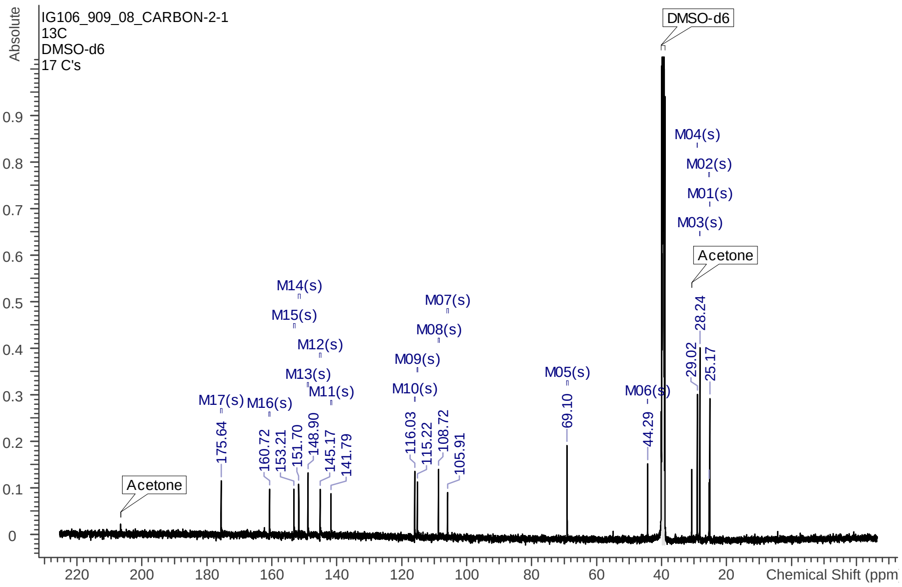

N-(4-(2,2-dimethyl-4-oxo-1,2,3,4-tetrahydrothieno[3,2-*d*]pyrimidin-6-yl)pyridin-2-yl)benzamide (**43**)

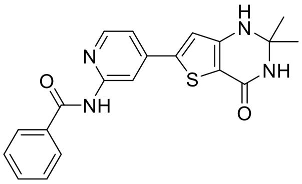
H
N
NH
O
S
N
O
NH

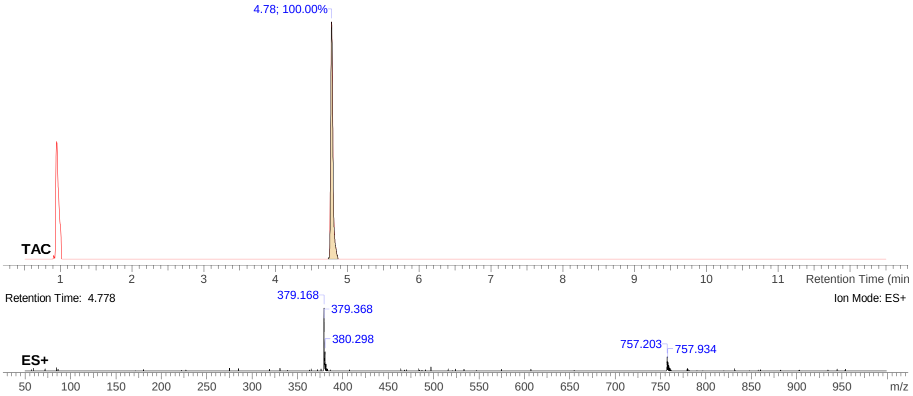
4.78; 100.00%
TAC
Retention Time (min
Retention Time: 4.778
Ion Mode: ES+
379.168
379.368
380.298
757.203
757.934
ES+
m/z

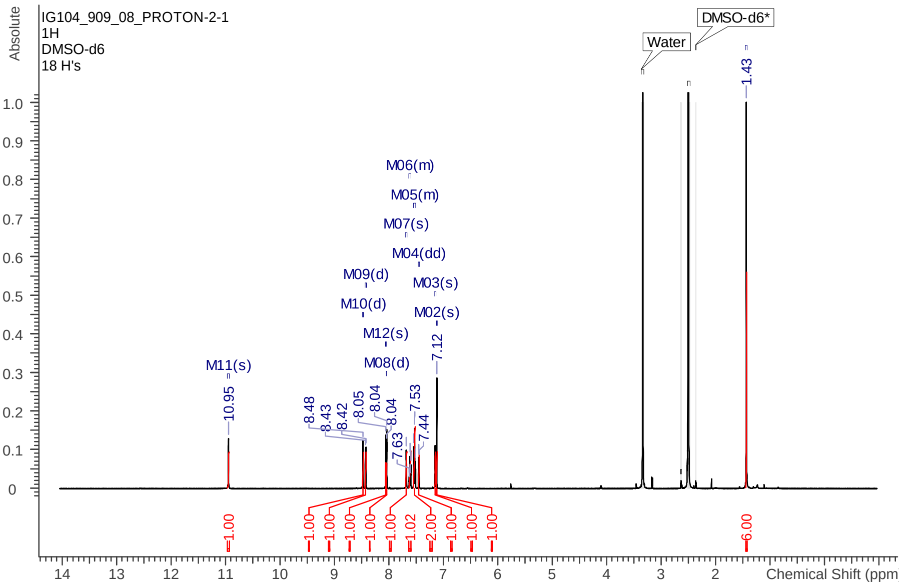
IG104_909_08_PROTON-2-1
1H
DMSO-d6
18 H's
Absolute
Water
DMSO-d6*
M11(s)
M09(d)
M10(d)
M12(s)
M08(d)
M06(m)
M05(m)
M07(s)
M04(dd)
M03(s)
M02(s)
10.95
8.48
8.43
8.42
8.05
8.04
8.04
7.63
7.53
7.44
7.12
1.43
Chemical Shift (ppm

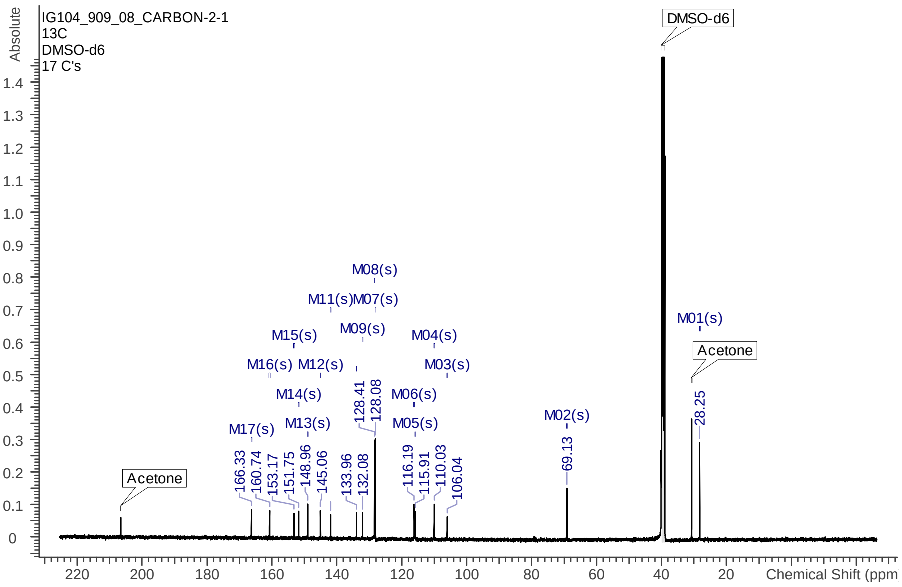

N-(4-(2,2-dimethyl-4-oxo-1,2,3,4-tetrahydrothieno[3,2-*d*]pyrimidin-6-yl)pyridin-2-yl)-methanesulfonamide (**44**)

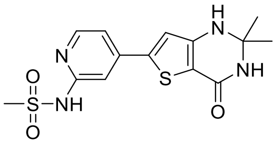

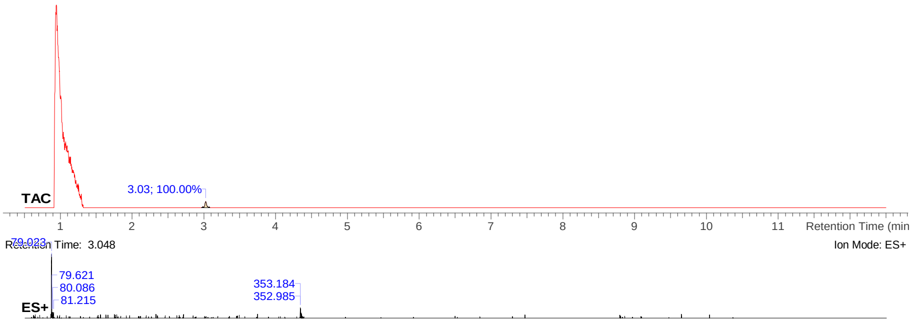

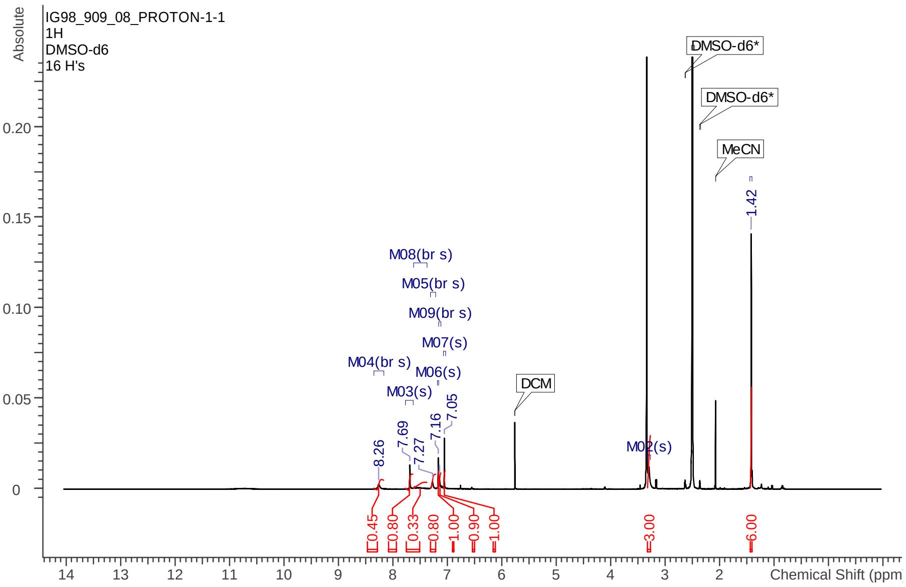

N-(4-(2,2-dimethyl-4-oxo-1,2,3,4-tetrahydrothieno[3,2-*d*]pyrimidin-6-yl)pyridin-2-yl)-ethanesulfonamide (**45**)

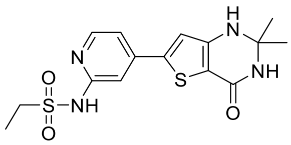

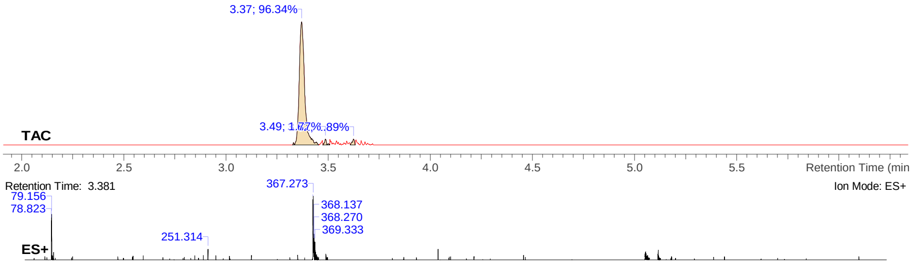

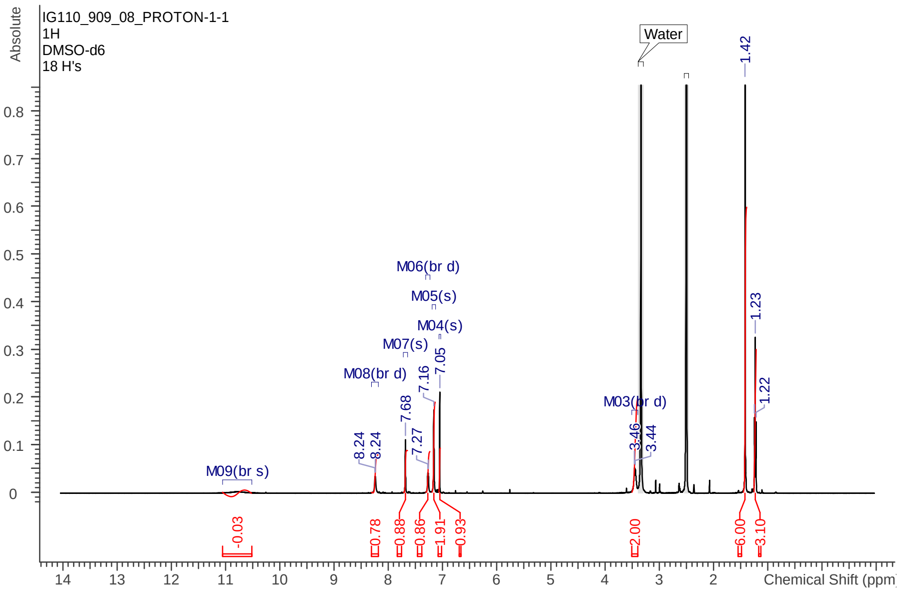

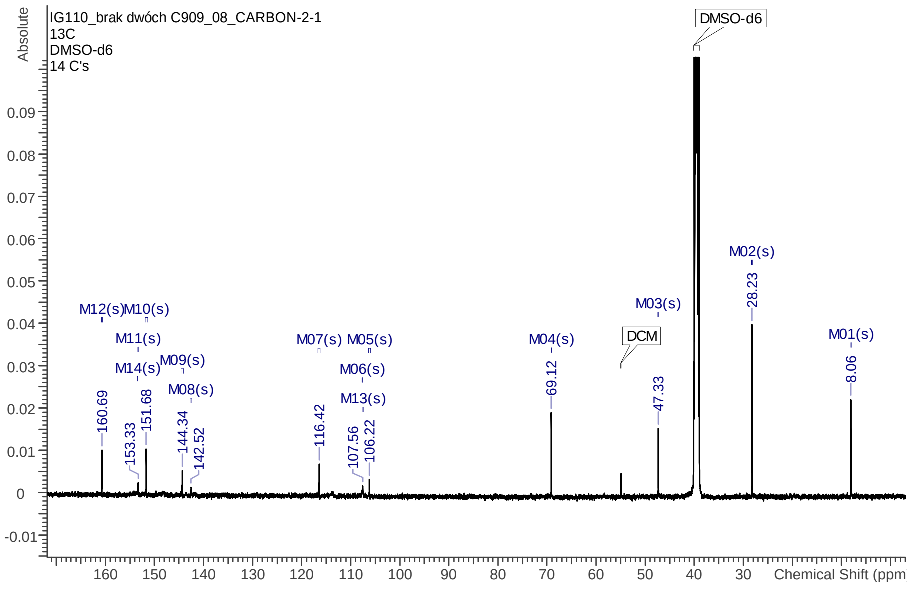

6-(2-((Cyclopropylmethyl)amino)pyridin-4-yl)-2,2-dimethyl-2,3-dihydrothieno[3,2-d]pyrimidin-4(1*H*)-one (**46**)

Commented [TW1]: dodać LCMS jak przyjdzie

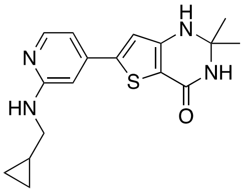
H
N
NH
O
S
N
HN

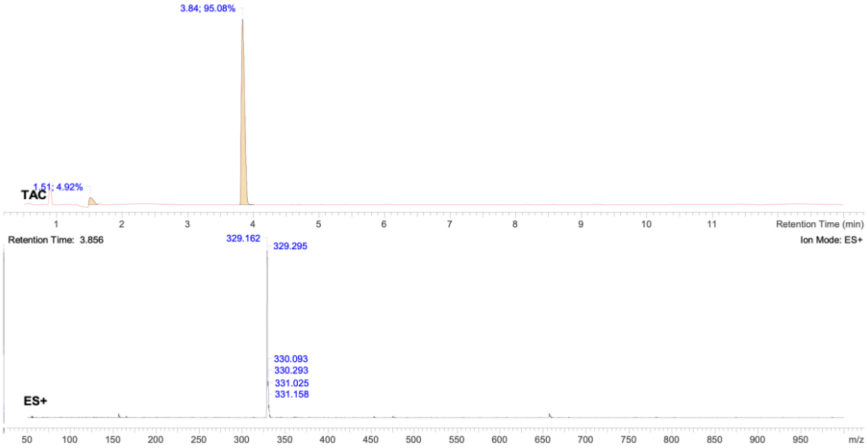
3.84; 95.08%
1.51; 4.92%
TAC
Retention Time (min)
Retention Time: 3.856
Ion Mode: ES+
329.162
329.295
330.093
330.293
331.025
331.158
ES+
m/z

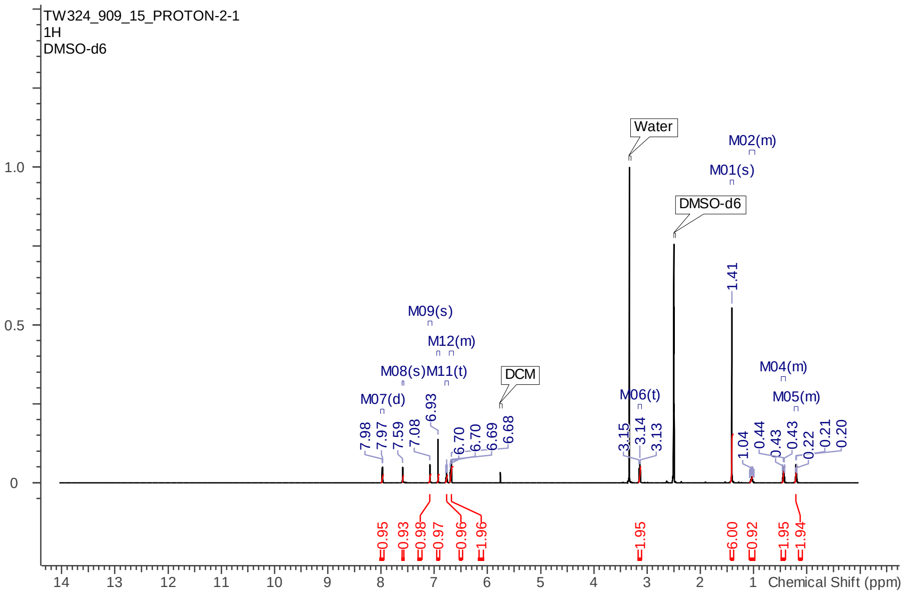
TW324_909_15_PROTON-2-1
1H
DMSO-d6
Water
DMSO-d6
DCM
M01(s)
M02(m)
M04(m)
M05(m)
M06(t)
M07(d)
M08(s)
M09(s)
M11(t)
M12(m)
Chemical Shift (ppm)

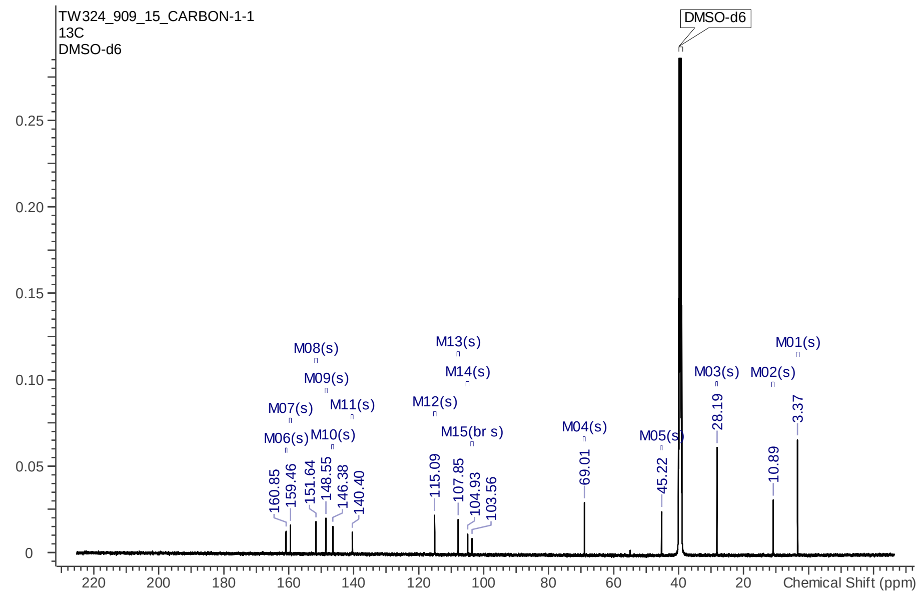

5-(2-acetamidopyridin-4-yl)-3-ureidothiophene-2-carboxamide (**47**)

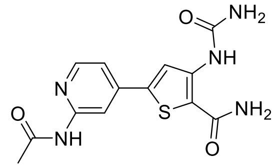

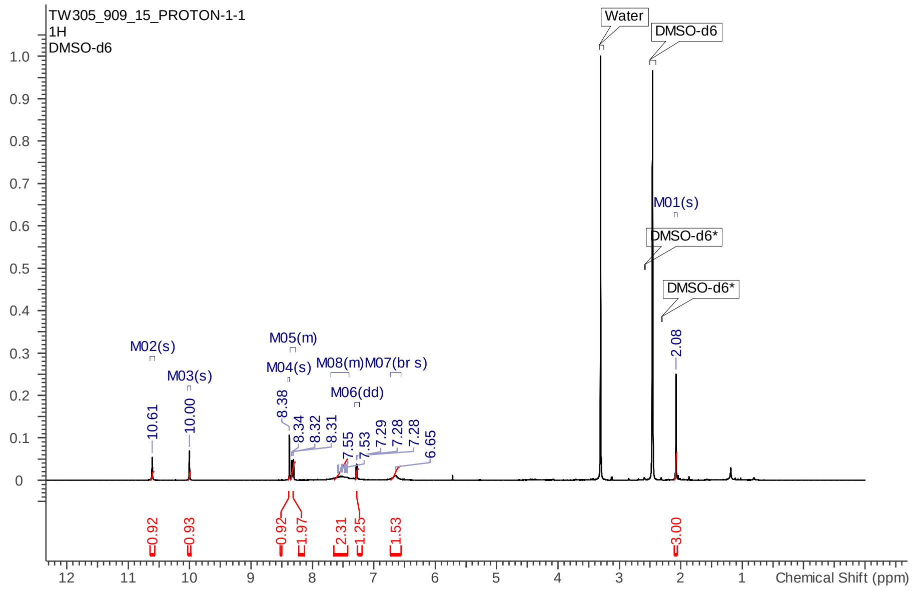
TW305_909_15_PROTON-1-1
1H
DMSO-d6
Water
DMSO-d6
M01(s)
DMSO-d6*
DMSO-d6*
M02(s)
M03(s)
M05(m)
M04(s)
M08(m)
M07(br s)
M06(dd)
10.61
10.00
8.38
8.34
8.32
8.31
7.55
7.53
7.29
7.28
7.28
6.65
2.08
0.92
0.93
0.92
1.97
2.31
1.25
1.53
3.00
Chemical Shift (ppm)

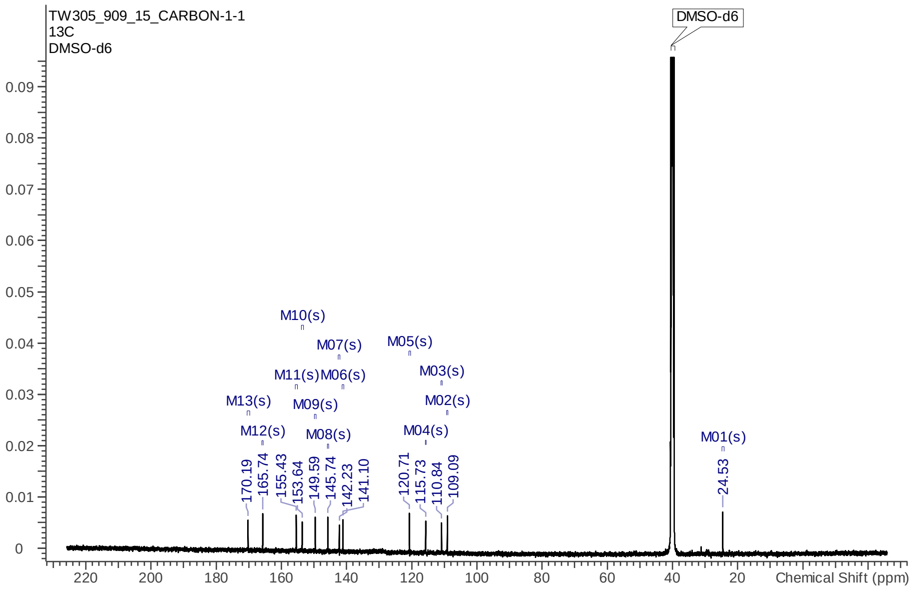
TW305_909_15_CARBON-1-1
13C
DMSO-d6
DMSO-d6
M10(s)
M07(s)
M05(s)
M11(s)
M06(s)
M03(s)
M13(s)
M09(s)
M02(s)
M12(s)
M08(s)
M04(s)
M01(s)
170.19
165.74
155.43
153.64
149.59
145.74
142.23
141.10
120.71
115.73
110.84
109.09
24.53
Chemical Shift (ppm)

5-(2-isobutyramidopyridin-4-yl)-3-ureidothiophene-2-carboxamide (**48**)

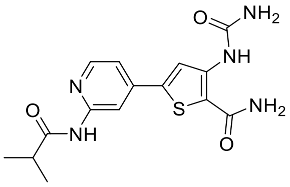

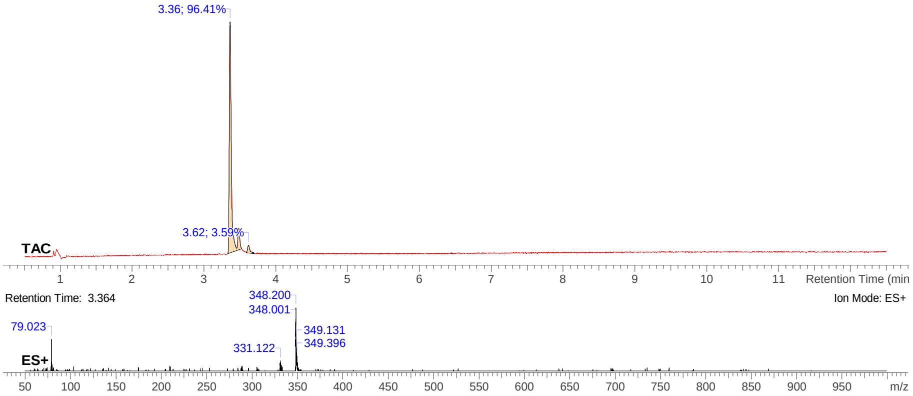

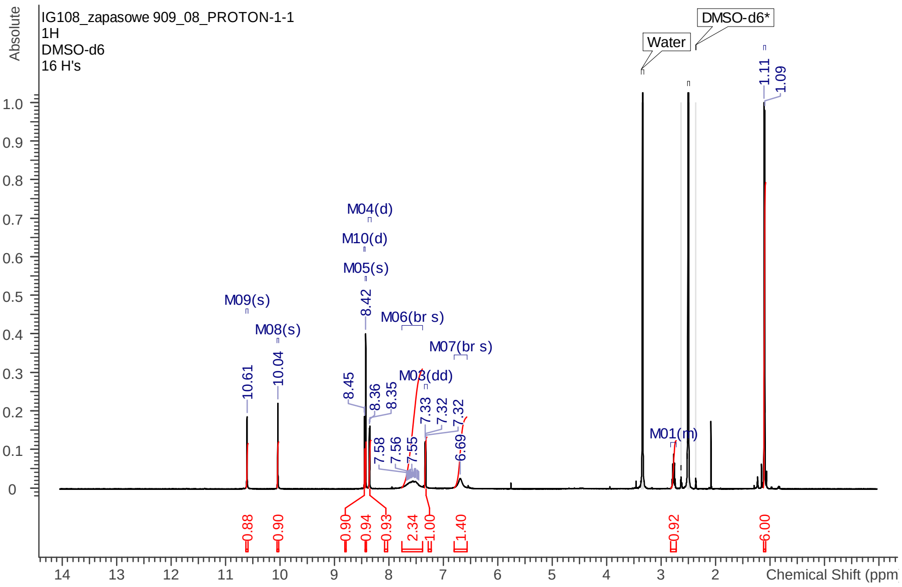

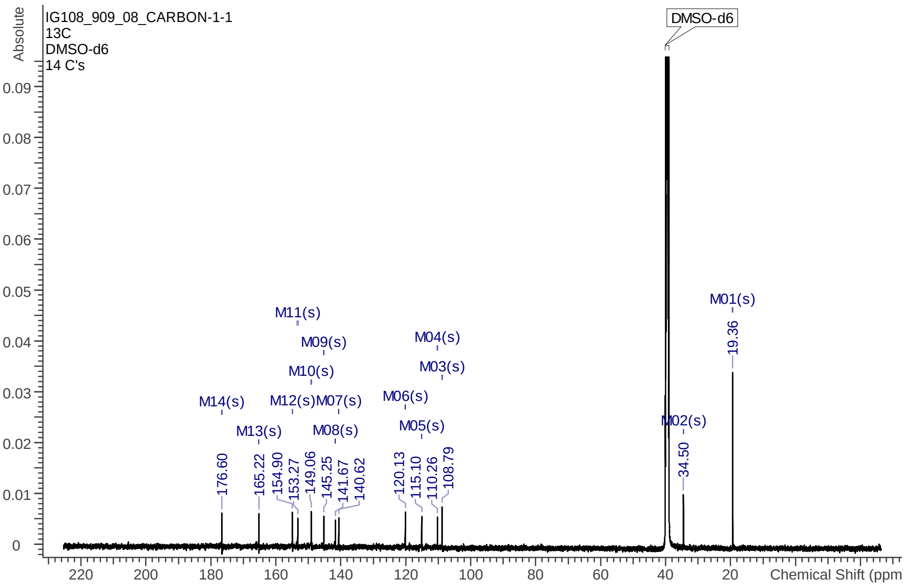

5-(2-(cyclopropanecarboxamido)pyridin-4-yl)-3-ureidothiophene-2-carboxamide (**49**)

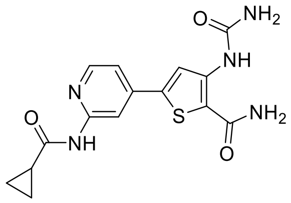

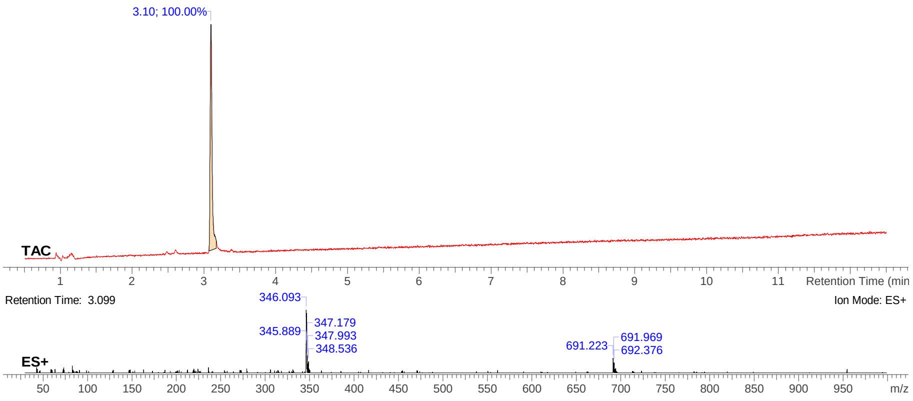

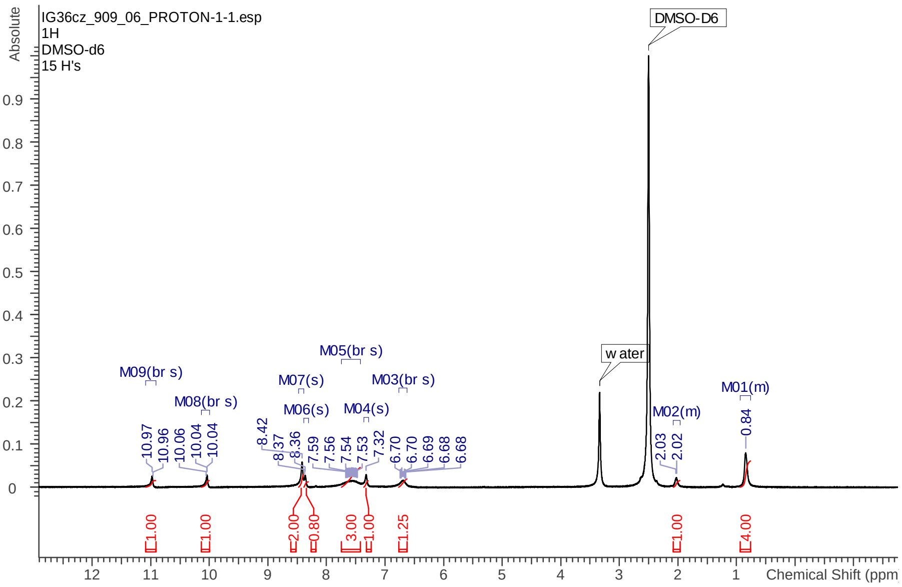

IG36cz_909_06_PROTON-1-1.esp
1H
DMSO-d6
15 H's
Absolute
DMSO-D6
water
M09(br s)
M08(br s)
M07(s)
M06(s)
M05(br s)
M04(s)
M03(br s)
M02(m)
M01(m)
10.97
10.96
10.06
10.04
10.04
8.42
8.37
8.36
7.59
7.56
7.54
7.53
7.32
6.70
6.70
6.69
6.68
6.68
2.03
2.02
0.84
1.00
1.00
2.00
0.80
3.00
1.00
1.25
1.00
4.00
Chemical Shift (ppm)

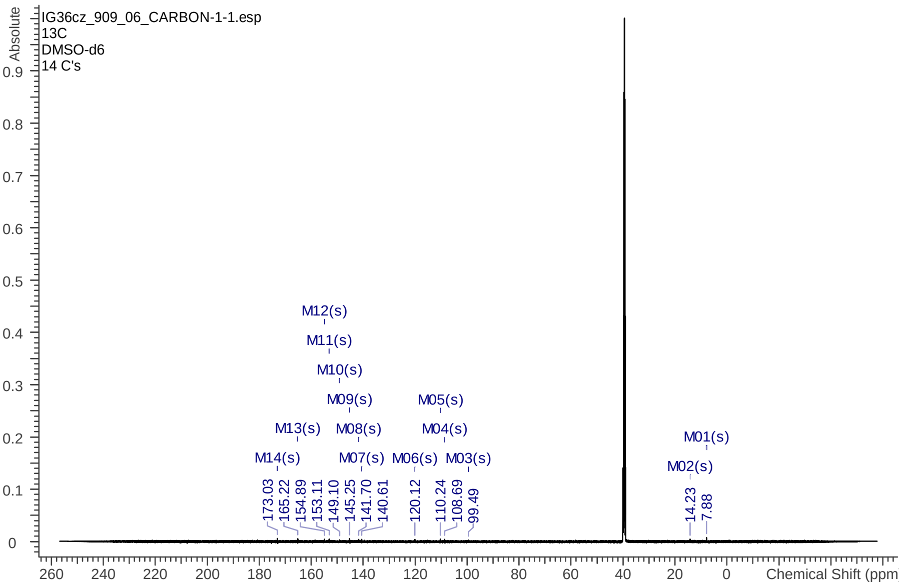

IG36cz_909_06_CARBON-1-1.esp
13C
DMSO-d6
14 C's
Absolute
M12(s)
M11(s)
M10(s)
M09(s)
M05(s)
M13(s)
M08(s)
M04(s)
M14(s)
M07(s)
M06(s)
M03(s)
M01(s)
M02(s)
173.03
165.22
154.89
153.11
149.10
145.25
141.70
140.61
120.12
110.24
108.69
99.49
14.23
7.88
Chemical Shift (ppm)

5-(2-(cyclobutanecarboxamido)pyridin-4-yl)-3-ureidothiophene-2-carboxamide (**50**)

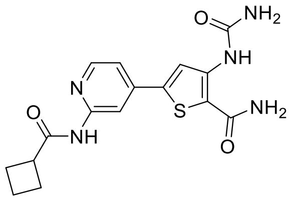

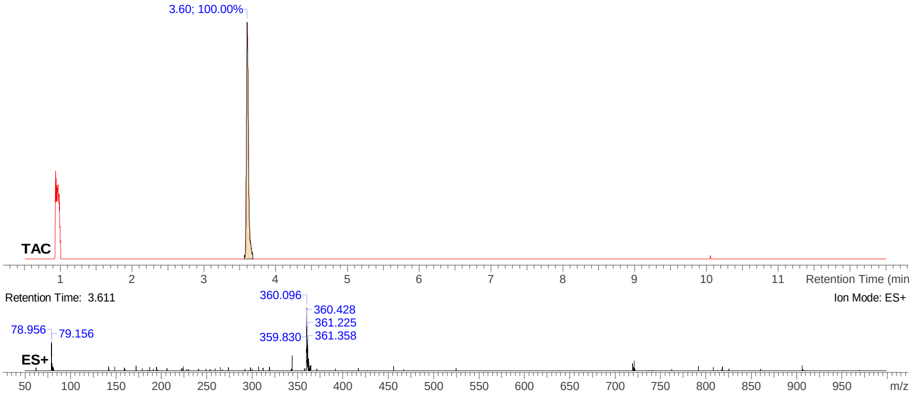

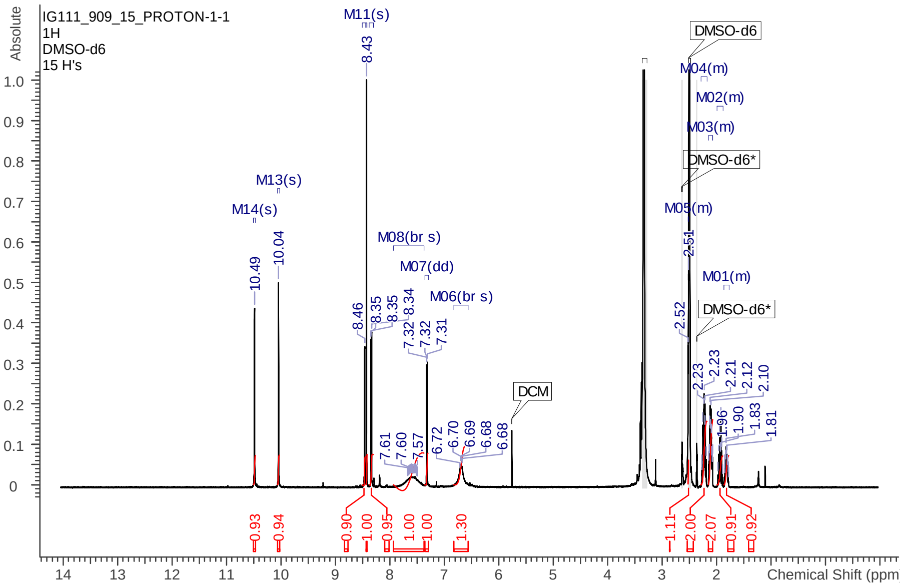

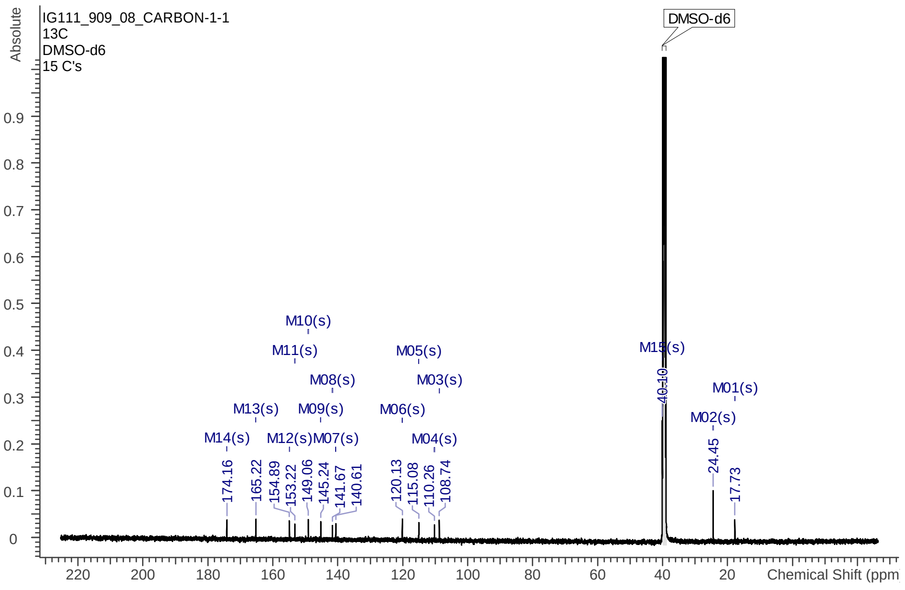

5-(2-(cyclohexanecarboxamido)pyridin-4-yl)-3-ureidothiophene-2-carboxamide (**51**)

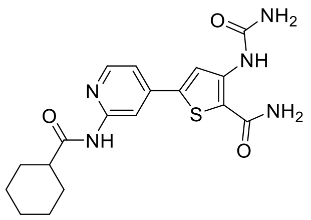

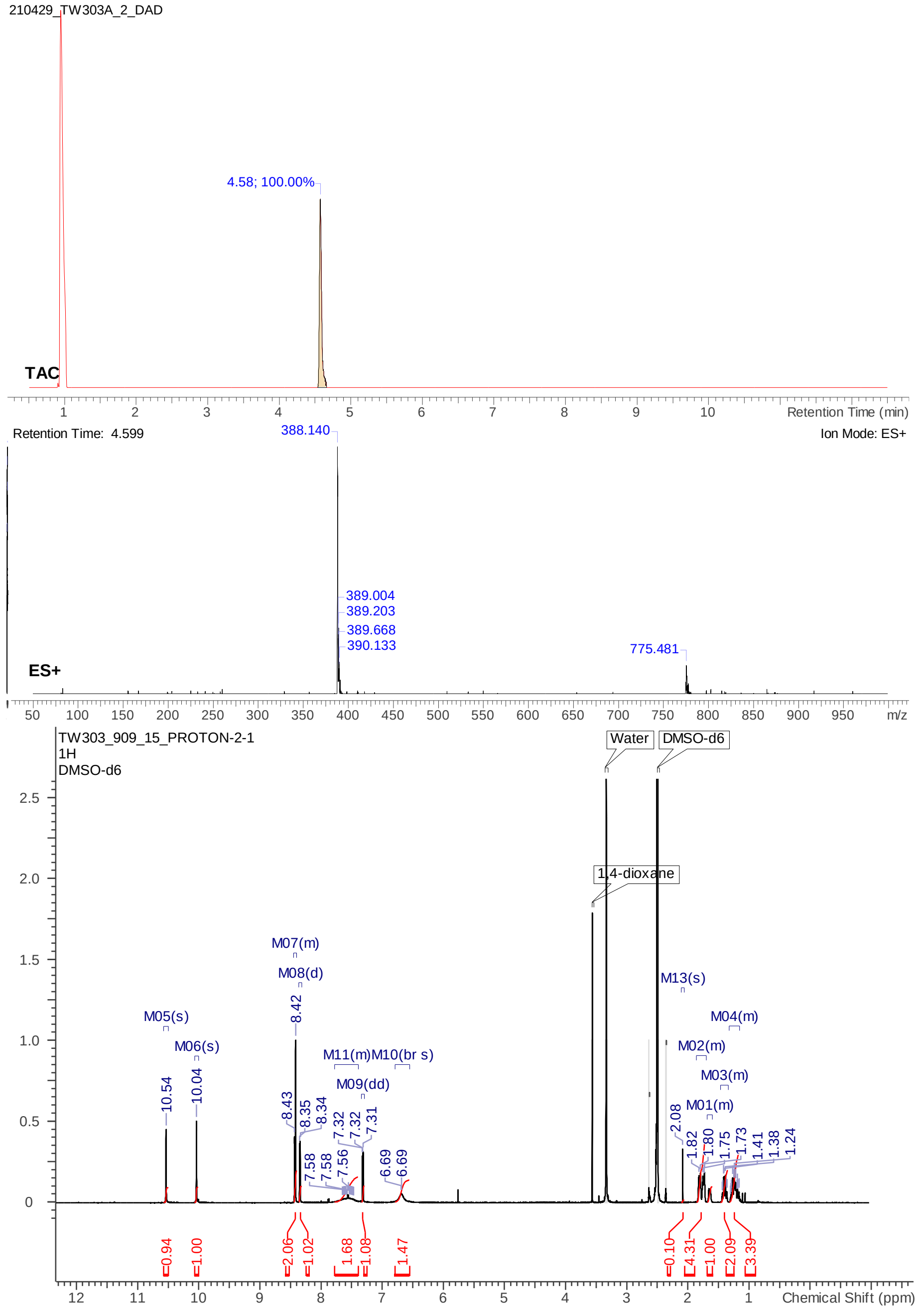

210429_TW303A_2_DAD
4.58; 100.00%
TAC
Retention Time (min)
Retention Time: 4.599
Ion Mode: ES+
388.140
389.004
389.203
389.668
390.133
775.481
ES+
m/z
TW303_909_15_PROTON-2-1
1H
DMSO-d6
Water
DMSO-d6
1,4-dioxane
M05(s)
M06(s)
M07(m)
M08(d)
M11(m)
M10(br s)
M09(dd)
M13(s)
M04(m)
M02(m)
M03(m)
M01(m)
Chemical Shift (ppm)

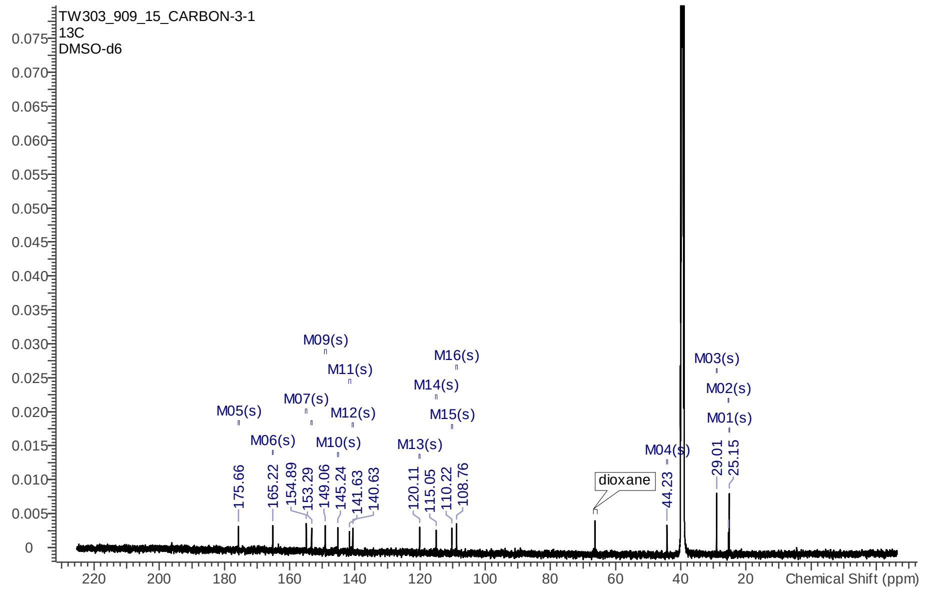

5-(2-benzamidopyridin-4-yl)-3-ureidothiophene-2-carboxamide (**52**)

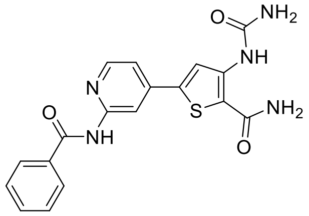

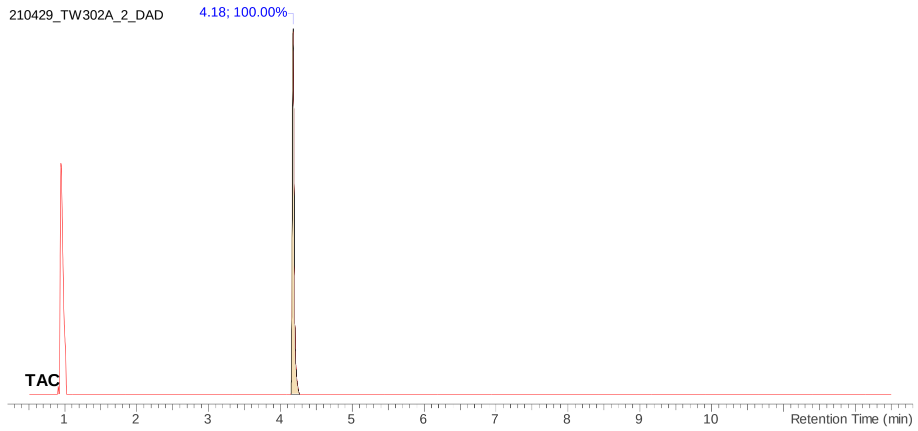

210429_TW302A_2_DAD
4.18; 100.00%
TAC
Retention Time (min)

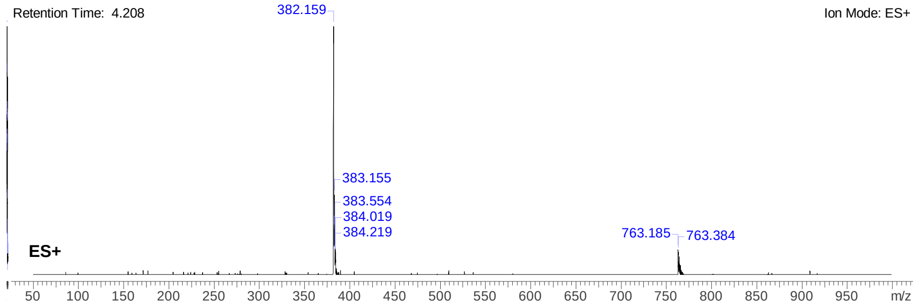

Retention Time: 4.208
Ion Mode: ES+
382.159
383.155
383.554
384.019
384.219
763.185
763.384
ES+
m/z

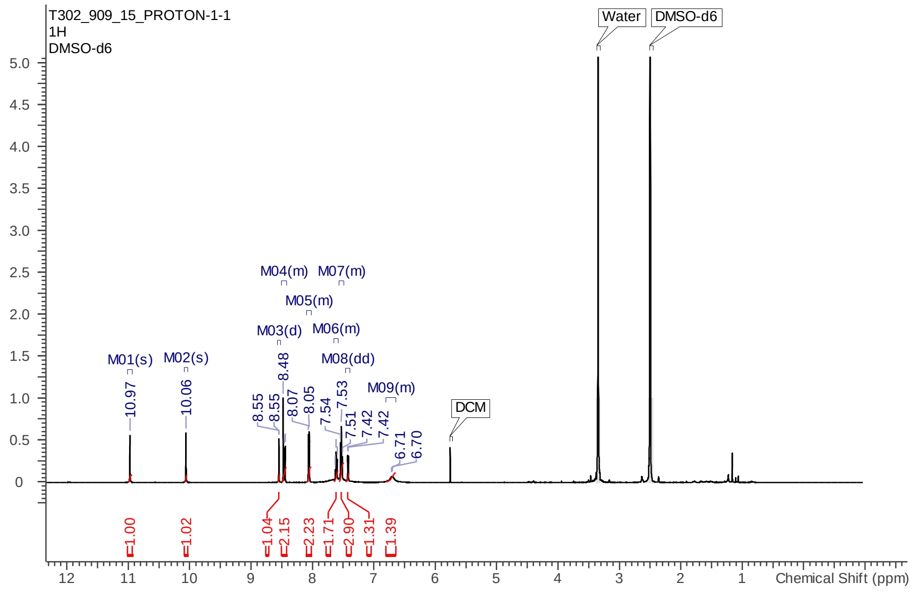

T302_909_15_PROTON-1-1
1H
DMSO-d6
Water
DMSO-d6
DCM
M01(s)
M02(s)
M03(d)
M04(m)
M05(m)
M06(m)
M07(m)
M08(dd)
M09(m)
10.97
10.06
8.55
8.55
8.48
8.07
8.05
7.54
7.53
7.51
7.42
7.42
6.71
6.70
1.00
1.02
1.04
2.15
2.23
1.71
2.90
1.31
1.39
Chemical Shift (ppm)

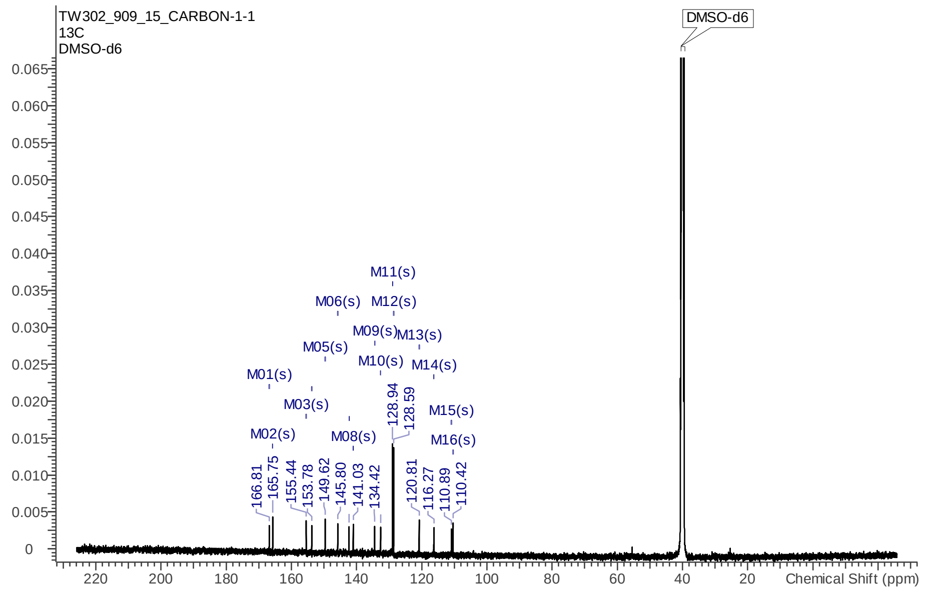
TW302_909_15_CARBON-1-1
13C
DMSO-d6
DMSO-d6
M01(s)
M02(s)
M03(s)
M05(s)
M06(s)
M08(s)
M09(s)
M10(s)
M11(s)
M12(s)
M13(s)
M14(s)
M15(s)
M16(s)
166.81
165.75
155.44
153.78
149.62
145.80
141.03
134.42
128.94
128.59
120.81
116.27
110.89
110.42
Chemical Shift (ppm)

N-(3-(2,2-dimethyl-4-oxo-1,2,3,4-tetrahydrothieno[3,2-*d*]pyrimidin-6-yl)phenyl)cyclopropanecarboxamide (**53**)

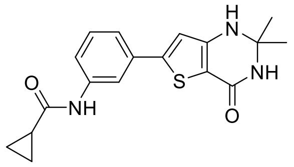

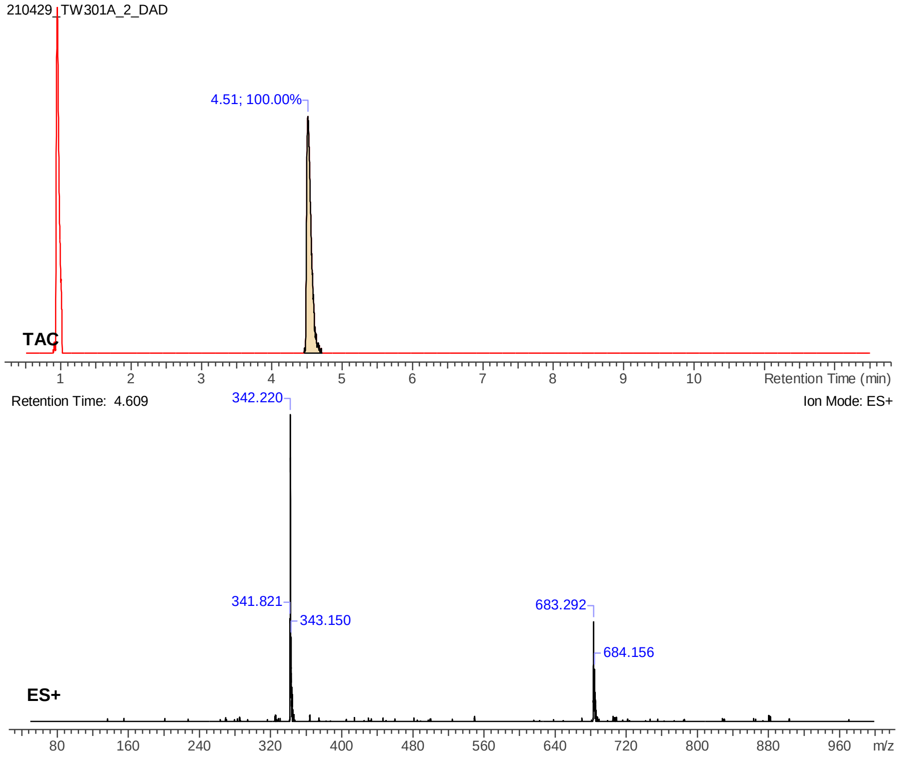

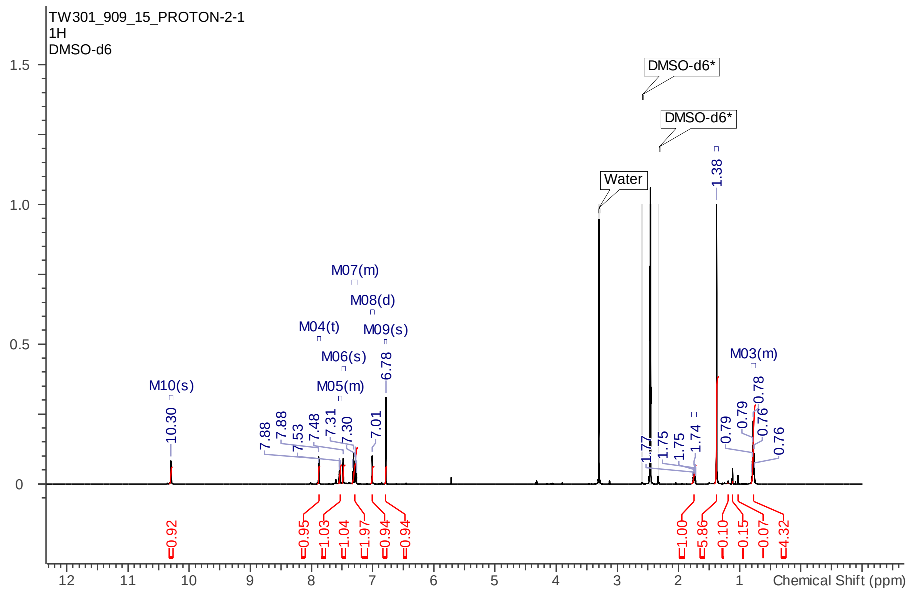
TW301_909_15_PROTON-2-1
1H
DMSO-d6
Chemical Shift (ppm)

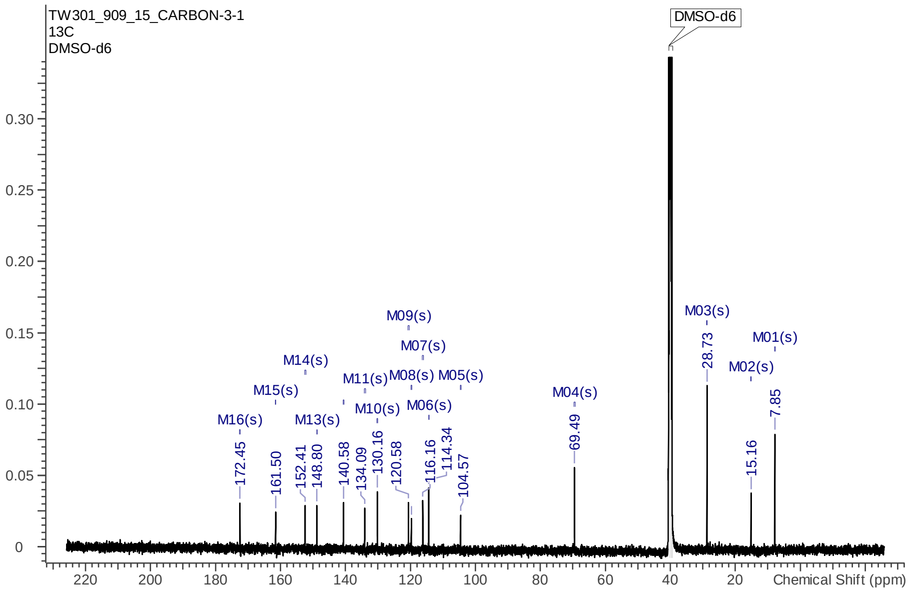
TW301_909_15_CARBON-3-1
13C
DMSO-d6
Chemical Shift (ppm)

N-(5-(2,2-dimethyl-4-oxo-1,2,3,4-tetrahydrothieno[3,2-*d*]pyrimidin-6-yl)-2-fluorophenyl)-cyclopropanecarboxamide (**54**)

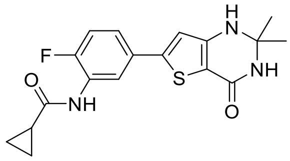

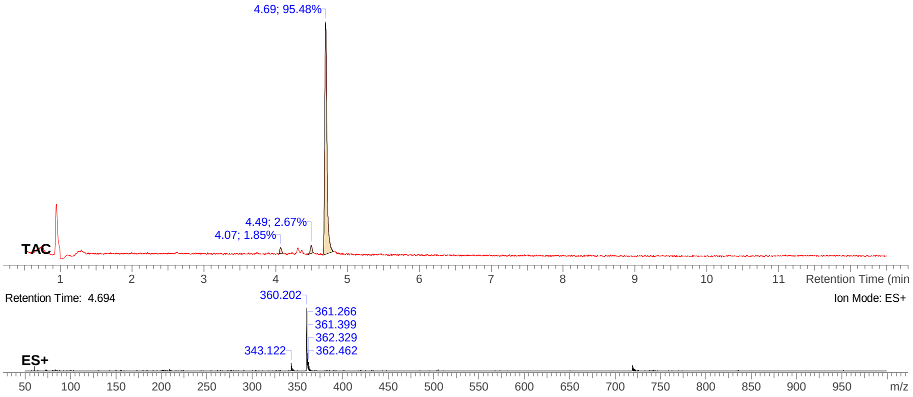

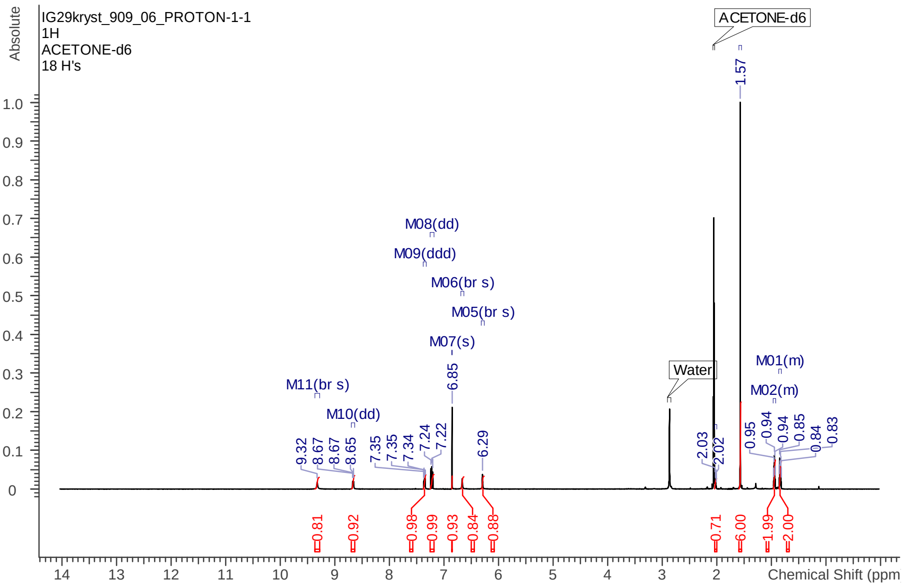

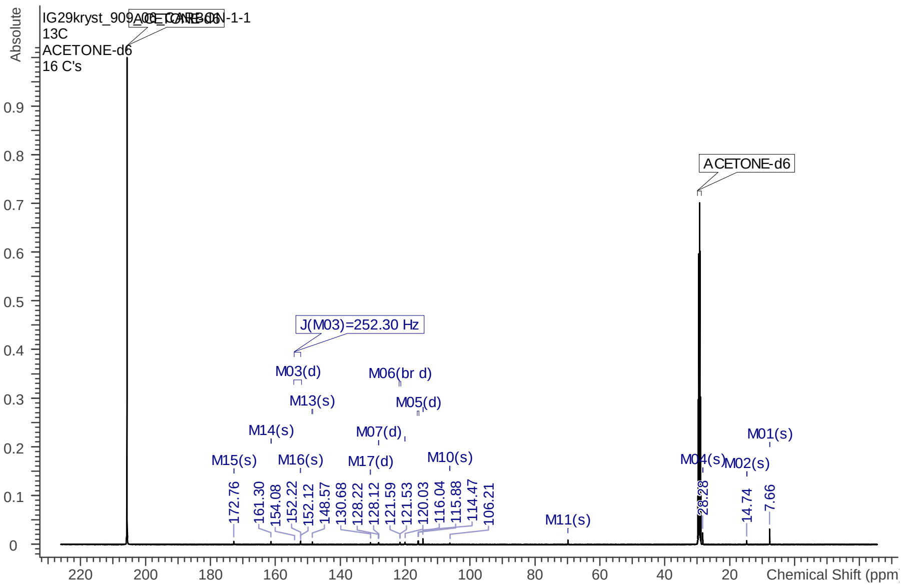

N-(3-(2,2-dimethyl-4-oxo-1,2,3,4-tetrahydrothieno[3,2-*d*]pyrimidin-6-yl)phenyl)ethanesulfonamide (**55**)

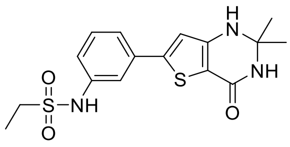

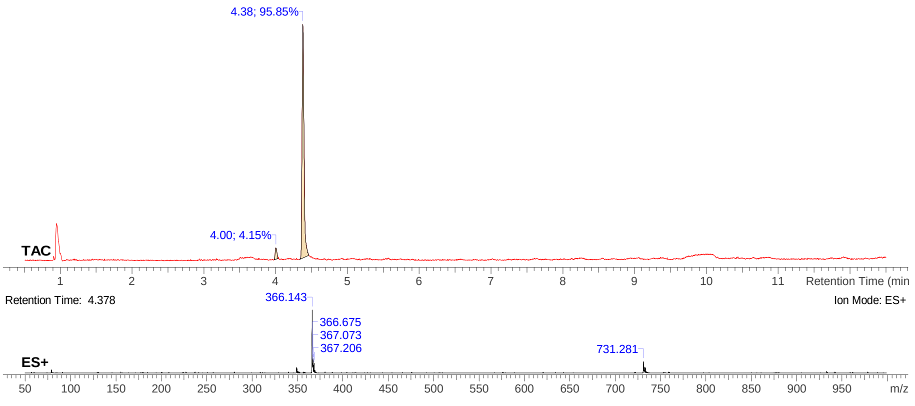

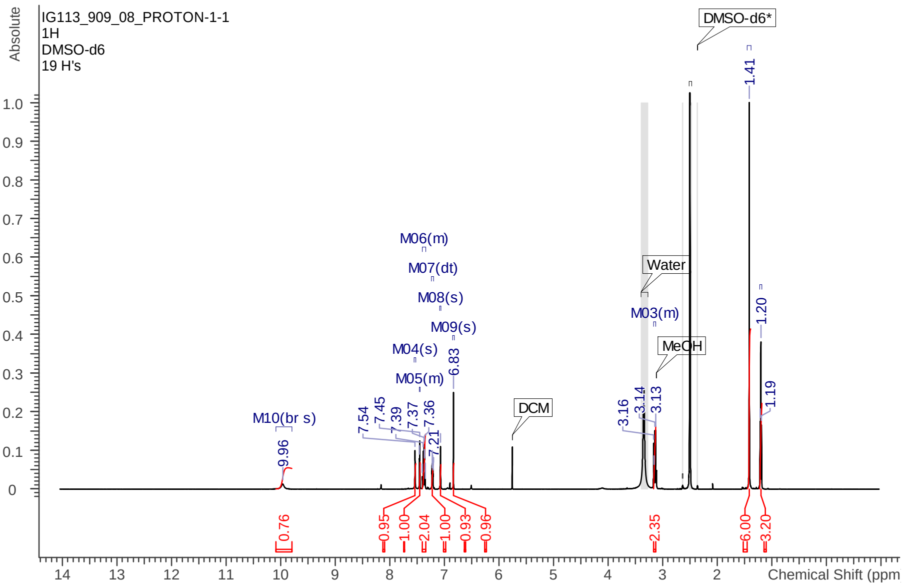
IG113_909_08_PROTON-1-1
1H
DMSO-d6
19 H's
Absolute
DMSO-d6*
Water
MeOH
DCM
M10(br s)
M06(m)
M07(dt)
M08(s)
M09(s)
M04(s)
M05(m)
M03(m)
9.96
7.54
7.45
7.39
7.37
7.36
7.21
6.83
3.16
3.14
3.13
1.41
1.20
1.19
0.76
0.95
1.00
2.04
1.00
0.93
0.96
2.35
6.00
3.20
Chemical Shift (ppm)

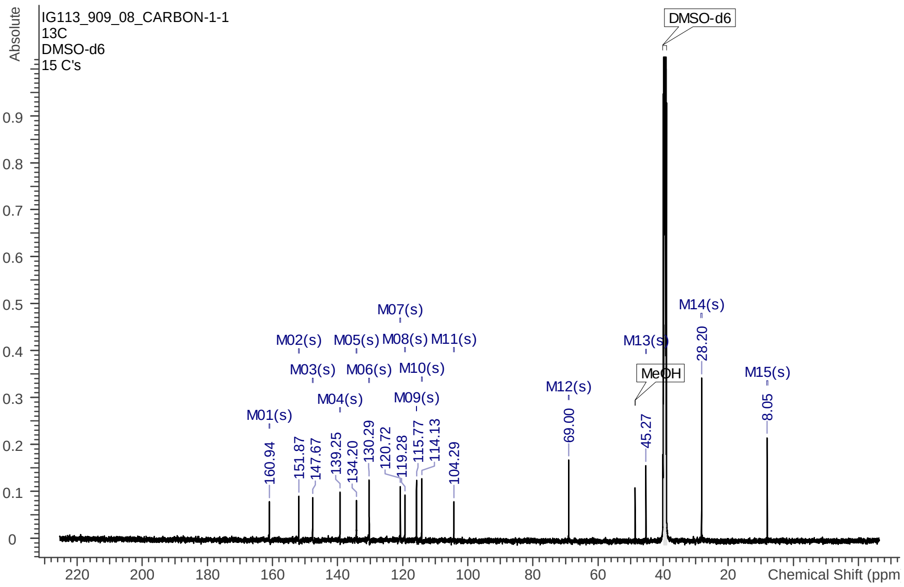
IG113_909_08_CARBON-1-1
13C
DMSO-d6
15 C's
Absolute
DMSO-d6
MeOH
M01(s)
M02(s)
M03(s)
M04(s)
M05(s)
M06(s)
M07(s)
M08(s)
M09(s)
M10(s)
M11(s)
M12(s)
M13(s)
M14(s)
M15(s)
160.94
151.87
147.67
139.25
134.20
130.29
120.72
119.28
115.77
114.13
104.29
69.00
45.27
28.20
8.05
Chemical Shift (ppm)

N-(3-(2,2-dimethyl-4-oxo-1,2,3,4-tetrahydrothieno[3,2-*d*]pyrimidin-6-yl)phenyl)cyclohexanesulfonamide (**56**)

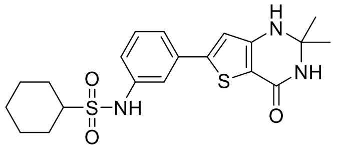

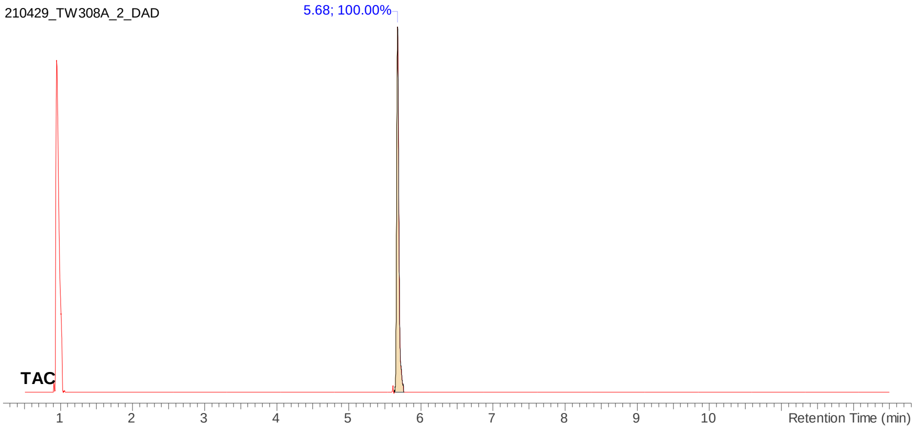

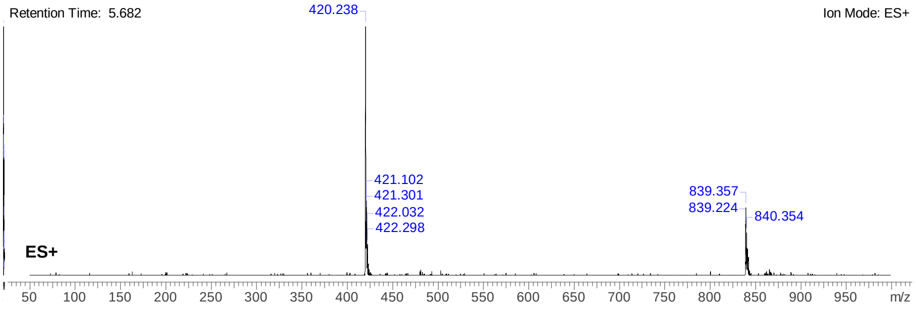

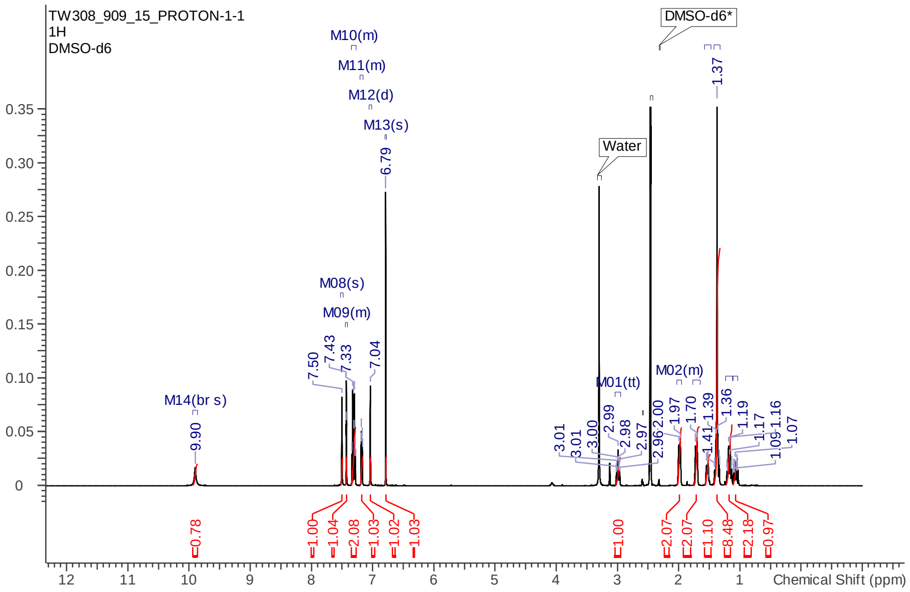
TW308_909_15_PROTON-1-1
1H
DMSO-d6
DMSO-d6*
Water
M01(tt)
M02(m)
M08(s)
M09(m)
M10(m)
M11(m)
M12(d)
M13(s)
M14(br s)
9.90
7.50
7.43
7.33
7.04
6.79
3.01
3.01
3.00
2.99
2.98
2.97
2.96
2.00
1.97
1.70
1.41
1.39
1.37
1.36
1.19
1.17
1.16
1.09
1.07
0.78
1.00
1.04
2.08
1.03
1.02
1.03
1.00
2.07
2.07
1.10
8.48
2.18
0.97
Chemical Shift (ppm)

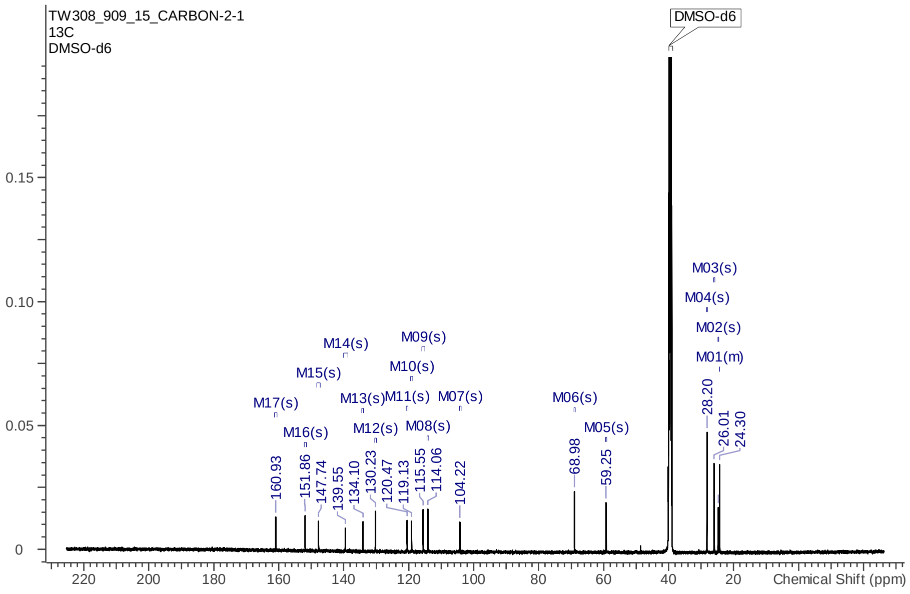
TW308_909_15_CARBON-2-1
13C
DMSO-d6
DMSO-d6
M01(m)
M02(s)
M03(s)
M04(s)
M05(s)
M06(s)
M07(s)
M08(s)
M09(s)
M10(s)
M11(s)
M12(s)
M13(s)
M14(s)
M15(s)
M16(s)
M17(s)
160.93
151.86
147.74
139.55
134.10
130.23
120.47
119.13
115.55
114.06
104.22
68.98
59.25
28.20
26.01
24.30
Chemical Shift (ppm)

2,2-dimethyl-6-(3-(piperidin-1-ylsulfonyl)phenyl)-2,3-dihydrothieno[3,2-*d*]pyrimidin-4(1*H*)-one (**57**)

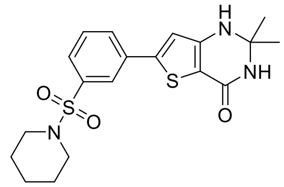

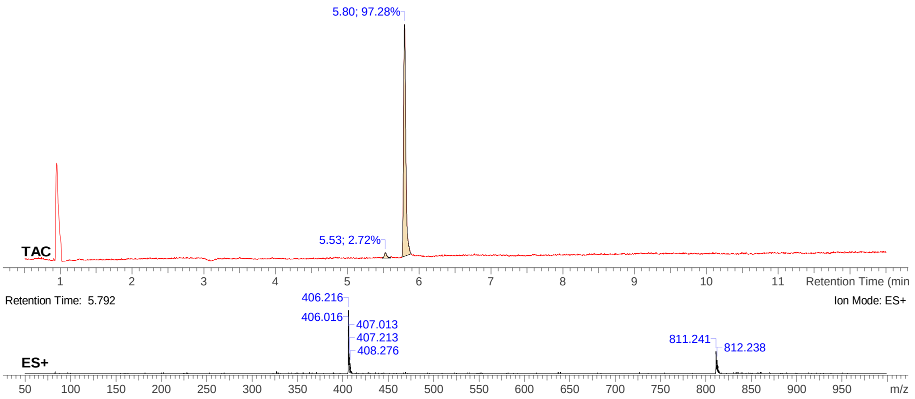

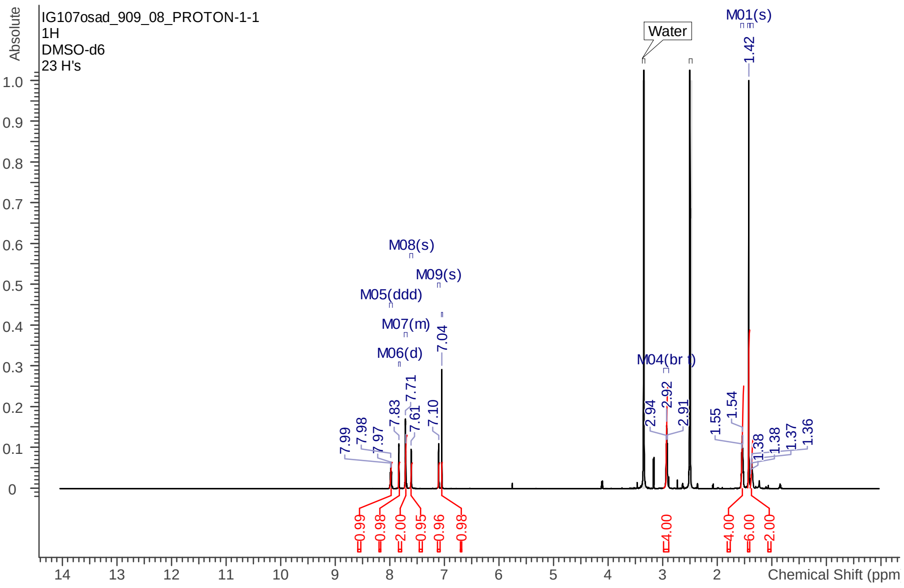

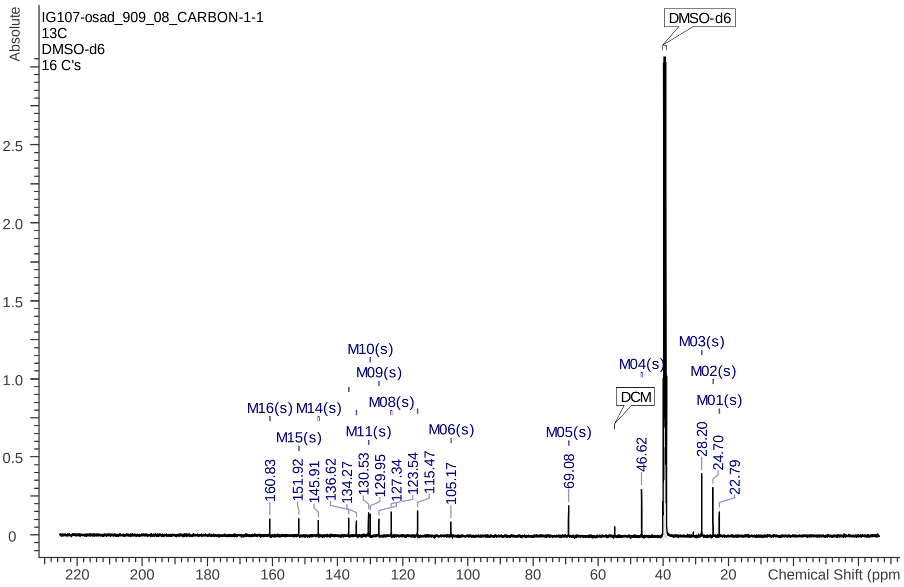

5-(3-(cyclopropanecarboxamido)phenyl)-3-ureidothiophene-2-carboxamide (**58**)

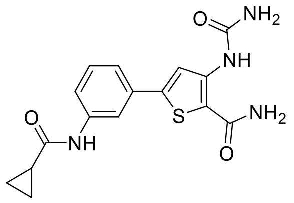

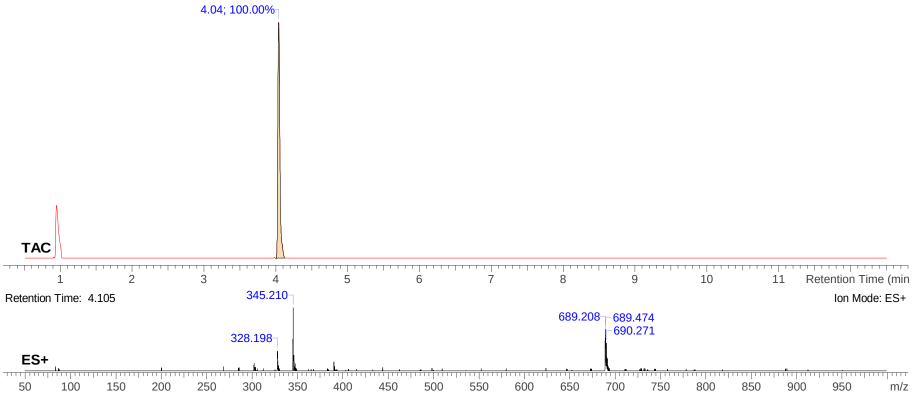

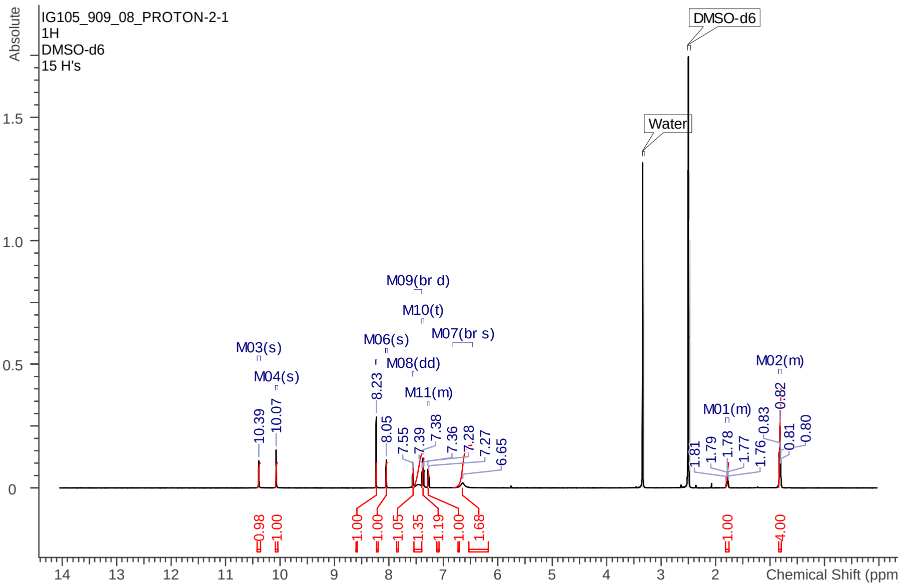
IG105_909_08_PROTON-2-1
1H
DMSO-d6
15 H's
Absolute
DMSO-d6
Water
M03(s)
M04(s)
M06(s)
M09(br d)
M10(t)
M07(br s)
M08(dd)
M11(m)
M01(m)
M02(m)
Chemical Shift (ppm

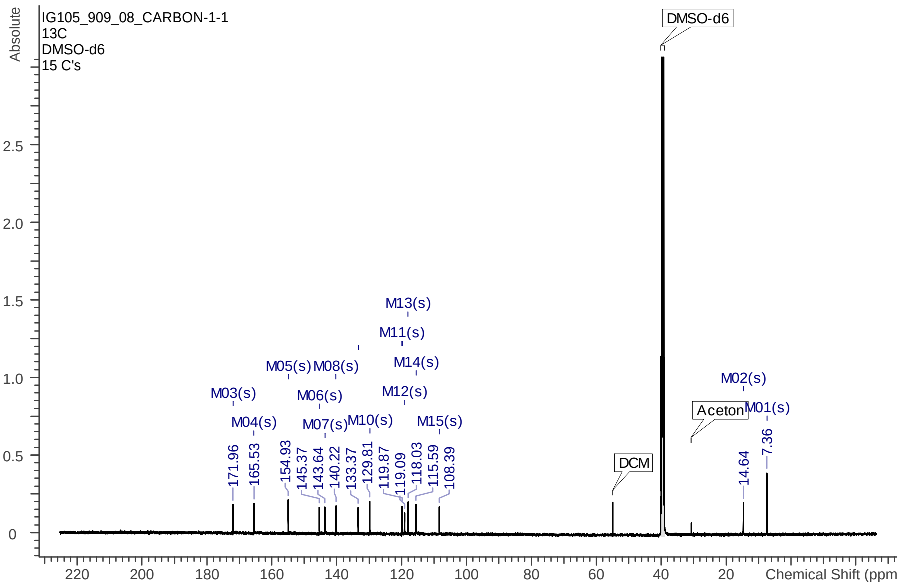
IG105_909_08_CARBON-1-1
13C
DMSO-d6
15 C's
Absolute
DMSO-d6
DCM
Aceton
M03(s)
M04(s)
M05(s)
M08(s)
M06(s)
M07(s)
M10(s)
M13(s)
M11(s)
M14(s)
M12(s)
M15(s)
M02(s)
M01(s)
Chemical Shift (ppm

5-(3-(cyclopropanecarboxamido)-4-fluorophenyl)-3-ureidothiophene-2-carboxamide (**59**)

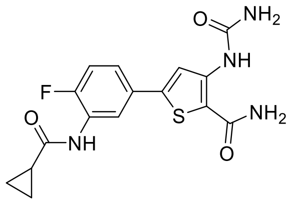

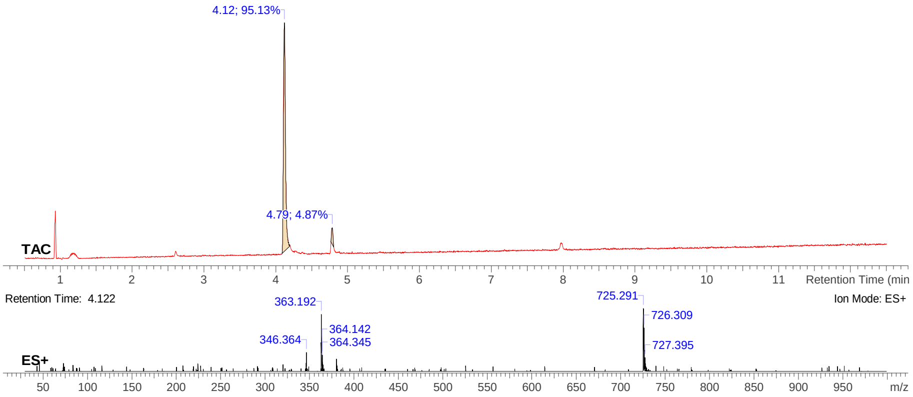

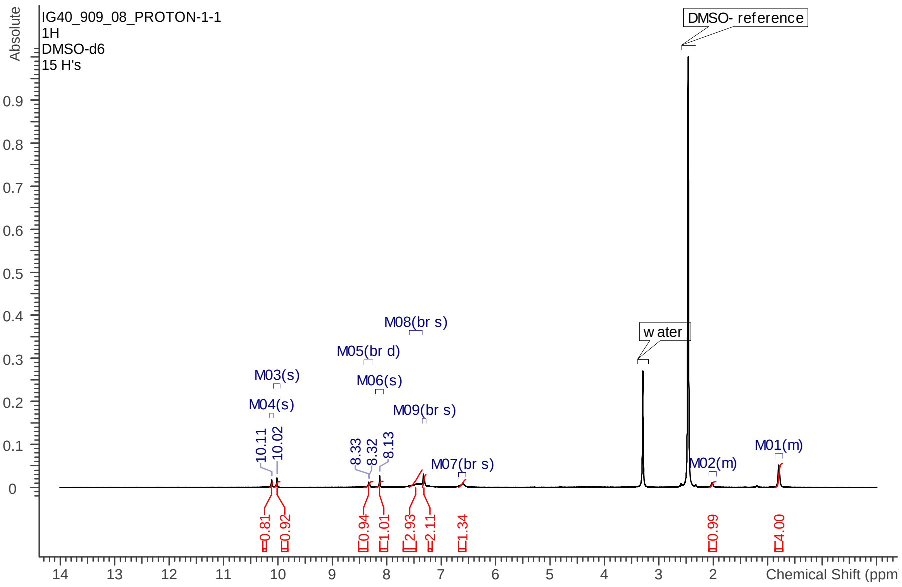

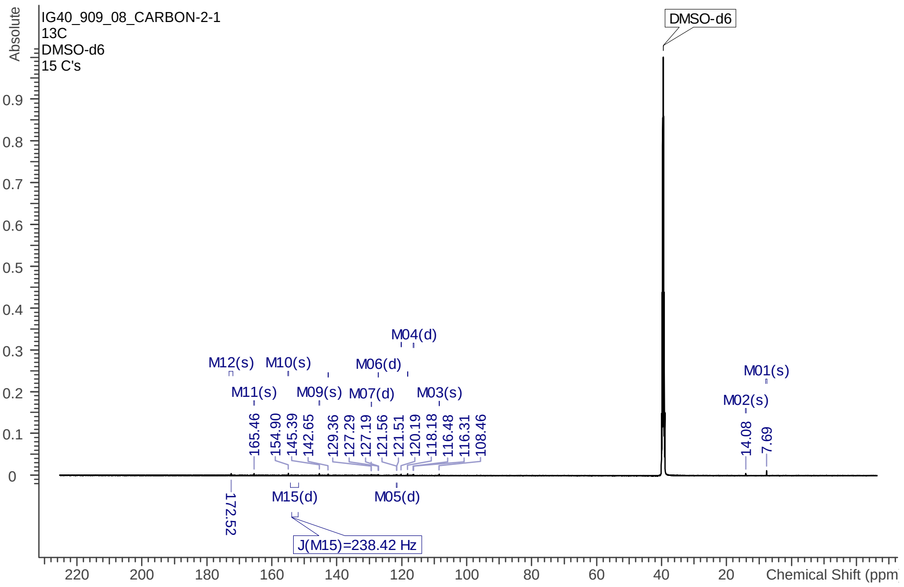

N-(4-(4'-oxo-3',4'-dihydro-1'*H*-spiro[cyclopentane-1,2'-thieno[3,2-*d*]pyrimidin]-6'-yl)pyridin-2-yl)cyclopropanecarboxamide (**60**)

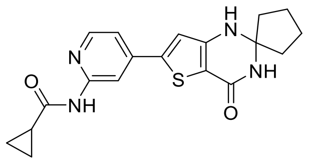

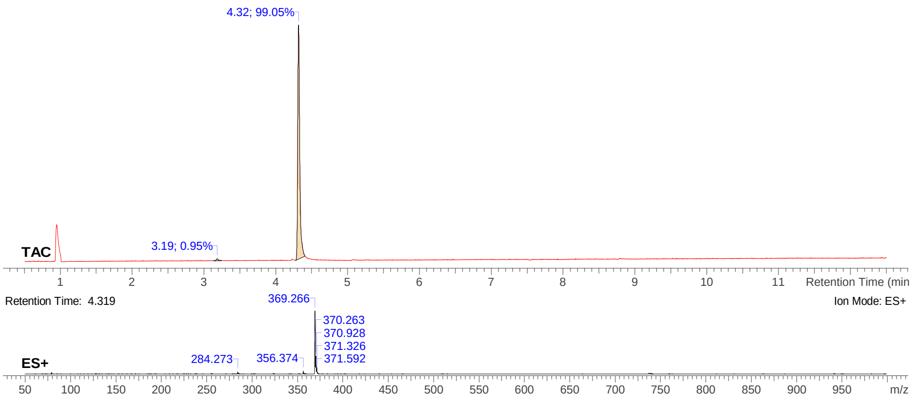

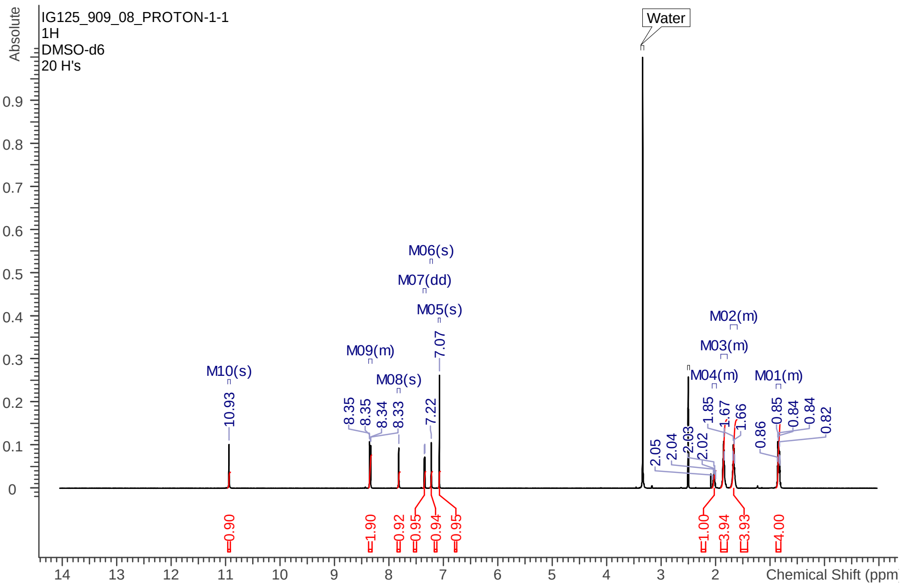
IG125_909_08_PROTON-1-1
1H
DMSO-d6
20 H's
Water
M10(s)
10.93
M09(m)
8.35
8.35
8.34
8.33
M08(s)
M07(dd)
M06(s)
M05(s)
7.22
7.07
M04(m)
M03(m)
M02(m)
M01(m)
2.05
2.04
2.03
2.02
1.85
1.67
1.66
0.86
0.85
0.84
0.84
0.82
0.90
1.90
0.92
0.95
0.94
0.95
1.00
3.94
3.93
4.00
Absolute
Chemical Shift (ppm

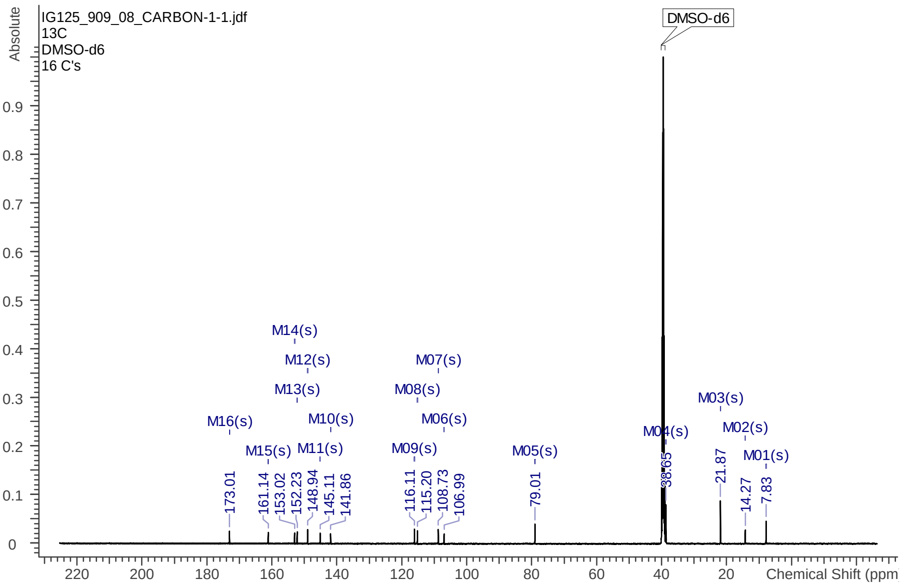
IG125_909_08_CARBON-1-1.jdf
13C
DMSO-d6
16 C's
DMSO-d6
M16(s)
173.01
M15(s)
161.14
M14(s)
M13(s)
M12(s)
153.02
152.23
148.94
M10(s)
M11(s)
145.11
141.86
M09(s)
116.11
115.20
M08(s)
M07(s)
M06(s)
108.73
106.99
M05(s)
79.01
M04(s)
38.65
M03(s)
21.87
M02(s)
14.27
M01(s)
7.83
Absolute
Chemical Shift (ppm

N-(4-(4'-oxo-3',4,4',5-tetrahydro-1'*H*,3*H*-spiro[furan-2,2'-thieno[3,2-*d*]pyrimidin]-6'-yl)pyridin-2-yl)cyclopropanecarboxamide (**61**)

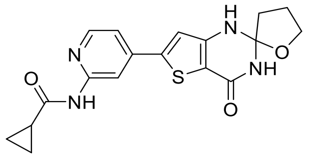

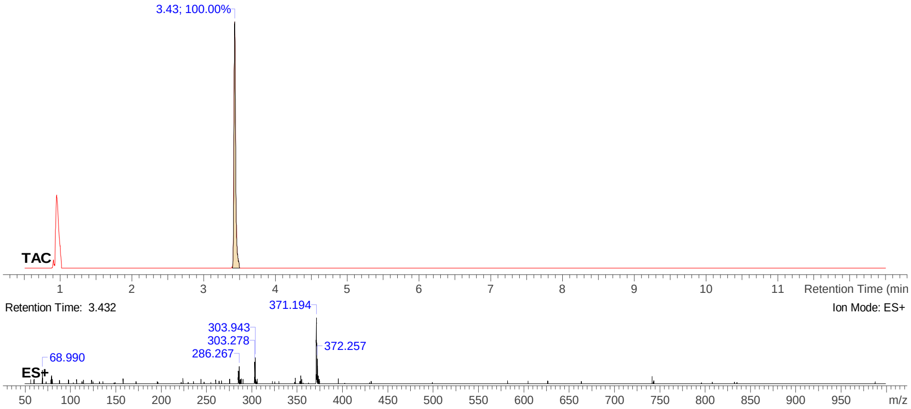

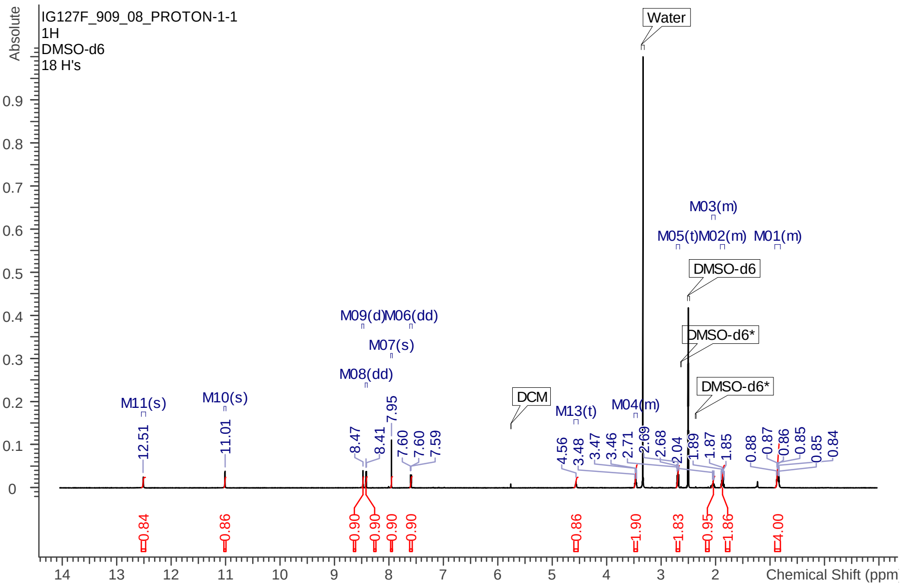

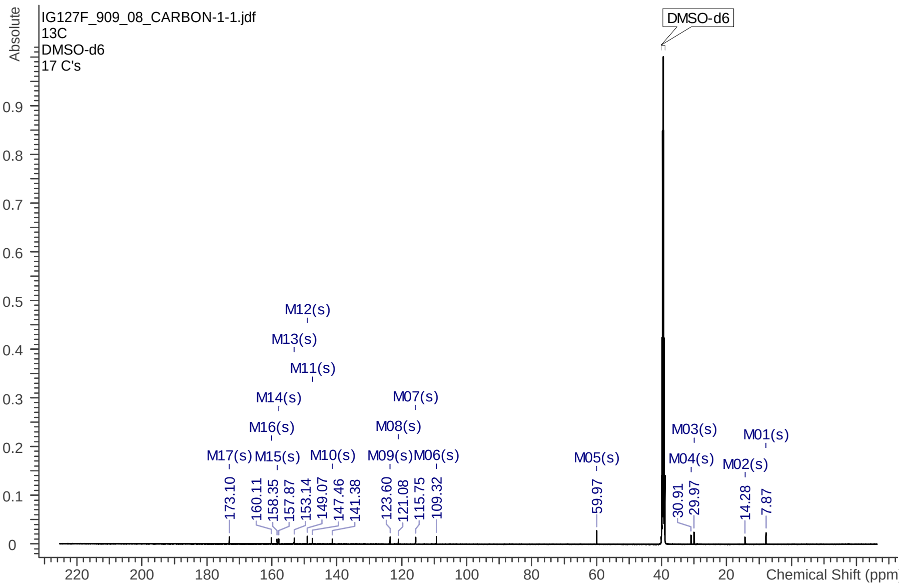

N-(4-(4'-oxo-3',4,4',5-tetrahydro-1'*H*,2*H*-spiro[furan-3,2'-thieno[3,2-*d*]pyrimidin]-6'-yl)pyridin-2-yl)cyclopropanecarboxamide (**62**)

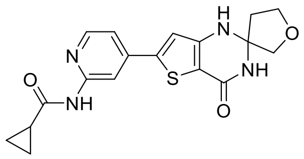

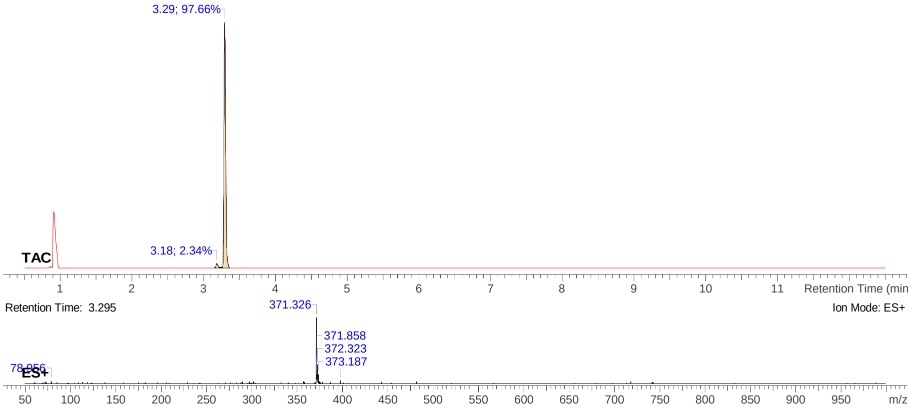

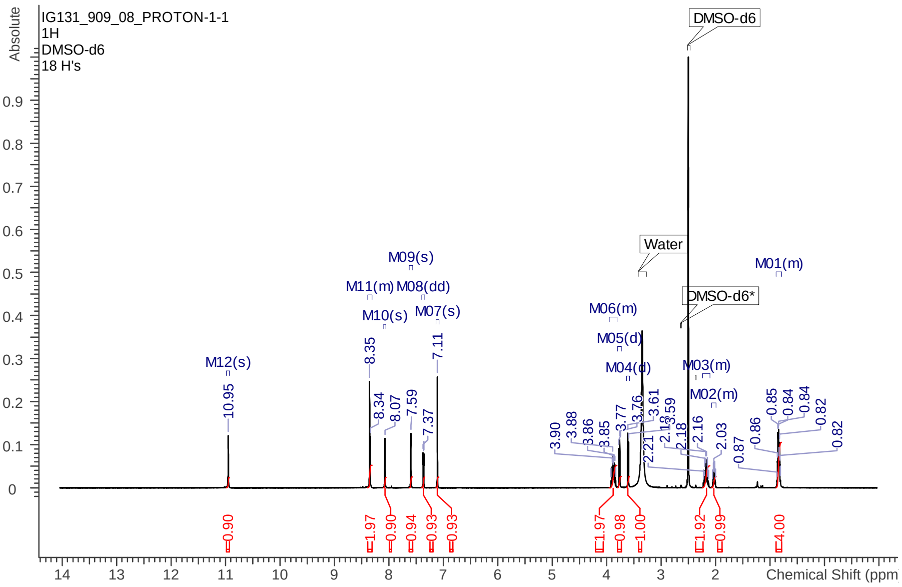
IG131_909_08_PROTON-1-1
1H
DMSO-d6
18 H's
Chemical Shift (ppm)

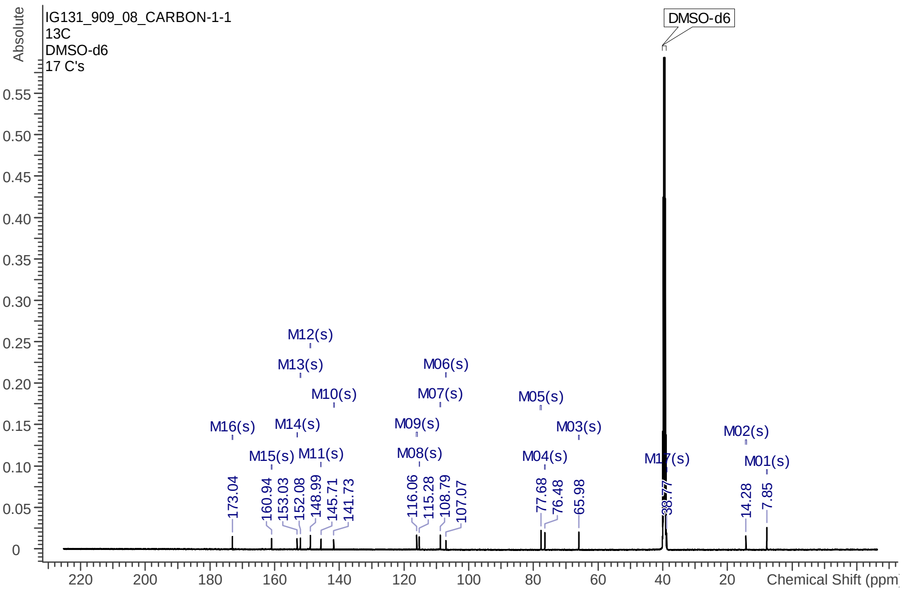
IG131_909_08_CARBON-1-1
13C
DMSO-d6
17 C's
Chemical Shift (ppm)
